# Supplementary material for: Systematic discovery of conservation states for single-nucleotide annotation of the human genome
Source: Commun Biol. 2019 Jul 2;2:248. doi: 10.1038/s42003-019-0488-1 (PMC6606595; doi:10.1038/s42003-019-0488-1)
Supplement: Supplementary file 1 — Supplementary Information [file 42003_2019_488_MOESM1_ESM.pdf]

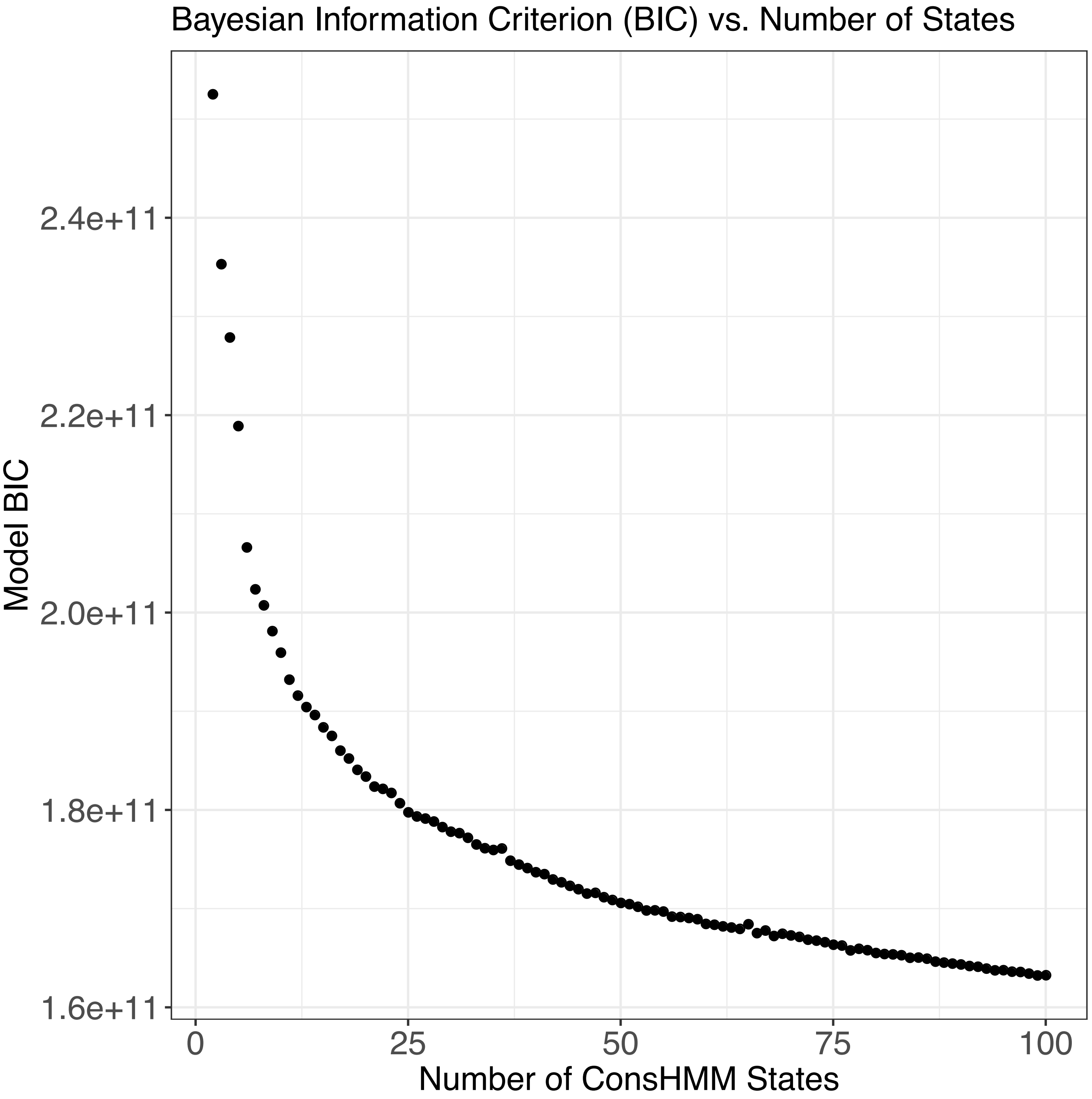

Supplementary Figure 1: BIC as a function of number of states in the model. The BIC criterion computed for models with each number of states from 2 to 100. For this criterion lower values correspond to preferred models.

State	Average posterior probability of state at positions assigned to state
1	1.00
2	0.99
3	0.99
4	0.98
5	0.96
6	0.96
7	0.97
8	0.92
9	0.93
10	0.94
11	0.98
12	0.97
13	0.97
14	0.95
15	0.92
16	0.92
17	0.95
18	0.94
19	0.97
20	0.98
21	0.98
22	0.96
23	0.97
24	0.98
25	0.97
26	0.97
27	0.96
28	0.98
29	0.97
30	0.98
31	0.97
32	0.97
33	0.98
34	0.98
35	0.97
36	0.96
37	0.97
38	0.97
39	0.97
40	0.97
41	0.96
42	0.95
43	0.97
44	0.96
45	0.97
46	0.97
47	0.93
48	0.95
49	0.96
50	0.96
51	0.95
52	0.93
53	0.95
54	0.98
55	0.97
56	0.96
57	0.96
58	0.96
59	0.95
60	0.95
61	0.96
62	0.96
63	0.96
64	0.97
65	0.98
66	0.99
67	0.98
68	0.98
69	0.97
70	0.95
71	0.94
72	0.96
73	0.98
74	0.99
75	0.99
76	0.99
77	1.00
78	1.00
79	0.99
80	0.98
81	0.98
82	0.99
83	0.99
84	0.99
85	0.99
86	1.00
87	0.97
88	0.98
89	0.95
90	0.99
91	0.98
92	1.00
93	1.00
94	1.00
95	0.99
96	1.00
97	1.00
98	1.00
99	1.00
100	1.00

Supplementary Figure 2: Average posterior probability of ConsHMM state assignments. The value listed for each state is the average posterior probability of that state for all bases in the genome assigned to that state.

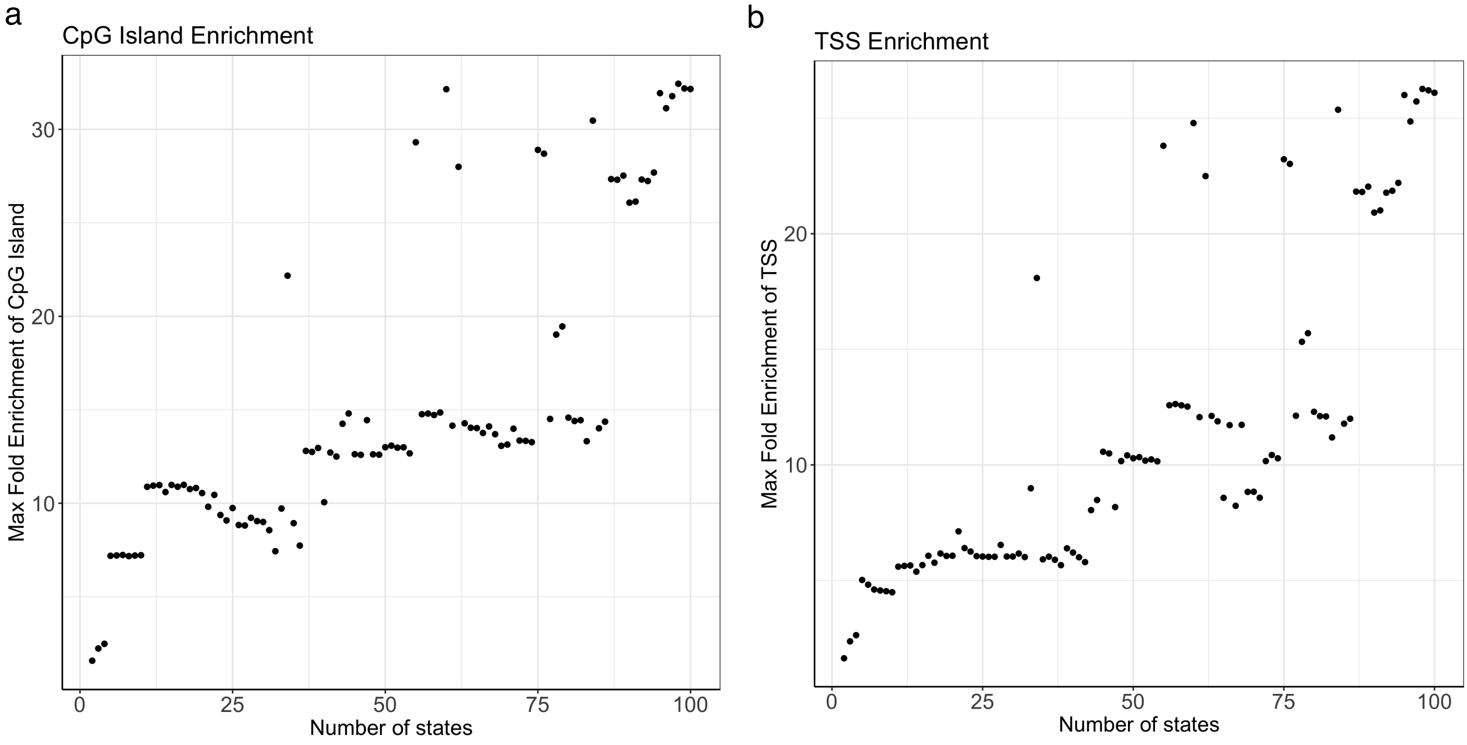

Supplementary Figure 3: Maximum CpG and TSS state enrichments as a function of number of states in the model. The figures show the maximum fold enrichment for **(a)** CpG islands and **(b)** TSS of any state in a model as a function of the number of states in the model. The figure shows that states with substantially higher enrichment for these annotations are only found consistently in models with a large number of states. There were isolated cases of models with a moderate number of states also exhibiting high enrichment. However, since similar enrichment levels were not captured in models with similar numbers of states this suggest the possibility that other biologically relevant states might be missing from these models.

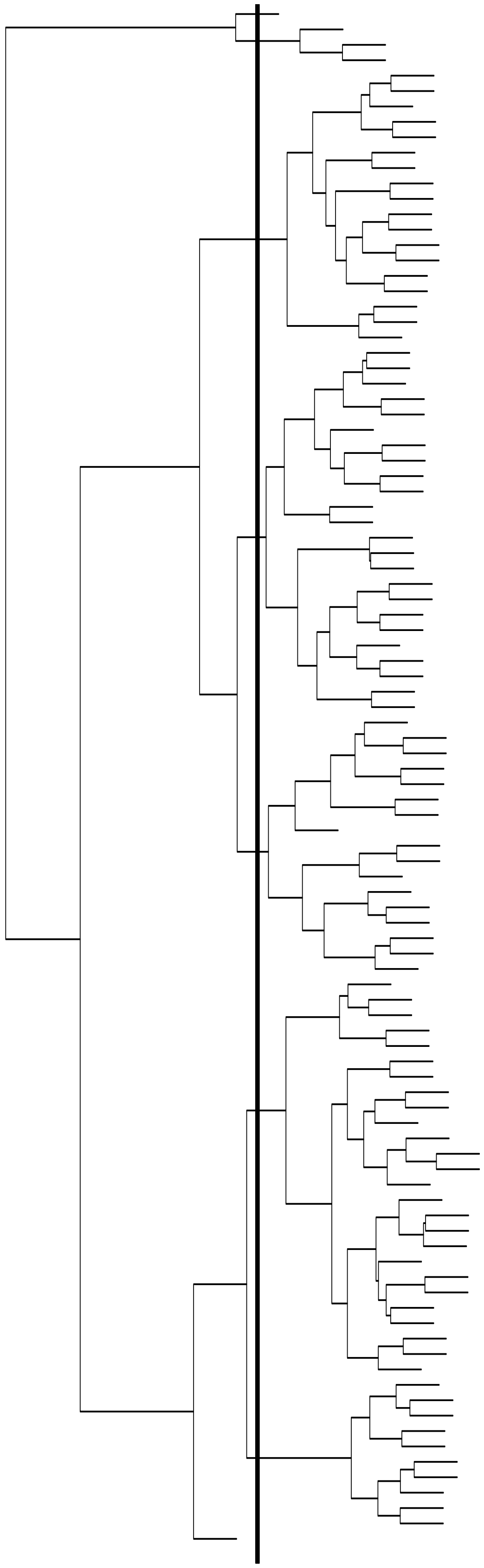

State	Group Mnemonic	Group Description
1	AM_allVert	High align and match frequency for all vertebrates
2 3 4	AM_nonMam	High align and matching for mammals and some non-mammals
5 6 7 8 9 10 11 12 13 14 15 16 17 18 19 20 21 22	AM_Mam	High align and match for mammals
23 24 25 26 27 28 29 30 31 32 33 34 35 36 37 38 39 40 41 42 43 44 45 46	AM_SMam	High align and match for mammals, but missing notable subsets
47 48 49 50 51 52 53 54 55 56 57 58 59 60 61 62 63	A_SMam	High align frequencies for mammals, but low match frequencies
64 65 66 67 68 69 70 71 72 73 74 75 76 77 78 79 80 81 82 83 84 85 86 87 88 89	AM_Prim	High align and match frequencies for primates
90 91 92 93 94 95 96 97 98 99	AM_SPrim	High align and match frequencies for a few primates
100	artifact	Putative artifact

Suplementary Figure 4: Hierarchical clustering and grouping of conservation states. The dendrogram on left displays a hierarchical clustering of the states based on the values in **Fig. 2a**, with the leaves ordered based on optimal leaf ordering[1].The thick black line indicates where the dendrogram was cut to form the eight major groups of conservation states, each receiving a different color shown on right. To the right of the state numbers are state group abbreviations from **Fig. 2a**. To the right of the state labels is a high level description of the general patterns of the parameters of the state groups. Notable enrichments associated with specific states are summarized in **Supplementary Data 1**.

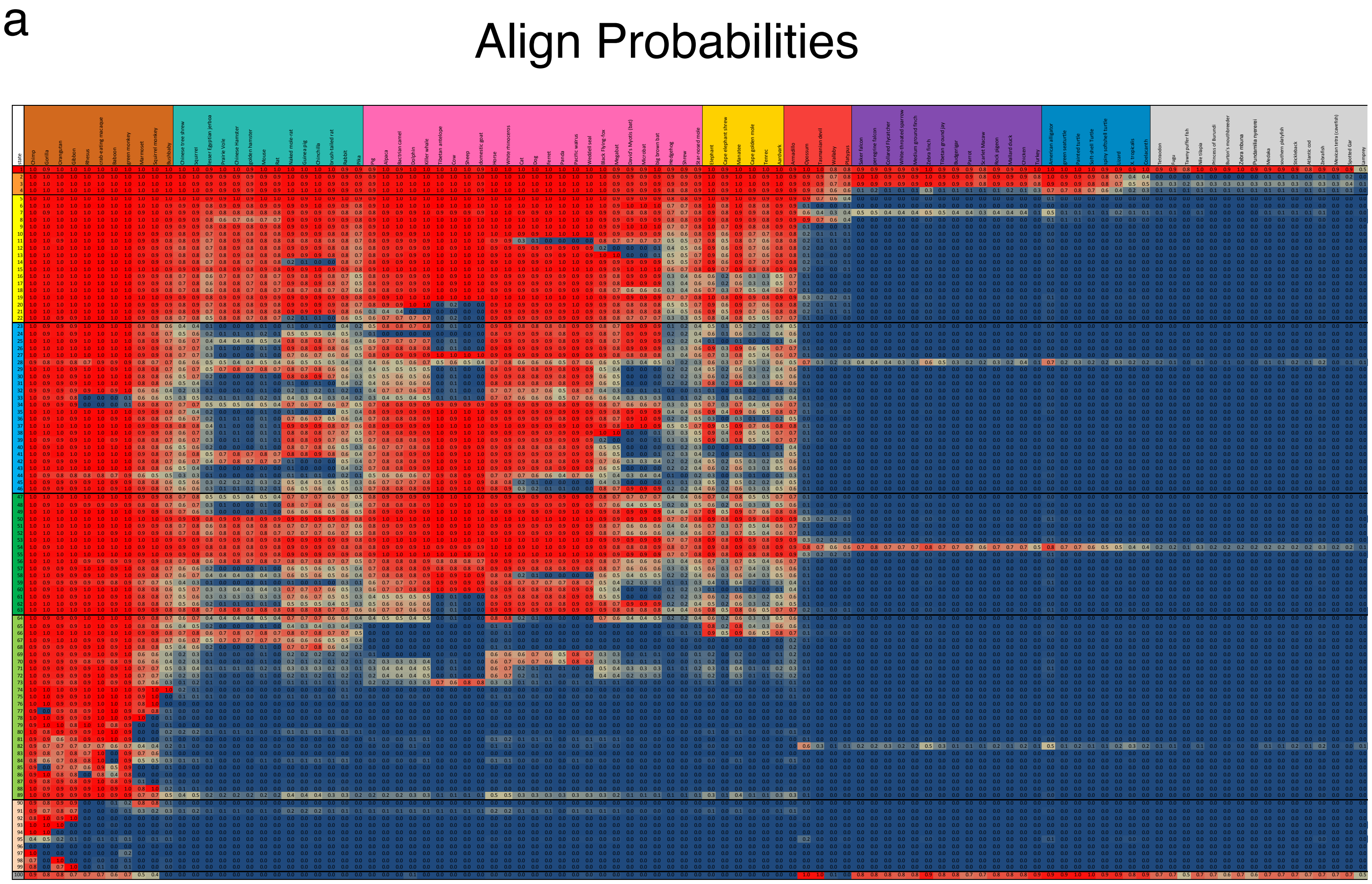

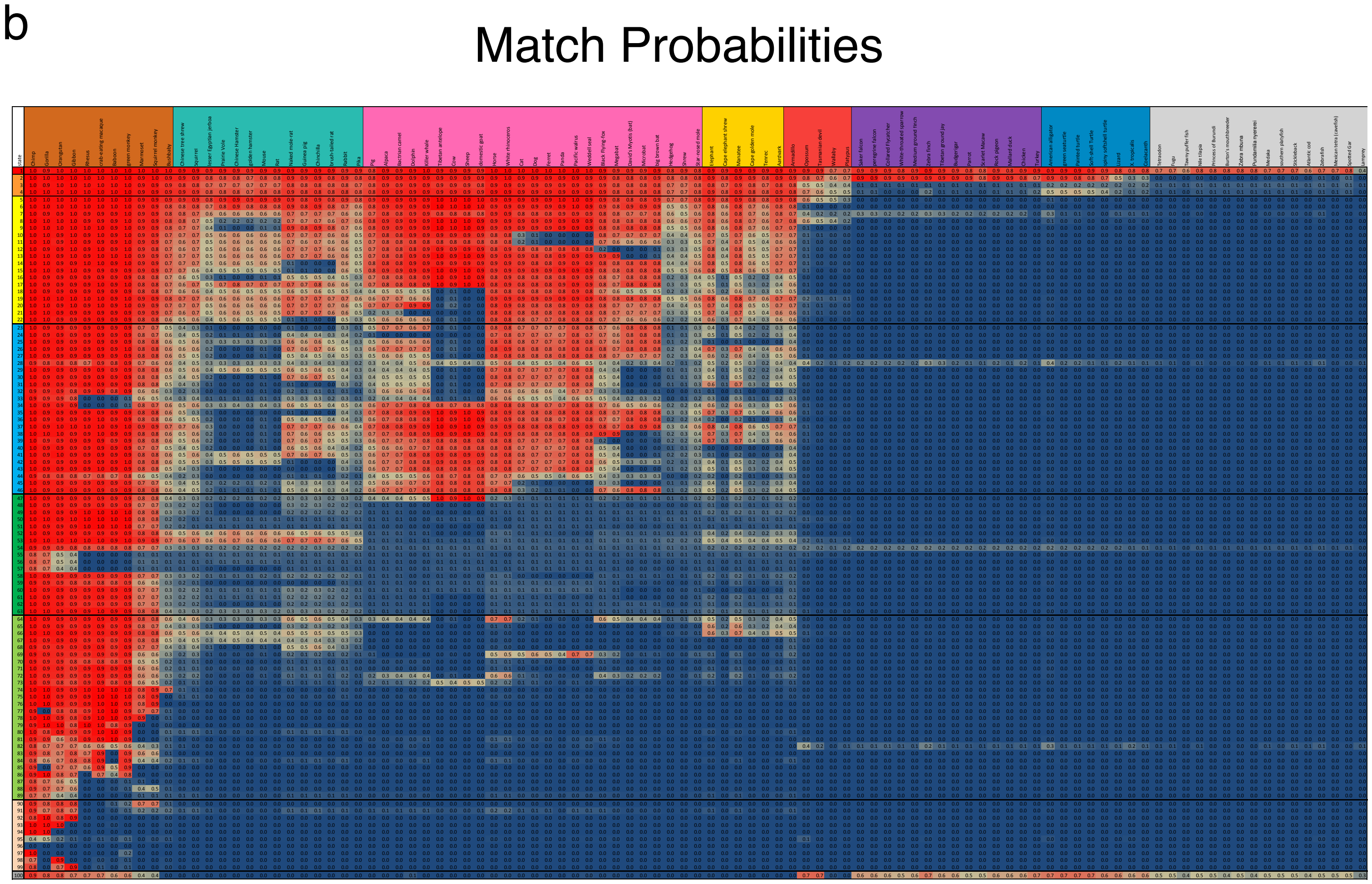

Supplementary Figure 5: Representation of the emission parameters. This is a more detailed view of the representation of the emission parameters shown in the heatmap in **Fig. 2a**. In this figure the actual probabilities values and the individual species names are also displayed.

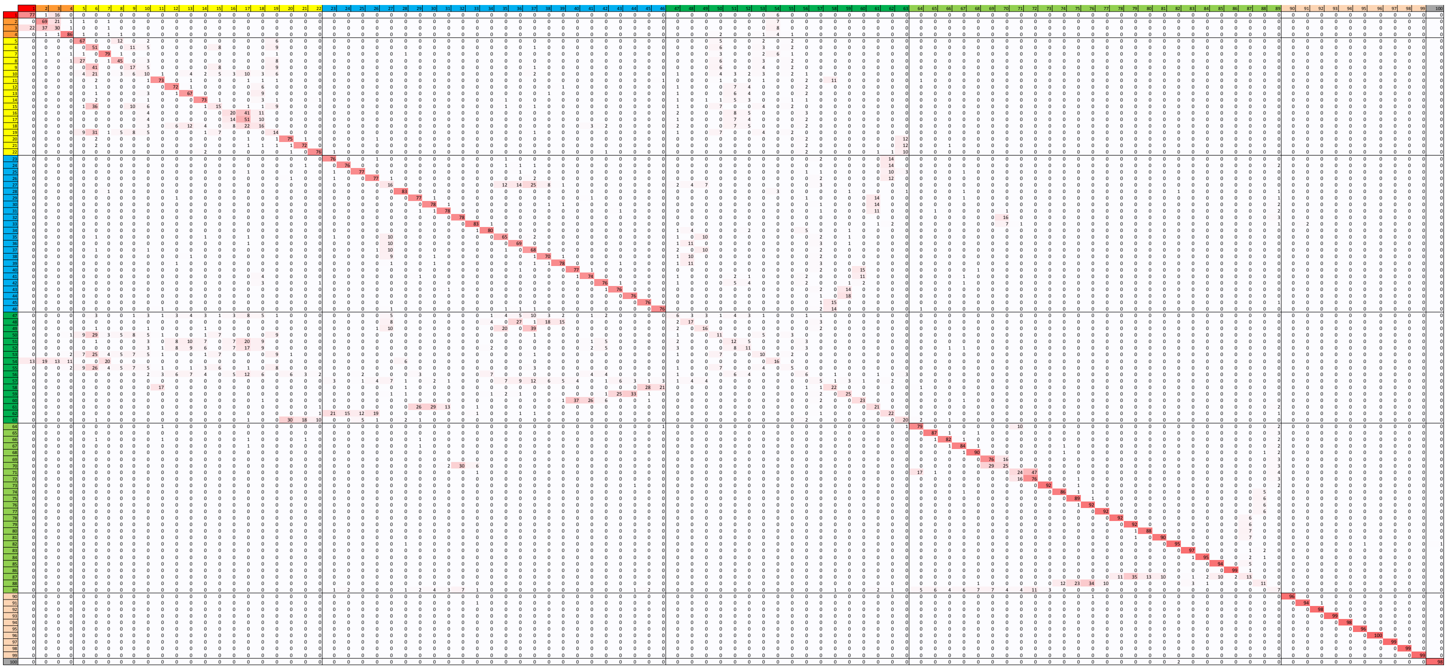

Supplementary Figure 6: Conservation state model transition probabilities. The figure displays the conservation state model transition probabilities. The values correspond to the probability when in the state of the row to transition to the state of the column at the next base. Probabilities are displayed multiplied by 100, rounded to the nearest integer and shaded based on their value, with darker red corresponding to greater transition probabilities. Transition probabilities along the diagonal show the probability of remaining in the state at a neighboring position, which were often the highest values for some states. For states associated with low matching probabilities relative to the alignment probabilities such as the A_SMam subgroup (states 47-63) the probability of remaining in the same state was low. Transition probabilities to stay in the same state were highest in some states only showing substantial alignability at most within primates, thus the model can use spatial information through these transition probabilities to better differentiate instances of states with relatively similar emission probabilities.

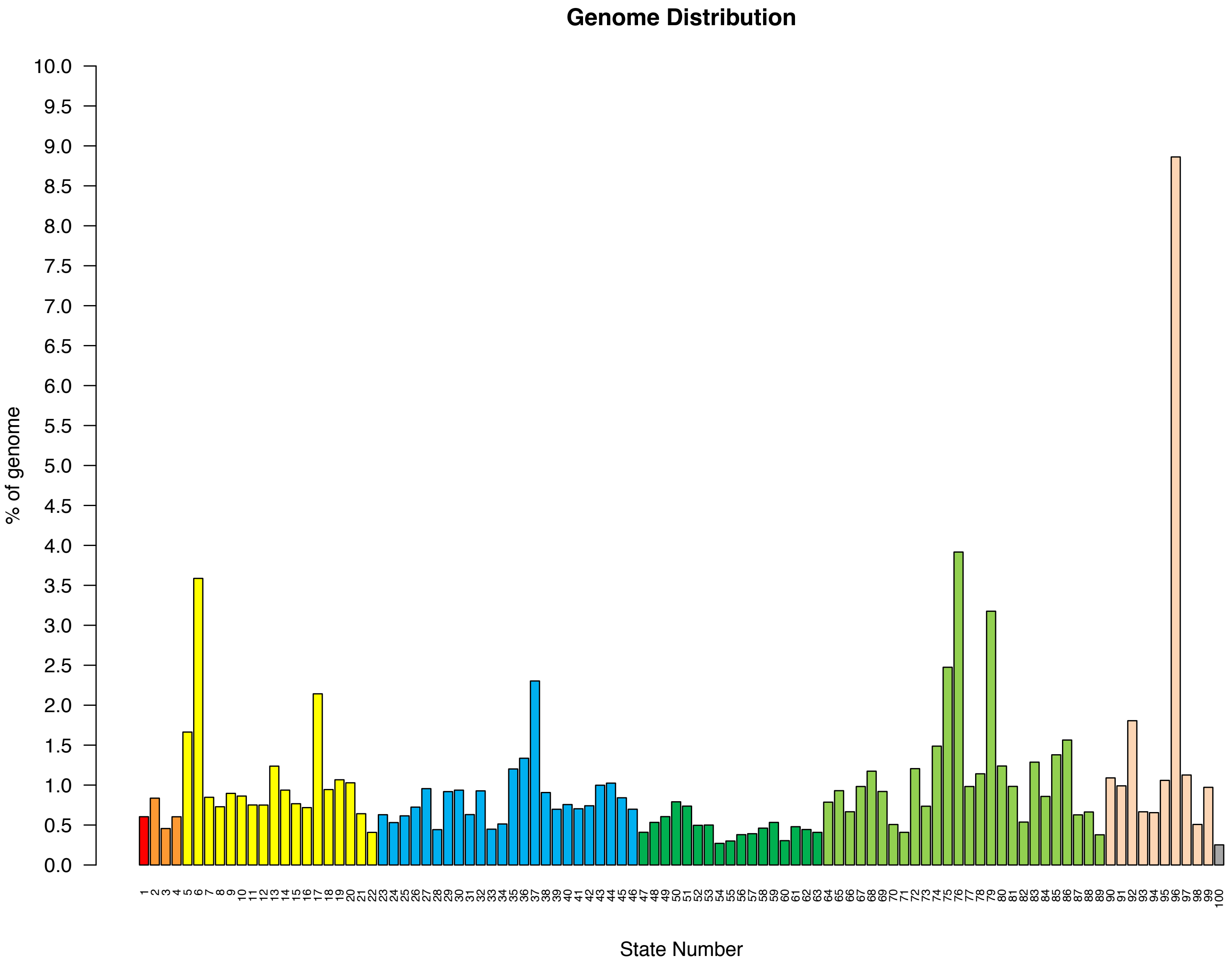

Supplementary Figure 7: Distribution of the genome in each state. The graph displays the percent of the genome assigned to each conservation state. The median state coverage was 0.76% of the genome. All states except state 96 were in the range of 0.25% to 3.92% of the genome. State 96 was the largest state covering 8.86% of the genome and was associated with assembly gaps (**Supplementary Fig. 8**).

	Genome %	TSS of protein coding genes	TSS of pseudogenes	TES of protein coding genes	TES of pseudogenes	CDS	Exons of protein coding genes	Exons of pseudogenes	UTR	CpG Island	PhastCons elements	Repeat elements	Median non-exonic DHS	Assembly gaps
1	0.60	1.78	2.37	1.98	2.06	71.49	28.14	7.36	12.34	12.54	17.94	0.01	2.84	0.00
2	0.84	2.37	0.77	7.00	0.61	9.15	7.85	1.23	9.00	1.53	14.19	0.02	3.07	0.00
3	0.46	1.85	0.80	5.10	0.94	30.08	14.81	4.70	9.98	5.45	9.39	0.02	2.62	0.00
4	0.60	6.88	1.95	5.30	1.79	16.20	8.98	2.92	7.06	5.46	10.45	0.03	3.97	0.00
5	1.66	5.99	0.30	5.93	0.23	1.83	3.28	0.27	4.54	2.75	9.08	0.06	4.09	0.00
6	3.59	1.73	0.11	2.19	0.10	0.25	1.19	0.09	1.62	0.94	2.92	0.36	2.49	0.00
7	0.85	6.76	0.91	6.72	0.96	2.30	4.76	0.69	5.39	7.10	2.09	0.10	2.14	0.00
8	0.73	3.30	0.26	4.55	0.36	1.19	2.88	0.34	3.84	2.16	2.75	0.05	2.83	0.00
9	0.90	1.28	0.06	1.95	0.11	0.19	1.14	0.08	1.53	0.91	1.01	0.37	2.13	0.00
10	0.86	1.72	0.08	2.25	0.14	0.30	1.30	0.12	1.69	1.11	0.65	0.40	2.02	0.00
11	0.75	1.23	0.37	1.20	0.31	0.21	1.05	0.31	1.29	0.93	0.64	0.49	1.50	0.00
12	0.75	0.91	0.22	1.00	0.20	0.15	0.73	0.25	0.92	0.73	0.84	0.46	1.42	0.00
13	1.24	1.03	0.11	1.03	0.06	0.13	0.74	0.09	0.94	0.83	1.05	0.46	1.57	0.00
14	0.94	1.65	0.20	1.45	0.11	0.19	1.22	0.15	1.57	1.43	0.94	0.43	2.16	0.00
15	0.77	1.75	0.13	1.99	0.09	0.24	1.30	0.10	1.72	1.25	0.61	0.36	2.20	0.00
16	0.72	0.77	0.15	0.83	0.09	0.09	0.61	0.14	0.71	0.60	0.19	0.64	1.28	0.00
17	2.14	0.62	0.21	0.83	0.15	0.09	0.58	0.15	0.69	0.44	0.77	0.64	1.28	0.00
18	0.94	0.71	0.17	0.89	0.14	0.11	0.69	0.14	0.82	0.70	0.18	0.59	1.41	0.00
19	1.07	2.24	0.14	2.88	0.08	0.54	1.71	0.15	2.26	1.35	1.22	0.29	2.42	0.00
20	1.03	3.31	0.16	1.14	0.14	0.26	1.12	0.16	1.48	3.29	1.00	0.48	1.73	0.00
21	0.64	1.00	0.22	1.21	0.17	0.22	1.05	0.16	1.30	0.96	0.72	0.46	1.51	0.00
22	0.41	1.79	0.21	0.75	0.23	0.15	0.82	0.19	1.00	2.25	0.40	0.63	1.47	0.00
23	0.63	0.48	0.31	0.68	0.22	0.07	0.45	0.18	0.51	0.66	0.37	0.92	1.00	0.00
24	0.53	0.45	0.10	0.58	0.14	0.06	0.42	0.19	0.48	0.40	0.28	0.83	0.96	0.00
25	0.61	0.49	0.20	0.46	0.11	0.05	0.36	0.12	0.39	0.40	0.31	1.01	0.91	0.00
26	0.72	0.63	0.14	0.67	0.14	0.06	0.49	0.18	0.57	0.55	0.44	0.68	1.06	0.00
27	0.96	0.71	0.09	0.96	0.13	0.09	0.67	0.15	0.79	0.59	0.20	0.62	1.34	0.00
28	0.44	29.03	14.52	5.03	14.52	7.34	6.79	14.19	6.59	32.16	3.27	0.20	4.13	0.00
29	0.92	0.63	0.20	0.39	0.13	0.05	0.35	0.12	0.40	0.55	0.28	0.81	0.91	0.00
30	0.94	0.21	0.20	0.34	0.09	0.03	0.26	0.12	0.30	0.19	0.29	0.86	0.79	0.00
31	0.63	0.51	0.21	0.55	0.17	0.08	0.37	0.22	0.42	0.62	0.31	0.80	0.90	0.00
32	0.93	0.37	0.24	0.43	0.24	0.06	0.28	0.26	0.33	0.48	0.36	1.33	0.67	0.00
33	0.45	0.92	0.83	0.52	0.60	0.09	0.29	0.63	0.33	0.78	0.41	1.05	0.63	0.00
34	0.51	0.71	0.56	0.65	0.67	0.13	0.48	0.57	0.55	0.50	0.65	0.62	0.88	0.00
35	1.20	0.99	0.26	1.22	0.15	0.14	0.92	0.19	1.11	1.01	0.64	0.56	1.55	0.00
36	1.34	0.34	0.19	0.58	0.13	0.04	0.35	0.13	0.40	0.20	0.47	0.89	0.95	0.00
37	2.30	0.61	0.13	1.11	0.11	0.09	0.72	0.12	0.89	0.47	0.89	0.51	1.46	0.00
38	0.91	0.58	0.15	0.60	0.13	0.08	0.47	0.11	0.57	0.36	0.56	0.63	1.12	0.00
39	0.70	0.41	0.24	0.58	0.31	0.08	0.40	0.27	0.47	0.29	0.45	0.65	0.96	0.00
40	0.76	0.31	0.16	0.27	0.12	0.03	0.21	0.14	0.24	0.14	0.33	1.03	0.76	0.00
41	0.70	0.39	0.10	0.57	0.19	0.05	0.36	0.14	0.41	0.32	0.38	0.84	0.96	0.00
42	0.74	0.82	0.26	0.66	0.18	0.12	0.54	0.19	0.60	0.67	0.36	0.75	1.20	0.00
43	1.00	0.27	0.17	0.51	0.11	0.07	0.39	0.14	0.45	0.40	0.39	0.77	1.07	0.00
44	1.02	0.68	0.66	0.65	0.55	0.13	0.39	0.64	0.44	0.64	0.46	1.11	0.78	0.00
45	0.84	0.48	0.37	0.57	0.22	0.10	0.38	0.29	0.42	0.34	0.30	0.87	0.86	0.00
46	0.70	0.45	0.26	0.63	0.26	0.10	0.48	0.33	0.51	0.33	0.38	0.80	0.98	0.00
47	0.41	1.28	0.29	1.29	0.27	0.18	0.93	0.19	1.10	1.18	0.05	0.53	1.59	0.00
48	0.53	0.43	0.11	0.64	0.16	0.08	0.43	0.20	0.49	0.42	0.14	0.71	1.03	0.00
49	0.61	0.92	0.16	1.13	0.18	0.13	0.90	0.18	1.08	0.99	0.23	0.48	1.54	0.00
50	0.79	3.11	0.16	3.03	0.17	0.74	1.92	0.21	2.48	1.89	1.22	0.26	2.38	0.00
51	0.74	1.05	0.26	0.89	0.14	0.13	0.75	0.17	0.90	0.96	0.21	0.54	1.45	0.00
52	0.50	1.14	0.18	1.00	0.22	0.13	0.81	0.17	0.97	1.06	0.14	0.55	1.49	0.00
53	0.50	2.64	0.20	3.48	0.19	0.83	2.09	0.21	2.62	2.05	0.75	0.27	2.41	0.00
54	0.27	3.88	1.62	5.53	3.13	20.62	11.07	6.63	7.67	6.87	4.24	0.04	2.15	0.00
55	0.30	3.12	0.15	2.82	0.29	0.80	1.87	0.31	2.37	2.03	1.44	0.26	2.31	0.00
56	0.38	1.55	0.21	1.10	0.27	0.15	0.72	0.27	0.86	1.27	0.22	0.56	1.33	0.00
57	0.39	0.80	0.39	0.84	0.17	0.12	0.57	0.27	0.66	0.83	0.17	0.67	1.17	0.00
58	0.46	1.08	0.27	0.95	0.29	0.18	0.68	0.37	0.75	0.87	0.10	0.69	1.15	0.00
59	0.53	0.85	0.62	0.80	0.51	0.16	0.56	0.51	0.62	1.20	0.11	0.85	1.08	0.00
60	0.30	0.32	0.14	0.56	0.22	0.06	0.34	0.17	0.37	0.41	0.08	0.90	0.90	0.00
61	0.48	0.52	0.14	0.40	0.15	0.06	0.37	0.15	0.42	0.61	0.07	0.76	0.92	0.00
62	0.44	0.70	0.20	0.52	0.13	0.08	0.50	0.20	0.55	0.80	0.10	0.79	1.08	0.00
63	0.41	2.79	0.30	1.18	0.11	0.29	1.19	0.25	1.45	3.15	0.19	0.49	1.63	0.00
64	0.79	0.79	0.23	0.56	0.23	0.07	0.42	0.18	0.47	0.61	0.30	0.82	0.91	0.00
65	0.93	0.49	0.19	0.54	0.17	0.07	0.48	0.12	0.54	0.75	0.16	0.81	0.91	0.00
66	0.67	1.21	0.19	1.07	0.13	0.15	0.93	0.16	1.11	1.28	0.29	0.54	1.33	0.00
67	0.98	0.85	0.62	0.77	0.46	0.13	0.73	0.25	0.81	1.22	0.16	1.17	0.97	0.00
68	1.17	0.30	0.31	0.38	0.16	0.04	0.33	0.18	0.35	0.29	0.11	1.46	0.60	0.00
69	0.92	0.25	0.36	0.34	0.23	0.05	0.26	0.21	0.29	0.34	0.26	1.30	0.63	0.00
70	0.51	0.46	0.29	0.50	0.32	0.08	0.36	0.31	0.40	0.89	0.09	1.20	0.74	0.00
71	0.41	0.78	0.54	0.56	0.38	0.12	0.48	0.33	0.51	1.02	0.08	1.08	0.84	0.00
72	1.21	0.43	0.36	0.58	0.35	0.08	0.34	0.32	0.37	0.52	0.30	1.27	0.68	0.00
73	0.74	0.48	0.65	0.62	0.69	0.18	0.49	0.58	0.52	0.42	0.18	1.53	0.56	0.00
74	1.49	0.37	0.40	0.56	0.28	0.06	0.48	0.15	0.55	0.34	0.12	1.67	0.78	0.00
75	2.47	0.22	1.34	0.31	0.76	0.05	0.46	0.40	0.51	0.15	0.19	1.80	0.45	0.00
76	3.92	0.15	0.37	0.22	0.27	0.03	0.22	0.18	0.25	0.03	0.14	2.00	0.48	0.00
77	0.98	0.27	0.32	0.35	0.21	0.04	0.37	0.17	0.42	0.18	0.11	1.95	0.36	0.00
78	1.14	0.24	0.51	0.24	0.38	0.03	0.24	0.25	0.28	0.21	0.14	1.93	0.49	0.00
79	3.18	0.14	0.29	0.22	0.25	0.02	0.17	0.22	0.21	0.06	0.11	2.02	0.39	0.00
80	1.24	0.22	0.31	0.39	0.20	0.04	0.39	0.17	0.44	0.19	0.16	1.81	0.37	0.00
81	0.98	0.29	1.91	0.42	1.13	0.04	0.26	0.76	0.30	0.64	0.39	1.82	0.40	0.00
82	0.54	3.07	57.15	2.36	48.00	1.24	1.71	26.12	1.69	10.08	3.57	0.50	0.86	0.00
83	1.29	0.18	0.37	0.23	0.33	0.03	0.15	0.45	0.17	0.12	0.09	2.00	0.27	0.00
84	0.86	0.43	1.06	0.58	0.94	0.16	0.34	1.08	0.36	0.72	0.26	1.64	0.29	0.00
85	1.38	0.13	0.27	0.21	0.24	0.03	0.15	0.27	0.19	0.15	0.06	2.02	0.23	0.00
86	1.56	0.08	0.25	0.11	0.23	0.01	0.10	0.20	0.11	0.06	0.08	2.07	0.21	0.00
87	0.63	0.28	0.61	0.37	0.38	0.04	0.26	0.34	0.31	0.31	0.06	1.92	0.38	0.00
88	0.66	0.32	0.85	0.26	0.47	0.04	0.36	0.26	0.41	0.20	0.06	1.85	0.50	0.00
89	0.38	1.14	0.52	0.63	0.43	0.15	0.53	0.40	0.58	1.31	0.09	1.00	0.88	0.00
90	1.09	0.17	0.46	0.27	0.41	0.03	0.17	0.29	0.19	0.14	0.13	1.92	0.34	0.00
91	0.99	0.54	0.93	0.41	0.62	0.08	0.31	0.66	0.35	0.50	0.24	1.55	0.40	0.00
92	1.81	0.13	0.36	0.19	0.32	0.02	0.13	0.24	0.14	0.09	0.07	2.02	0.25	0.00
93	0.67	0.21	0.38	0.14	0.33	0.02	0.14	0.25	0.17	0.08	0.04	2.02	0.29	0.00
94	0.65	0.23	0.33	0.22	0.32	0.03	0.12	0.34	0.15	0.10	0.05	2.00	0.21	0.00
95	1.06	0.51	3.78	0.46	3.02	0.16	0.29	2.41	0.32	2.04	1.52	1.64	0.30	0.00
96	8.86	0.02	0.03	0.02	0.03	0.00	0.01	0.03	0.01	0.01	0.00	0.30	0.02	11.28
97	1.13	0.17	0.42	0.14	0.27	0.02	0.07	0.38	0.08	0.08	0.03	1.99	0.11	0.00
98	0.51	0.07	0.13	0.12	0.14	0.01	0.07	0.26	0.09	0.07	0.02	2.06	0.22	0.00
99	0.97	0.07	0.20	0.13	0.21	0.01	0.10	0.20	0.13	0.11	0.04	2.04	0.17	0.00
100	0.25	0.78	98.15	1.21	147.04	4.02	2.09	176.81	1.32	4.24	15.51	0.02	0.63	0.00
Base	100	6.30E-04	4.40E-04	6.30E-04	4.40E-04	1.13	2.98	0.38	1.58	0.68	5.17	46.72	0.72	7.57

Supplementary Fig. 8: Conservation state enrichment values. The first column displays the state number followed by the percentage of genome in each state, and then the values of the fold enrichments represented in the heatmap in **Fig. 2b** along with additional columns for: enrichments for TES of protein coding genes, TES of pseudogenes, coding sequence (CDS), untranslated regions (UTRs), and assembly gaps. The last row gives the % of the genome covered by the annotation in each column. In the case of median non-exonic DHS, the last row is the median genome coverage across the DNase I experiments.

State assignment using original model	Probability of assignment to same state using uniform transition probabilities
1	0.99
2	0.98
3	0.98
4	0.91
5	0.91
6	0.87
7	0.86
8	0.84
9	0.93
10	0.97
11	0.92
12	0.88
13	0.88
14	0.85
15	0.95
16	0.85
17	0.85
18	0.91
19	0.93
20	0.92
21	0.93
22	0.91
23	0.92
24	0.94
25	0.92
26	0.92
27	0.94
28	0.90
29	0.91
30	0.93
31	0.93
32	0.90
33	0.93
34	0.94
35	0.92
36	0.89
37	0.88
38	0.92
39	0.89
40	0.88
41	0.86
42	0.84
43	0.91
44	0.84
45	0.92
46	0.91
47	0.97
48	0.78
49	0.84
50	0.91
51	0.90
52	0.91
53	0.93
54	0.95
55	0.96
56	0.87
57	0.93
58	0.85
59	0.87
60	0.78
61	0.88
62	0.86
63	0.90
64	0.92
65	0.92
66	0.96
67	0.95
68	0.92
69	0.92
70	0.90
71	0.92
72	0.87
73	0.87
74	0.95
75	0.92
76	0.99
77	0.97
78	0.97
79	0.99
80	0.91
81	0.93
82	0.94
83	0.96
84	0.90
85	0.97
86	0.95
87	0.99
88	0.96
89	0.93
90	0.97
91	0.91
92	0.99
93	0.99
94	1.00
95	0.93
96	1.00
97	1.00
98	1.00
99	0.99
100	0.98

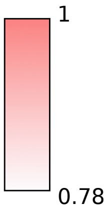

Supplementary Figure 9: Comparison of state annotations from the learned model versus the learned model except with uniform probabilities. The first column shows the state IDs. The second column displays for each state the fraction of bases annotated to the state using the learned model that were also assigned when using the learned model except with uniform transition probabilities. For all states the majority of bases were assigned to the same state when using the model with uniform transition probabilities with the fraction ranging between 0.78 and 1.00.

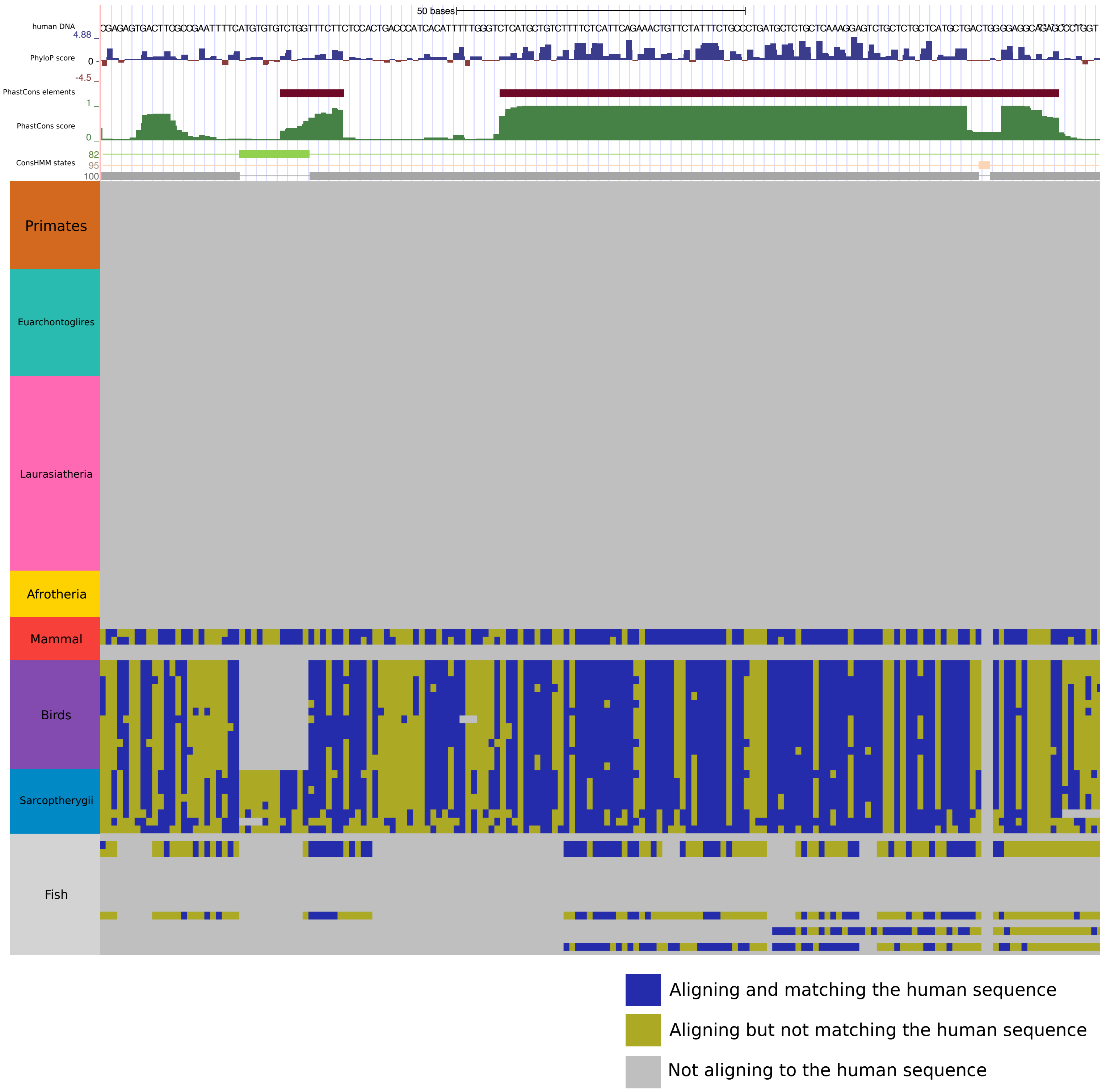

Supplementary Figure 10: Illustration of conservation state assignments at an additional locus. Similar to **Fig. 1b**, but illustrating the conservation state assignment at a different locus: chr22:20,673,589-20,673,761. The top tracks are the DNA sequence, the PhyloP score, PhastCons elements, and then PhastCons score. Below this set of tracks are the conservation state assignments with only the states assigned to at least one nucleotide in the locus shown. Below the conservation state assignments is a color encoding of the input multiple sequence alignment. The major clade of species as annotated on the UCSC genome browser[2] are labeled and ordered based on divergence from human. The figure is an example of positions with high constraint scores from PhyloP and PhastCons, while the multiple sequence alignment lacks alignment to most mammals, which is suggestive of alignment artifacts. ConsHMM states 82 and 100 capture the pattern of non-mammalian vertebrates aligning and/or matching the human genome, without most mammals. State 95 captures the pattern of all species having low alignment and matching probabilities and relatively proximal to states with higher probabilities of alignment and matching (**Supplementary Figs. 5** and **6**).

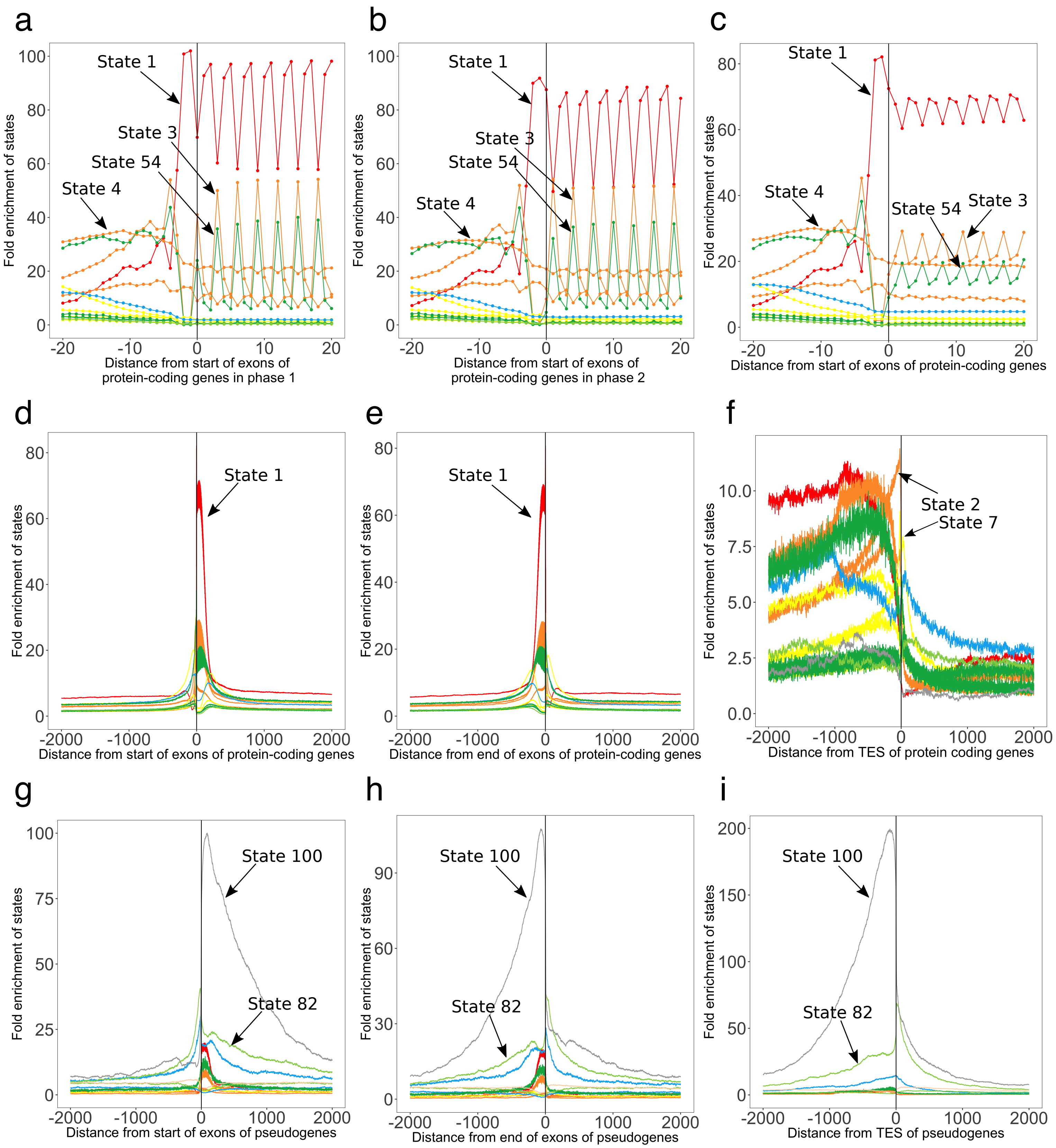

Supplementary Figure 11: Additional conservation state positional enrichments. The figure shows additional positional enrichment plots similar to what was shown in **Fig. 3**. These additional enrichment plots include enrichments relative to start of exons of protein coding genes for **(a)** phase 1 and **(b)** phase 2 exons, **(c)** all exons, and a zoomed out view of enrichments relative to **(d)** the start and **(e)** end of all exons of protein coding genes. Also shown are enrichment plots relative to **(f)** TES of protein coding genes, **(g)** start and **(h)** end of exons of pseudogenes as well as **(i)** TES of pseudogenes. Enrichments were computed relative to a genome-wide background. The subset of states included in the figure was composed of the states that had at least a 3 fold enrichment at some position within +/-2kb from the anchor point.

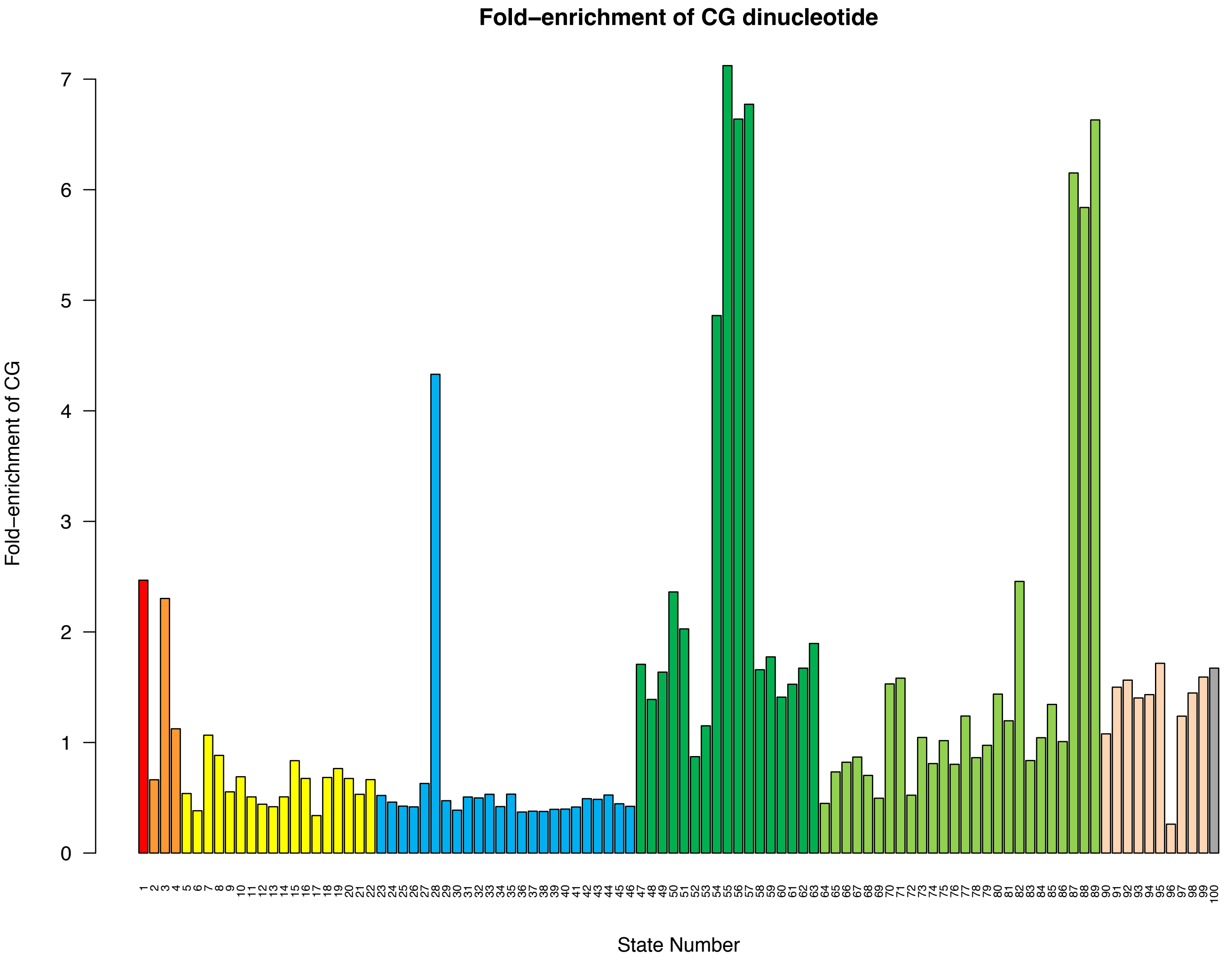

Supplementary Figure 12: Enrichment of CG dinucleotides in the states. The bar graph shows for each state its fold enrichment for CG dinucleotides. States 55-57 and 87-89 had the highest enrichments followed by states 28 and 54.

state	detection of chemical stimulus involved in sensory perception of smell	system development	nucleosome	cellular developmental process	regulation of cellular process	transcription from RNA polymerase II promoter	cell-cell signaling	calcium ion binding	receptor binding	antigen binding	protein-DNA complex assembly	cell adhesion	mesonephros development	neuromuscular process
1	0.3	14.1	41.3	5.4	4.2	7.9	7.9	12.2	0.0	0.0	13.2	8.8	1.7	2.5
2	0.0	41.2	0.1	30.6	6.9	19.0	11.0	3.6	7.5	0.0	0.0	3.7	5.3	1.7
3	319.6	2.6	0.2	0.1	18.6	0.1	2.8	13.5	0.0	0.0	0.0	4.3	0.8	0.1
4	0.0	21.0	0.0	17.7	5.6	24.7	8.1	0.1	6.5	0.0	0.1	2.2	2.2	4.0
5	0.0	26.8	0.0	18.9	10.0	13.4	20.8	0.5	11.3	0.1	0.0	8.6	3.6	3.4
6	0.0	5.6	0.0	5.3	3.7	2.4	3.4	1.5	1.1	0.0	0.0	2.7	2.2	0.1
7	0.2	21.6	0.1	12.7	11.6	9.2	12.0	1.4	5.1	0.0	0.0	1.5	3.6	9.4
8	0.0	19.8	0.0	21.0	2.9	8.2	9.0	1.1	6.5	0.7	0.0	11.7	5.3	2.9
9	0.0	10.2	0.0	6.4	3.6	1.7	4.2	4.4	4.1	0.0	0.0	4.6	4.1	0.4
10	0.0	8.6	0.0	6.2	1.0	0.7	2.9	3.3	5.1	0.0	0.0	7.5	2.6	0.7
11	0.1	0.6	0.0	0.1	0.1	0.0	0.5	4.4	1.0	0.5	0.0	0.1	0.0	0.0
12	1.7	0.3	0.1	0.2	0.6	0.0	0.8	0.7	1.2	0.1	0.0	0.4	0.0	0.1
13	0.0	0.7	0.0	0.6	0.0	0.0	0.7	0.5	0.1	0.1	0.0	0.7	0.0	0.1
14	0.0	2.0	0.0	2.6	2.9	0.4	5.2	1.4	2.6	0.3	0.0	0.2	0.4	0.0
15	0.0	15.4	0.0	10.8	2.9	2.0	4.6	2.5	6.0	0.0	0.0	5.2	3.1	0.3
16	0.1	0.0	0.0	0.0	0.0	0.0	0.0	0.5	0.1	1.4	0.0	0.4	0.0	0.1
17	0.3	0.0	0.0	0.0	0.0	0.0	0.0	0.3	0.0	0.7	0.0	0.2	0.0	0.0
18	0.0	0.1	0.0	0.0	0.0	0.0	0.2	0.1	0.1	1.0	0.0	0.5	0.0	0.1
19	0.0	19.8	0.0	15.5	2.6	1.1	8.1	3.1	9.5	0.1	0.0	10.5	3.1	0.9
20	0.0	5.1	0.1	2.1	2.0	1.4	1.1	0.4	0.1	0.1	0.3	0.7	0.1	1.7
21	1.2	0.4	0.1	0.1	0.0	0.0	0.1	0.5	0.1	0.0	0.4	1.4	0.0	0.1
22	0.0	0.1	0.2	0.1	0.1	0.1	0.9	0.5	0.7	0.0	0.3	1.0	0.1	0.1
23	0.0	0.0	0.1	0.0	0.0	0.0	0.0	0.0	0.0	0.5	0.3	0.0	0.0	0.1
24	0.0	0.0	2.3	0.0	0.0	0.0	0.0	0.0	0.0	0.1	3.2	0.1	0.0	0.0
25	0.0	0.0	0.0	0.0	0.0	0.0	0.0	0.0	0.0	0.1	0.3	0.0	0.0	0.1
26	0.0	0.0	0.0	0.0	0.0	0.0	0.0	0.0	0.0	0.5	0.7	0.1	0.0	0.0
27	0.0	0.0	0.4	0.0	0.0	0.0	0.0	0.0	0.0	0.1	0.9	0.0	0.0	0.3
28	60.0	5.8	0.9	5.9	24.9	12.5	10.5	0.9	1.3	1.4	0.1	0.9	1.1	0.7
29	0.5	0.0	0.1	0.0	0.0	0.0	0.0	0.0	0.0	0.5	0.1	0.0	0.0	0.7
30	0.2	0.0	3.9	0.0	0.0	0.0	0.0	0.0	0.0	0.5	2.0	0.0	0.0	0.3
31	1.5	0.0	1.5	0.0	0.0	0.0	0.0	0.0	0.0	0.0	1.4	0.0	0.0	0.1
32	0.0	0.0	2.3	0.0	0.3	0.0	0.0	0.0	0.0	1.0	1.7	0.0	0.0	0.0
33	15.3	0.0	2.8	0.0	1.0	0.1	0.0	0.0	0.0	1.7	1.4	0.0	0.6	0.4
34	16.5	0.0	1.5	0.0	0.0	0.0	0.0	0.0	0.0	0.1	1.2	0.1	0.0	0.1
35	0.0	0.0	0.2	0.0	0.0	0.1	0.1	0.4	0.0	0.0	1.2	0.0	0.0	0.0
36	0.0	0.0	0.1	0.0	0.0	0.0	0.0	0.0	0.0	0.1	0.3	0.0	0.0	0.1
37	0.0	0.0	0.1	0.0	0.0	0.0	0.0	0.0	0.0	0.1	0.1	0.0	0.0	0.4
38	0.1	0.0	0.9	0.0	0.1	0.1	0.0	0.0	0.0	1.0	4.0	0.1	0.0	0.0
39	4.8	0.0	10.7	0.0	0.0	0.0	0.0	0.1	0.0	0.5	3.6	0.5	0.0	0.0
40	0.9	0.0	0.1	0.0	0.0	0.0	0.0	0.0	0.0	0.3	1.4	0.3	0.0	0.3
41	0.9	0.0	0.2	0.0	0.0	0.0	0.0	0.5	0.0	0.0	0.1	0.2	0.0	0.0
42	0.5	0.0	0.0	0.0	0.0	0.0	0.0	0.4	0.0	0.0	0.0	0.0	0.0	0.1
43	0.0	0.0	5.0	0.0	0.1	0.0	0.0	0.0	0.0	0.1	2.5	0.0	0.0	0.0
44	28.0	0.0	3.9	0.0	0.1	0.0	0.0	0.0	0.0	1.7	0.9	0.0	0.0	0.0
45	6.7	0.0	4.4	0.0	0.1	0.0	0.0	0.1	0.3	1.7	2.9	0.0	0.0	0.0
46	2.6	0.0	0.1	0.0	0.0	0.0	0.0	0.4	0.1	0.3	0.2	0.1	0.0	0.0
47	0.0	0.1	0.1	0.1	0.0	0.0	0.0	0.6	1.3	0.7	0.0	1.0	0.1	0.3
48	3.1	0.0	2.8	0.0	0.0	0.0	0.0	0.0	0.0	1.0	1.2	0.0	0.0	0.4
49	0.0	0.0	0.2	0.0	0.0	0.0	0.1	0.0	0.0	0.5	0.3	0.0	0.0	0.4
50	0.0	29.6	0.0	21.7	5.6	4.7	12.6	2.9	15.8	0.1	0.0	11.1	8.0	0.4
51	0.0	0.5	0.0	0.0	0.1	0.0	0.2	0.7	0.2	0.5	0.0	0.8	0.0	0.1
52	0.0	0.7	0.0	0.2	0.4	0.0	0.6	0.9	0.8	0.0	0.0	0.8	0.0	0.1
53	0.0	44.6	0.0	30.2	7.9	9.2	18.8	2.1	15.4	0.3	0.0	9.4	9.5	2.0
54	53.2	3.9	32.3	0.9	1.7	0.9	2.8	15.9	0.2	0.0	8.9	8.6	1.7	1.2
55	0.0	22.2	0.0	18.3	2.6	4.0	12.0	5.0	13.3	0.1	0.0	6.3	11.0	2.5
56	6.3	0.1	0.1	0.0	0.0	0.0	0.2	0.5	0.3	0.3	0.0	0.6	0.0	0.1
57	3.1	0.0	1.2	0.0	0.0	0.0	0.0	0.0	0.0	1.4	1.4	0.0	0.0	0.3
58	5.9	0.0	1.2	0.0	0.0	0.0	0.0	0.5	0.0	0.5	0.6	0.0	0.0	0.1
59	17.7	0.0	7.7	0.0	0.2	0.0	0.0	0.0	0.0	0.3	0.9	0.0	0.0	0.0
60	3.7	0.0	0.1	0.0	0.0	0.0	0.0	0.2	0.0	0.3	0.1	0.1	0.0	0.1
61	2.6	0.0	0.9	0.0	0.0	0.0	0.0	0.0	0.1	0.5	0.7	0.0	0.0	0.7
62	0.0	0.0	0.1	0.0	0.0	0.0	0.0	0.0	0.1	1.0	0.9	0.0	0.0	0.1
63	0.0	1.4	0.0	0.2	0.2	0.0	0.2	1.2	0.6	0.5	0.1	2.6	0.1	0.1
64	1.7	0.0	0.1	0.0	0.0	0.0	0.0	0.7	0.2	1.4	0.4	0.0	0.1	0.1
65	0.3	0.0	6.3	0.0	0.0	0.0	0.0	0.1	0.0	0.3	3.6	0.0	0.0	0.0
66	0.1	0.9	0.0	0.4	0.0	0.0	0.1	2.1	0.3	0.3	0.0	0.7	0.0	0.1
67	0.0	0.0	0.0	0.0	0.0	0.0	0.2	0.5	0.2	0.5	0.0	0.3	0.0	0.1
68	0.0	0.0	0.0	0.0	0.0	0.0	0.0	0.7	0.0	0.5	0.7	0.0	0.0	0.1
69	0.5	0.0	0.6	0.0	0.0	0.0	0.0	0.0	0.0	1.0	0.9	0.0	0.0	0.3
70	0.5	0.0	4.4	0.0	0.0	0.0	0.0	0.0	0.0	2.3	1.4	0.0	0.0	0.0
71	0.1	0.0	0.2	0.0	0.0	0.0	0.0	0.0	0.0	8.2	0.2	0.0	0.0	0.1
72	0.1	0.0	0.6	0.0	0.0	0.0	0.0	0.0	0.0	7.4	0.3	0.0	0.0	0.0
73	1.0	0.0	0.4	0.0	0.0	0.0	0.0	0.0	0.1	4.6	0.3	0.0	0.0	0.0
74	0.0	0.0	0.4	0.0	0.0	0.0	0.0	0.0	0.0	0.3	0.7	0.0	0.0	0.1
75	0.0	0.0	0.4	0.0	0.0	0.0	0.0	0.0	0.0	1.4	0.3	0.0	0.0	0.0
76	0.0	0.0	0.4	0.0	0.0	0.0	0.0	0.0	0.0	0.7	0.0	0.0	0.0	0.0
77	0.0	0.0	0.2	0.0	0.0	0.1	0.0	0.0	0.0	0.3	0.7	0.1	0.0	0.0
78	0.1	0.0	0.4	0.0	0.0	0.0	0.0	0.0	0.1	0.3	0.2	0.0	0.0	0.0
79	0.0	0.0	0.4	0.0	0.2	0.0	0.0	0.0	0.0	0.5	0.3	0.0	0.0	0.1
80	0.0	0.0	0.9	0.0	0.0	0.0	0.0	0.0	0.1	0.3	2.2	0.5	0.0	0.0
81	2.8	0.0	1.2	0.0	0.0	0.0	0.0	0.0	0.0	0.7	0.6	0.0	0.2	0.0
82	58.0	0.0	7.7	0.0	0.6	0.0	0.0	0.0	0.0	0.3	3.2	0.0	0.0	0.1
83	0.0	0.0	0.0	0.0	0.0	0.0	0.0	0.0	0.0	1.0	0.0	0.0	0.0	0.0
84	0.1	0.0	0.1	0.0	0.0	0.0	0.0	0.0	0.0	0.5	0.1	0.0	0.0	0.0
85	0.0	0.0	0.4	0.0	0.0	0.0	0.0	0.1	0.0	0.1	0.3	0.0	0.0	0.0
86	0.0	0.0	0.0	0.0	0.0	0.0	0.0	0.1	0.0	1.0	0.2	0.0	0.0	0.0
87	0.0	0.0	0.9	0.0	0.0	0.0	0.0	0.0	0.0	0.1	0.3	0.0	0.0	0.1
88	0.0	0.0	0.6	0.0	0.0	0.0	0.0	0.0	0.0	2.8	0.0	0.0	0.0	0.0
89	3.4	0.0	0.2	0.0	0.0	0.0	0.0	0.0	0.1	14.5	0.0	0.0	0.0	0.1
90	0.1	0.0	0.2	0.0	0.0	0.0	0.0	0.0	0.0	0.5	0.3	0.0	0.0	0.0
91	9.4	0.0	1.2	0.0	0.0	0.0	0.0	0.0	0.0	1.0	0.7	0.0	0.0	0.1
92	0.0	0.0	0.0	0.0	0.0	0.0	0.0	0.0	0.0	0.5	0.4	0.0	0.0	0.0
93	1.0	0.0	0.0	0.0	0.0	0.0	0.0	0.0	0.1	2.8	0.0	0.0	0.0	0.0
94	0.1	0.0	0.1	0.0	0.0	0.0	0.0	0.2	0.1	1.0	0.2	0.0	0.1	0.1
95	17.1	0.0	0.1	0.0	0.0	0.0	0.0	0.0	0.0	1.0	0.9	0.0	0.0	0.1
96	0.1	0.0	0.1	0.0	0.0	0.0	0.0	0.0	0.2	0.3	0.3	0.1	0.0	0.0
97	0.0	0.0	0.1	0.0	0.0	0.0	0.0	0.0	0.0	0.7	0.3	0.0	0.1	0.4
98	0.3	0.0	0.1	0.0	0.0	0.0	0.0	0.0	0.0	0.5	0.3	0.0	0.0	0.1
99	0.0	0.0	0.0	0.0	0.0	0.0	0.0	0.0	0.0	0.7	0.1	0.0	0.0	0.1
100	9.7	0.1	26.1	0.1	0.3	1.1	0.1	0.1	0.1	0.7	14.0	0.0	0.3	0.2

Supplementary Figure 13: GO term enrichment p-values. This figure is an extended version of **Fig. 4b**, now showing $-\log_{10}$ p-values for GO term enrichments for all states. The criteria for including a GO term were the same as in **Fig. 4b**, except the top 10 term criterion was relaxed to include four additional terms. Values that are boxed mark the top most significant enrichment for the term of the column.

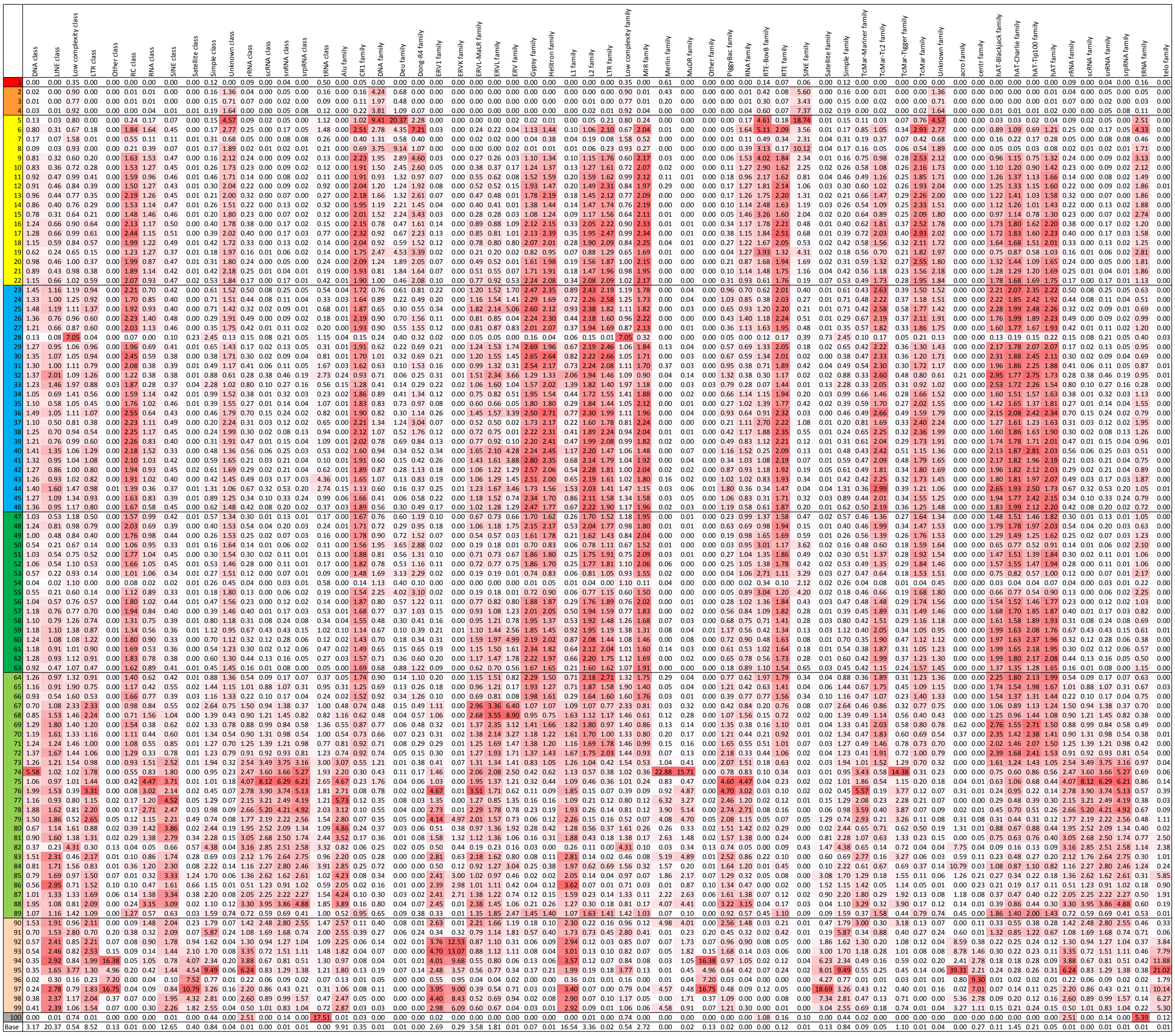

Supplementary Figure 14: Conservation state enrichments for RepeatMasker classes and families of repeats. The rows correspond to different conservation states and columns correspond to different repeat classes or families. The first 16 columns are repeat classes, and the remaining are repeat families. The values correspond to fold enrichment for the repeat class or family for the conservation state. Values are shaded in a column specific manner. The last row gives the % of the genome the repeat class or family covers.

state	1_TssA	2_PromU	3_PromD1	4_PromD2	5_Tx5	6_Tx	7_Tx3	8_TxWk	9_TxReg	10_TxEnh5	11_TxEnh3	12_TxEnhW	13_EnhA1	14_EnhA2	15_EnhAF	16_EnhW1	17_EnhW2	18_EnhAc	19_DNase	20_ZNF/Rpts	21_Het	22_PromP	23_PromBiv	24_ReprPC	25_Quies
1	2.1	3.4	5.4	3.8	0.3	8.6	8.7	1.6	6.7	1.3	8.5	0.8	1.4	1.6	1.0	1.2	1.0	0.5	1.0	4.0	0.3	1.6	6.3	1.6	0.5
2	1.0	2.5	1.1	1.7	0.3	2.0	2.8	1.2	2.7	1.6	4.2	1.1	3.5	4.7	3.1	3.3	3.4	1.2	1.9	0.4	0.1	0.7	3.4	1.6	0.8
3	1.3	2.7	2.3	2.5	0.3	4.2	4.9	1.4	3.7	1.5	5.8	1.0	2.6	3.1	2.2	2.4	2.3	1.0	1.5	3.2	0.3	1.2	4.1	1.6	0.7
4	4.7	4.0	5.7	3.0	0.4	2.8	3.0	1.1	4.7	2.0	3.7	1.3	2.5	3.6	2.3	3.0	2.4	1.1	2.1	0.8	0.2	1.4	4.8	1.7	0.8
5	3.6	3.5	3.8	2.7	0.6	1.1	1.3	1.0	3.9	2.3	2.6	1.7	3.3	4.2	2.9	3.1	2.9	1.6	2.5	0.2	0.1	1.0	3.8	2.0	0.9
6	1.4	2.2	2.2	2.7	1.0	0.9	1.0	1.0	2.3	2.1	1.6	2.1	2.0	2.3	2.0	2.1	2.2	1.2	2.0	0.2	0.1	0.8	2.1	1.9	1.0
7	5.4	3.9	5.9	2.3	0.5	2.9	4.5	1.8	3.6	1.7	4.6	1.0	2.1	2.0	1.7	1.8	1.7	1.0	1.2	1.3	0.2	1.5	5.6	1.6	0.7
8	2.6	2.6	2.9	2.2	0.6	1.4	1.9	1.1	3.1	2.0	2.9	1.5	2.7	3.2	2.5	2.4	2.5	1.3	2.0	0.4	0.1	0.9	3.0	1.7	0.9
9	1.3	2.0	2.1	2.4	1.0	0.9	1.0	1.0	2.1	2.0	1.6	2.0	1.8	2.1	2.0	2.0	2.1	1.1	1.9	0.2	0.1	0.7	2.1	1.9	1.0
10	1.4	2.1	2.0	2.1	1.0	1.1	1.3	1.1	2.1	2.0	1.8	1.9	1.9	2.2	1.9	1.9	2.0	1.1	1.8	0.3	0.2	0.7	2.0	1.7	1.0
11	1.1	1.6	1.4	1.6	1.0	1.0	1.3	1.1	1.4	1.6	1.4	1.6	1.5	1.5	1.4	1.6	1.6	1.0	1.5	0.4	0.2	0.6	1.4	1.5	1.0
12	0.8	1.2	1.1	1.3	0.9	0.7	0.8	0.9	1.1	1.2	1.0	1.3	1.3	1.4	1.3	1.3	1.5	0.9	1.4	0.3	0.3	0.6	1.3	1.3	1.0
13	0.9	1.4	1.4	1.3	1.0	0.8	0.9	1.0	1.0	1.3	1.0	1.5	1.2	1.4	1.4	1.3	1.6	0.9	1.4	0.3	0.2	0.6	1.2	1.3	1.0
14	1.7	3.2	2.7	2.3	1.3	1.4	1.5	1.2	2.0	2.4	1.9	2.2	2.4	2.3	1.9	2.4	1.9	1.3	2.0	0.3	0.2	0.8	2.5	1.7	0.9
15	1.6	2.5	2.5	2.5	1.0	1.1	1.2	1.0	2.5	2.2	1.8	2.0	2.0	2.4	2.0	2.2	2.2	1.1	2.0	0.3	0.1	0.8	2.5	2.0	0.9
16	0.8	1.3	1.1	1.3	1.1	1.0	0.9	1.0	1.3	1.6	1.1	1.7	1.2	1.2	1.3	1.3	1.4	0.8	1.4	0.3	0.3	0.7	1.1	1.3	1.0
17	0.7	1.2	0.9	1.4	1.2	1.0	0.9	1.0	1.1	1.5	1.0	1.7	1.1	1.1	1.2	1.2	1.4	0.8	1.3	0.4	0.3	0.7	0.9	1.2	1.0
18	0.9	1.4	1.2	1.3	1.1	1.0	1.0	1.0	1.3	1.7	1.2	1.7	1.3	1.4	1.4	1.4	1.5	0.9	1.5	0.4	0.3	0.7	1.2	1.3	1.0
19	1.8	2.4	2.4	2.4	0.9	1.1	1.3	1.0	2.5	2.2	2.1	2.0	2.2	2.7	2.2	2.2	2.3	1.2	2.1	0.3	0.1	0.8	2.6	1.9	0.9
20	3.7	1.9	3.1	1.4	0.9	0.9	1.1	1.0	1.4	1.4	1.3	1.5	1.4	1.5	1.5	1.4	1.6	0.8	1.4	0.3	0.2	0.9	2.2	1.3	1.0
21	1.0	1.4	1.4	1.4	0.9	1.0	1.2	1.0	1.5	1.5	1.6	1.5	1.5	1.7	1.5	1.5	1.6	0.8	1.4	0.4	0.3	0.6	1.4	1.4	1.0
22	2.5	1.9	2.2	1.1	1.2	1.2	1.2	1.1	1.0	1.7	1.4	1.5	1.2	1.3	1.3	1.5	1.3	0.8	1.4	0.4	0.3	1.0	1.8	1.2	1.0
23	0.8	1.1	0.7	0.8	1.6	1.6	1.2	1.2	1.1	1.4	1.3	1.4	1.0	0.8	1.0	1.1	0.9	0.8	1.1	1.0	0.5	0.7	0.7	0.9	1.0
24	0.5	0.7	0.5	0.7	1.2	1.2	1.0	1.0	0.9	1.3	1.0	1.2	0.9	0.8	0.9	0.9	0.9	0.5	1.1	0.7	0.6	0.6	0.5	0.9	1.0
25	0.5	0.6	0.4	0.6	1.1	1.0	0.9	0.9	0.7	1.0	0.7	1.1	0.7	0.9	0.9	0.9	1.0	0.6	1.0	0.7	0.5	0.6	0.5	0.9	1.1
26	0.7	0.8	0.8	0.9	1.3	1.2	1.2	1.1	0.9	1.2	1.2	1.4	0.9	0.9	1.0	1.0	1.1	0.7	1.1	0.7	0.4	0.6	0.6	0.9	1.0
27	0.9	1.3	1.1	1.3	1.4	1.5	1.3	1.2	1.5	1.8	1.4	1.9	1.2	1.3	1.3	1.4	1.4	0.8	1.4	0.7	0.3	0.7	0.9	1.2	1.0
28	35.1	4.5	19.9	2.4	0.5	2.2	2.4	1.0	3.6	1.3	2.0	0.6	1.3	1.3	0.8	1.3	0.7	0.9	1.1	5.4	1.4	8.0	13.1	1.9	0.6
29	0.6	0.5	0.5	0.5	0.9	0.6	0.7	0.8	0.6	0.8	0.6	1.0	0.7	0.8	1.0	0.8	1.1	0.5	1.0	0.5	0.5	0.5	0.5	0.9	1.1
30	0.3	0.4	0.2	0.4	1.1	0.8	0.7	0.9	0.4	0.7	0.5	0.9	0.7	0.6	0.7	0.7	0.9	0.6	0.9	0.7	0.6	0.4	0.3	0.7	1.1
31	0.8	0.8	0.6	0.5	1.2	1.0	0.9	1.1	0.4	1.0	0.7	1.1	0.9	0.7	0.8	0.9	0.9	0.8	1.1	0.9	0.7	0.7	0.6	0.9	1.0
32	0.5	0.5	0.3	0.4	1.3	1.0	0.7	1.1	0.5	0.9	0.6	0.8	0.6	0.6	0.6	0.7	0.6	0.6	0.8	1.5	1.1	0.7	0.5	0.9	1.0
33	1.1	0.4	0.4	0.4	1.0	0.8	0.7	1.0	0.3	0.4	0.5	0.6	0.5	0.4	0.5	0.6	0.6	0.4	0.7	1.1	1.5	1.2	0.5	0.8	1.0
34	0.7	0.8	0.6	1.1	1.3	1.0	1.0	1.1	0.9	1.0	1.0	1.2	0.6	0.6	0.8	1.0	0.9	0.5	1.0	0.6	0.7	0.7	0.7	1.0	1.0
35	1.3	2.1	1.7	1.7	1.5	1.8	1.6	1.4	1.9	2.1	1.7	2.0	1.7	1.6	1.5	1.7	1.4	1.1	1.6	0.6	0.3	0.8	1.4	1.4	0.9
36	0.4	0.7	0.4	0.8	1.5	1.3	1.0	1.1	0.9	1.3	0.9	1.4	0.8	0.8	0.9	1.0	1.0	0.7	1.1	0.7	0.5	0.7	0.4	0.9	1.0
37	0.8	1.2	1.1	1.6	1.4	1.4	1.3	1.2	1.5	1.7	1.4	1.9	1.3	1.3	1.4	1.4	1.5	0.8	1.4	0.5	0.2	0.6	0.9	1.2	1.0
38	0.6	0.8	0.7	0.9	1.4	1.2	1.1	1.2	0.8	1.2	0.9	1.6	0.8	0.8	1.0	1.0	1.2	0.7	1.1	0.8	0.3	0.5	0.5	0.9	1.0
39	0.4	0.7	0.5	0.7	1.1	0.8	0.8	1.0	0.5	0.8	0.7	1.2	0.8	0.7	0.9	0.8	1.1	0.6	1.0	0.8	0.6	0.6	0.5	0.9	1.0
40	0.3	0.4	0.2	0.4	1.2	0.9	0.7	0.9	0.4	0.8	0.5	1.1	0.6	0.5	0.7	0.7	0.9	0.5	0.9	0.8	0.6	0.4	0.2	0.8	1.1
41	0.4	0.6	0.4	0.7	1.0	0.7	0.6	0.9	0.5	1.0	0.6	1.1	0.7	0.7	0.9	0.9	1.1	0.6	1.1	0.4	0.4	0.5	0.5	1.1	1.1
42	0.8	1.4	0.8	1.0	1.4	1.1	1.0	1.1	0.9	1.4	1.0	1.5	1.1	1.1	1.1	1.4	1.2	1.0	1.4	0.5	0.5	0.7	1.0	1.3	1.0
43	0.7	0.9	0.6	0.7	1.5	1.2	1.0	1.2	0.6	1.3	1.1	1.4	0.9	0.9	1.0	1.1	1.0	0.9	1.2	1.0	0.5	0.7	0.6	1.0	1.0
44	0.8	0.7	0.5	0.6	1.5	1.2	0.9	1.1	0.6	0.7	0.6	0.9	0.7	0.6	0.7	0.8	0.6	0.7	1.0	1.5	1.2	1.1	0.7	1.1	1.0
45	0.5	0.7	0.4	0.6	1.1	0.9	0.8	1.0	0.5	0.8	0.7	0.9	0.6	0.7	0.8	0.8	0.9	0.6	1.0	0.7	0.6	0.5	0.5	1.0	1.1
46	0.5	0.8	0.5	0.9	1.3	1.2	1.2	1.2	0.8	1.0	1.1	1.3	0.9	0.7	0.9	1.0	1.0	0.9	1.1	0.6	0.5	0.6	0.5	1.0	1.0
47	1.4	1.8	1.6	1.4	1.2	1.4	1.3	1.1	1.9	2.1	1.6	1.9	1.6	1.7	1.5	1.7	1.6	1.0	1.7	0.5	0.3	0.8	1.6	1.4	1.0
48	0.6	0.9	0.6	0.9	1.3	1.2	0.9	1.0	1.0	1.3	1.0	1.5	0.9	0.9	1.0	1.0	1.1	0.7	1.2	0.8	0.5	0.7	0.6	1.0	1.0
49	1.3	1.9	1.7	1.6	1.3	1.6	1.5	1.2	2.0	2.1	1.7	2.0	1.6	1.6	1.5	1.7	1.6	0.9	1.6	0.6	0.2	0.7	1.4	1.4	0.9
50	2.2	2.7	2.9	2.5	0.8	1.1	1.4	1.0	2.7	2.1	2.1	1.8	2.2	2.6	2.1	2.2	2.2	1.2	2.0	0.3	0.1	0.9	3.0	2.0	0.9
51	1.1	1.7	1.4	1.4	1.1	1.0	1.0	1.0	1.4	1.7	1.2	1.7	1.4	1.5	1.4	1.5	1.5	0.9	1.5	0.4	0.3	0.7	1.5	1.4	1.0
52	1.2	1.8	1.5	1.5	1.1	1.1	1.1	1.0	1.6	1.9	1.3	1.8	1.5	1.6	1.5	1.6	1.6	1.0	1.6	0.4	0.3	0.8	1.6	1.4	1.0
53	2.3	2.9	3.0	2.5	0.8	1.3	1.6	1.1	2.9	2.3	2.4	1.9	2.3	2.8	2.2	2.4	2.3	1.2	2.1	0.4	0.1	0.9	3.2	2.0	0.9
54	2.5	3.0	3.3	2.2	0.3	3.8	4.8	1.4	3.7	1.5	5.2	0.9	2.3	2.6	1.8	2.2	1.9	1.0	1.5	2.8	0.4	1.3	4.4	1.6	0.7
55	2.4	2.5	2.8	2.3	0.8	1.2	1.4	1.0	2.5	2.0	2.1	1.8	2.1	2.5	2.1	2.2	2.2	1.1	2.0	0.3	0.1	0.8	2.8	1.9	0.9
56	1.4	1.4	1.4	1.2	1.0	0.9	0.9	0.9	1.2	1.5	1.1	1.5	1.2	1.3	1.3	1.4	1.4	0.8	1.4	0.4	0.4	0.7	1.3	1.3	1.0
57	1.1	1.2	1.0	1.0	1.3	1.3	1.1	1.1	1.2	1.5	1.1	1.5	1.1	1.0	1.1	1.2	1.2	0.8	1.3	0.7	0.5	0.7	0.9	1.1	1.0
58	1.0	1.2	1.0	0.9	1.1	1.1	1.1	1.0	1.2	1.3	1.2	1.3	1.1	1.1	1.1	1.2	1.1	0.8	1.3	0.6	0.5	0.7	1.0	1.2	1.0
59	1.3	1.3	1.0	0.8	1.4	1.4	1.0	1.1	1.0	1.4	1.1	1.3	1.0	1.0	0.9	1.2	0.9	0.9	1.3	1.3	0.9	1.1	1.1	1.2	1.0
60	0.5	0.7	0.4	0.5	1.1	0.9	0.7	0.9	0.6	1.1	0.6	1.2	0.8	0.8	0.9	0.9	1.0	0.6	1.1	0.7	0.6	0.5	0.5	1.1	1.1
61	0.7	0.7	0.5	0.5	1.0	0.9	0.8	0.9	0.6	1.0	0.7	1.1	0.9	0.9	0.9	0.9	1.0	0.6	1.1	0.6	0.6	0.5	0.6	0.9	1.1
62	0.9	1.0	0.8	0.8	1.3	1.5	1.2	1.0	1.2	1.6	1.2	1.4	1.0	1.0	1.0	1.1	1.0	0.7	1.2	0.8	0.5	0.7	0.7	1.0	1.0
63	3.2	2.0	2.8	1.3	0.9	1.1	1.3	1.0	1.6	1.7	1.6	1.5	1.5	1.7	1.5	1.6	1.6	0.8	1.5	0.4	0.3	0.9	2.4	1.4	1.0
64	0.8	0.6	0.5	0.6	1.0	0.8	0.9	0.9	0.5	0.9	0.7	0.9	0.8	0.7	0.9	0.8	1.0	0.7	1.0	0.6	0.5	0.6	0.5	0.9	1.1
65	0.9	0.9	0.7	0.7	1.2	1.1	1.0	1.0	0.8	1.2	0.9	1.2	0.8	0.8	0.9	1.0	0.9	0.8	1.0	0.7	0.6	0.7	0.7	1.0	1.0
66	1.4	1.5	1.3	1.4	1.0	1.1	1.2	1.1	1.5	1.6	1.3	1.5	1.3	1.4	1.3	1.4	1.4	0.7	1.4	0.4	0.3	0.7	1.4	1.4	1.0
67	1.3	1.1	1.0	1.0	1.0	1.0	1.1	1.0	1.1	1.2	1.1	1.1	1.1	1.2	1.1	1.0	1.1	0.9	1.1	0.5	0.5	0.9	1.2	1.2	1.0
68	0.4	0.4	0.3	0.6	1.2	1.0	0.9	1.0	0.6	0.8	0.6	0.9	0.6	0.6	0.7	0.6	0.8	0.7	0.7	0.8	0.7	0.6	0.3	0.8	1.1
69	0.4	0.4	0.2	0.4	1.1	0.9	0.7	1.0	0.4	0.6	0.5	0.7	0.6	0.4	0.6	0.6	0.7	0.7	0.8	1.0	1.1	0.7	0.3	0.9	1.1
70	0.8	0.7	0.5	0.5	1.2	1.1	0.8	1.0	0.6	1.0	0.7	0.9	0.7	0.6	0.7	0.9	0.7	0.7	1.0	1.2	1.1	1.0	0.7	1.0	1.0
71	1.1	0.8	0.8	0.7	1.3	1.3	1.1	1.1	0.8	1.2	0.8	1.0	0.8	0.7	0.8	0.9	0.8	0.9	1.0	1.0	0.8	0.9	0.7	1.0	1.0
72	0.6	0.6	0.4	0.6	1.3	1.1	0.9	1.1	0.5	0.8	0.6	0.8	0.6	0.5	0.6	0.7	0.6	0.8	0.8	1.2	1.0	0.8	0.4	0.9	1.0
73	0.6	0.5	0.3	0.8	1.6	1.2	1.1	1.4	0.4	0.7	0.6	0.9	0.6	0.4	0.7	0.6	0.6	1.2	0.7	1.2	1.0	0.9	0.5	1.2	1.0
74	0.4	0.6	0.5	0.9	1.6	1.4	1.4	1.3	0.8	0.9	0.8	1.2	0.8	0.7	0.9	0.7	0.9	0.9	0.8	1.1	0.6	0.7	0.5	1.0	1.0
75	0.3	0.4	0.2	0.9	1.6	1.2	1.3	1.5	0.5	0.8	0.6	0.9	0.6	0.4	0.7	0.6	0.7	1.2	0.6	1.0	0.6	0.8	0.3	1.0	1.0
76	0.1	0.3	0.1	0.4	1.1	0.8	0.7	1.0	0.4	0.5	0.5	0.6	1.0	0.7	1.0	0.7	0.8	1.5	0.7	1.8	2.0	0.6	0.1	0.6	1.0
77	0.5	0.4	0.3	0.8	2.1	1.2	1.4	2.0	0.2	0.4	0.5	0.6	0.6	0.3	0.6	0.4	0.4	1.6	0.4	1.1	1.0	1.2	0.2	0.8	0.9
78	0.4	0.5	0.3	0.7	1.4	1.1	0.9	1.2	0.4	0.4	0.7	0.6	0.8	0.5	0.8	0.7	0.6	1.3	0.7	2.1	1.8	1.0	0.3	0.7	1.0
79	0.2	0.3	0.1	0.4	1.1	0.8	0.6	1.0	0.4	0.4	0.5	0.4	0.9	0.5	0.8	0.7	0.5	1.6	0.7	2.7	2.8	0.9	0.2	0.7	1.0
80	0.5	0.4	0.2	0.8	1.9	1.2	1.3	1.7	0.5	0.6	0.4	0.7	0.5	0.3	0.5	0.4	0.5	1.5	0.5	1.0	0.7	1.0	0.2	0.8	1.0
81	0.5	0.5	0.2	0.4	1.3	1.0	0.8	1.3	0.4	0.4	0.5	0.4	0.4	0.3	0.6	0.5	0.4	1.3	0.6	2.3	2.0	1.2	0.5	1.0	1.0
82	4.2	1.2	1.7	0.9	0.9	1.6	1.6	1.3	0.9	0.7	1.0	0.5	0.4	0.6	0.5	0.8	0.4	1.1	1.1	6.4	3.5	4.8	2.1	2.1	0.8
83	0.2	0.1	0.1	0.4	0.7	0.4	0.4	0.8	0.1	0.2	0.2	0.2	0.4	0.3	0.4	0.2	0.3	1.1	0.4	2.0	2.5	0.6	0.1	0.4	1.1
84	0.4	0.2	0.1	0.6	1.1	0.7	0.7	1.1	0.1	0.2	0.2	0.3	0.3	0.2	0.3	0.2	0.2	0.7	0.3	1.0	1.6	1.1	0.3	0.8	1.0
85	0.2	0.1	0.1	0.3	1.3	0.7	0.6	1.4	0.1	0.2	0.2	0.2	0.4	0.2	0.3	0.2	0.2	1.1	0.3	1.6	2.1	1.0	0.1	0.5	1.0
86	0.1	0.2	0.0	0.2	0.7	0.4	0.3	0.9	0.1	0.1	0.1	0.1	0.3	0.2	0.4	0.3	0.2	1.0	0.3	1.9	2.5	0.7	0.1	0.4	1.1
87	0.4	0.4	0.2	0.6	1.5	1.0	0.9	1.3	0.4	0.4	0.5	0.5	0.6	0.4	0.6	0.6	0.4	1.5	0.6	2.0	2.0	1.1	0.3	0.8	1.0
88	0.3	0.4	0.3	0.7	1.5	1.1	1.1	1.3	0.5	0.7	0.6	0.8	0.8	0.6	0.8	0.6	0.7	1.3	0.7	1.3	1.3	0.7	0.3	0.8	1.0
89	1.4	0.8	0.9	0.7	1.1	1.1	1.0	1.0	0.8	1.1	0.8	1.0	0.8	0.8	0.8	0.9	0.8	0.8	1.0	0.9	0.9	1.0	0.8	1.1	1.0
90	0.2	0.3	0.1	0.4	1.1	0.7	0.6	1.0	0.2	0.3	0.4	0.4	0.6	0.3	0.6	0.5	0.5	0.9	0.5	1.8	2.6	0.7	0.2	0.7	1.0
91	0.8	0.3	0.2	0.5	1.3	0.8	0.8	1.3	0.2	0.4	0.3	0.5	0.4	0.3	0.4	0.5	0.4	1.1	0.6	1.0	1.4	1.5	0.4	1.0	1.0
92	0.2	0.2	0.1	0.3	0.7	0.4	0.4	0.9	0.3	0.2	0.3	0.2	0.7	0.4	0.6	0.4	0.4	3.0	0.5	1.9	3.1	1.2	0.2	0.5	1.1
93	0.2	0.3	0.1	0.1	0.5	0.2	0.3	0.7	0.1	0.2	0.2	0.1	0.7	0.3	0.6	0.4	0.4	2.5	0.5	2.0	3.9	1.4	0.3	0.8	1.1
94	0.7	0.2	0.1	0.3	0.3	0.2	0.2	0.7	0.2	0.1	0.3	0.1	0.7	0.2	0.5	0.3	0.3	3.0	0.5	1.1	3.3	5.2	0.1	0.4	1.1
95	0.8	0.3	0.2	0.5	0.5	0.3	0.4	0.9	0.1	0.1	0.2	0.2	0.4	0.2	0.3	0.3	0.2	1.7	0.5	1.1	3.0	2.9	0.6	0.9	1.0
96	0.0	0.0	0.0	0.2	0.0	0.0	0.0	0.1	0.0	0.0	0.0	0.0	0.0	0.0	0.0	0.0	0.0	0.2	0.0	0.3	1.1	1.3	0.0	0.0	1.1
97	0.3	0.1	0.1	0.7	0.2	0.1	0.1	0.5	0.1	0.1	0.1	0.1	0.3	0.1	0.2	0.2	0.1	1.2	0.2	0.7	2.2	4.1	0.0	0.2	0.9
98	0.3	0.2	0.1	0.5	0.7	0.3	0.3	0.8	0.0	0.1	0.2	0.1	0.9	0.2	0.5	0.3	0.2	1.9	0.4	1.4	2.9	2.2	0.1	0.6	1.1
99	0.1	0.1	0.0	0.2	0.8	0.3	0.4	1.0	0.1	0.1	0.1	0.1	0.4	0.1	0.3	0.2	0.2	1.2	0.3	1.5	2.2	1.1	0.1	0.4	1.1
100	0.4	0.4	0.2	0.2	0.7	1.5	0.9	0.7	0.1	0.3	0.4	0.5	0.3	0.7	0.6	0.5	0.4	0.5	1.4	8.4	2.8	1.9	1.2	1.5	1.0
Base	0.23	0.28	0.38	0.23	1.89	0.63	3.84	5.49	0.07	0.12	0.09	0.45	0.13	0.08	0.46	0.25	2.06	0.04	0.44	0.24	2.59	0.13	0.42	1.35	76.19

Supplementary Figure 15: Conservation state enrichments for chromatin states. This is an extended version of **Fig. 4a**. Each row corresponds to one of the 100 conservation states. Each column corresponds to one of 25 chromatin states from the Roadmap Epigenomics imputation based chromatin state model defined across 127 samples of diverse cell and tissue types[3]. The values reported are the median fold enrichment across the 127 samples of the chromatin state for the conservation state. Values that are boxed were the maximum value of the column.

a

	GERP++ elements	PhastCons elements	SiPhy-Omega elements	SiPhy-Pi elements
1	14.32	17.94	21.98	16.49
2	12.62	14.19	16.40	13.18
3	11.98	9.39	14.71	12.60
4	10.99	10.45	13.54	11.55
5	9.84	9.08	11.27	9.77
6	3.37	2.92	3.22	3.48
7	3.77	2.09	3.10	3.56
8	5.77	2.75	4.02	4.48
9	2.09	1.01	1.44	1.99
10	1.65	0.65	0.99	1.62
11	0.85	0.64	0.54	0.84
12	1.09	0.84	0.70	1.19
13	1.35	1.05	0.99	1.29
14	1.17	0.94	0.88	1.21
15	1.61	0.61	0.96	1.64
16	0.34	0.19	0.16	0.35
17	0.65	0.77	0.49	0.64
18	0.41	0.18	0.18	0.47
19	2.86	1.22	1.94	2.70
20	1.34	1.00	1.00	1.29
21	1.02	0.72	0.65	0.88
22	0.33	0.40	0.24	0.35
23	0.09	0.37	0.09	0.07
24	0.09	0.28	0.08	0.07
25	0.11	0.31	0.11	0.11
26	0.31	0.44	0.22	0.26
27	0.34	0.20	0.15	0.30
28	3.00	3.27	2.27	1.91
29	0.16	0.28	0.12	0.20
30	0.12	0.29	0.09	0.11
31	0.09	0.31	0.07	0.10
32	0.03	0.36	0.03	0.05
33	0.07	0.41	0.07	0.06
34	0.47	0.65	0.37	0.44
35	0.51	0.64	0.35	0.39
36	0.17	0.47	0.16	0.11
37	0.91	0.89	0.65	0.77
38	0.45	0.56	0.30	0.32
39	0.33	0.45	0.18	0.34
40	0.09	0.33	0.08	0.10
41	0.24	0.38	0.17	0.27
42	0.17	0.36	0.15	0.23
43	0.12	0.39	0.10	0.14
44	0.05	0.46	0.05	0.05
45	0.08	0.30	0.08	0.15
46	0.13	0.38	0.13	0.12
47	0.31	0.05	0.08	0.51
48	0.16	0.14	0.06	0.15
49	0.48	0.23	0.18	0.39
50	2.85	1.22	1.86	2.58
51	0.45	0.21	0.19	0.47
52	0.35	0.14	0.13	0.42
53	2.38	0.75	1.36	2.25
54	7.67	4.24	7.31	7.77
55	3.09	1.44	2.17	2.87
56	0.45	0.22	0.20	0.46
57	0.23	0.17	0.09	0.20
58	0.14	0.10	0.05	0.19
59	0.05	0.11	0.02	0.07
60	0.06	0.08	0.02	0.12
61	0.07	0.07	0.02	0.11
62	0.08	0.10	0.03	0.08
63	0.56	0.19	0.23	0.54
64	0.16	0.30	0.12	0.14
65	0.06	0.16	0.03	0.09
66	0.36	0.29	0.21	0.33
67	0.16	0.16	0.08	0.17
68	0.02	0.11	0.02	0.06
69	0.02	0.26	0.02	0.05
70	0.01	0.09	0.00	0.04
71	0.03	0.08	0.01	0.05
72	0.03	0.30	0.02	0.05
73	0.01	0.18	0.01	0.07
74	0.07	0.12	0.03	0.08
75	0.02	0.19	0.01	0.05
76	0.01	0.14	0.01	0.02
77	0.01	0.11	0.00	0.03
78	0.00	0.14	0.00	0.03
79	0.01	0.11	0.00	0.02
80	0.01	0.16	0.01	0.06
81	0.01	0.39	0.01	0.03
82	0.33	3.57	0.16	0.08
83	0.00	0.09	0.00	0.01
84	0.00	0.26	0.00	0.03
85	0.00	0.06	0.00	0.01
86	0.00	0.08	0.00	0.01
87	0.01	0.06	0.00	0.03
88	0.02	0.06	0.01	0.04
89	0.07	0.09	0.02	0.09
90	0.01	0.13	0.01	0.02
91	0.02	0.24	0.01	0.07
92	0.01	0.07	0.01	0.02
93	0.01	0.04	0.00	0.01
94	0.01	0.05	0.01	0.01
95	0.04	1.52	0.02	0.03
96	0.00	0.00	0.00	0.00
97	0.00	0.03	0.00	0.00
98	0.00	0.02	0.00	0.00
99	0.00	0.04	0.00	0.01
100	1.96	15.51	0.99	0.31
Base	6.775	5.174	4.042	5.565

b

	mean PhyloP score	mean PhastCons core	mean GERP++ score
1	5.173	0.926	4.191
2	2.308	0.738	3.030
3	0.617	0.484	1.211
4	1.357	0.549	2.194
5	1.173	0.488	2.371
6	0.636	0.180	1.130
7	0.292	0.126	0.339
8	0.132	0.155	0.074
9	-0.029	0.065	-0.186
10	-0.243	0.042	-1.062
11	0.112	0.051	-0.076
12	0.197	0.062	0.155
13	0.272	0.076	0.350
14	0.221	0.070	0.235
15	-0.249	0.040	-0.921
16	-0.287	0.018	-0.829
17	0.401	0.066	0.541
18	-0.430	0.016	-1.440
19	-0.215	0.073	-1.025
20	0.166	0.070	0.052
21	0.111	0.055	-0.107
22	0.057	0.038	-0.175
23	0.130	0.045	-0.043
24	0.095	0.037	-0.116
25	0.085	0.035	-0.128
26	0.118	0.043	-0.054
27	-0.409	0.019	-1.299
28	0.148	0.180	-0.240
29	0.071	0.032	-0.147
30	0.111	0.036	-0.060
31	0.101	0.040	-0.075
32	0.175	0.053	0.056
33	0.104	0.051	-0.121
34	0.142	0.056	-0.073
35	0.233	0.059	0.135
36	0.228	0.051	0.159
37	0.276	0.071	0.317
38	0.214	0.053	0.181
39	0.122	0.044	-0.028
40	0.132	0.041	-0.011
41	0.129	0.039	-0.013
42	0.113	0.038	-0.021
43	0.145	0.046	-0.012
44	0.185	0.058	0.054
45	0.122	0.039	-0.049
46	0.135	0.043	-0.017
47	-1.126	0.004	-3.166
48	-0.509	0.015	-1.702
49	-0.475	0.020	-1.812
50	-0.310	0.071	-1.652
51	-0.469	0.018	-1.851
52	-0.530	0.012	-1.917
53	-0.484	0.045	-2.083
54	-0.338	0.219	-2.198
55	-0.376	0.082	-1.613
56	-0.593	0.018	-1.908
57	-0.619	0.017	-1.721
58	-0.641	0.011	-1.857
59	-0.653	0.015	-1.489
60	-0.674	0.010	-1.794
61	-0.631	0.010	-1.793
62	-0.600	0.012	-1.754
63	-0.618	0.015	-2.166
64	0.078	0.035	-0.144
65	-0.022	0.030	-0.347
66	-0.061	0.030	-0.548
67	-0.088	0.024	-0.531
68	-0.025	0.028	-0.273
69	0.171	0.048	0.033
70	-0.611	0.016	-1.079
71	-0.661	0.013	-1.275
72	0.183	0.050	0.046
73	-0.071	0.035	-0.184
74	0.040	0.042	-0.142
75	0.038	0.043	-0.043
76	0.124	0.106	0.102
77	0.068	0.068	0.035
78	0.093	0.089	0.057
79	0.117	0.137	0.098
80	0.030	0.044	-0.047
81	0.074	0.093	0.029
82	0.064	0.210	-0.180
83	0.064	0.108	0.057
84	0.019	0.056	-0.045
85	0.072	0.122	0.065
86	0.037	0.145	0.054
87	-1.178	0.034	-0.463
88	-1.067	0.024	-0.695
89	-0.883	0.012	-1.473
90	0.022	0.100	-0.003
91	-0.064	0.047	-0.168
92	0.004	0.146	-0.003
93	0.006	0.165	0.004
94	0.000	0.188	-0.007
95	0.030	0.182	-0.026
96	0.000	0.120	0.000
97	-0.006	0.196	-0.001
98	0.001	0.169	0.002
99	-0.003	0.140	-0.002
100	2.005	0.795	0.170

Supplementary Figure 16: Conservation states enrichments for evolutionary constrained element calls and average constraint scores. (a) Each row corresponds to a conservation state and each column corresponds to a different constrained element set. The values correspond to the fold enrichment for bases in a constrained element set for the conservation state. The constrained element sets are from left to right GERP++[4], PhastCons[5], SiPhy-omega, and SiPhy-pi[6,7]. The bottom row gives the percentage of the genome of each constrained element set. **(b)** Each row corresponds to a conservation state and each column corresponds to a different score of constraint. The values correspond to the average constraint score in the conservation state. The constraint scores are from left to right: PhyloP[8], PhastCons[5], and GERP++[4].

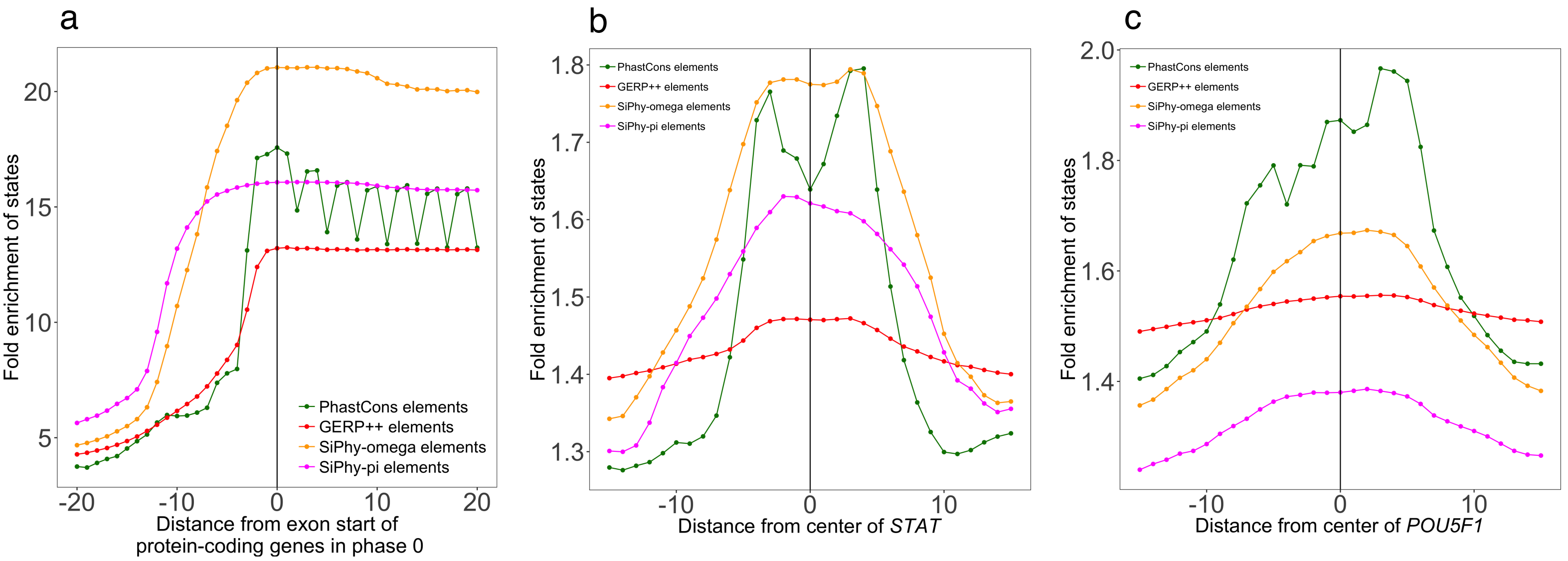

Supplementary Figure 17: Positional enrichment of constrained element sets. Analogous to what was shown for conservation states in **Fig. 3a,e,f**, the graphs show the positional fold enrichments of GERP++, PhastCons, SiPhy-omega and SiPhy-pi constrained elements calls around **(a)** the start of exons of protein coding genes, **(b)** center of instances of a *STAT* motif, and **(c)** center of instances of a *POU5F1* motif. Of these only PhastCons element calls are able to exhibit relevant single nucleotide enrichment variation, as was seen with the conservation states.

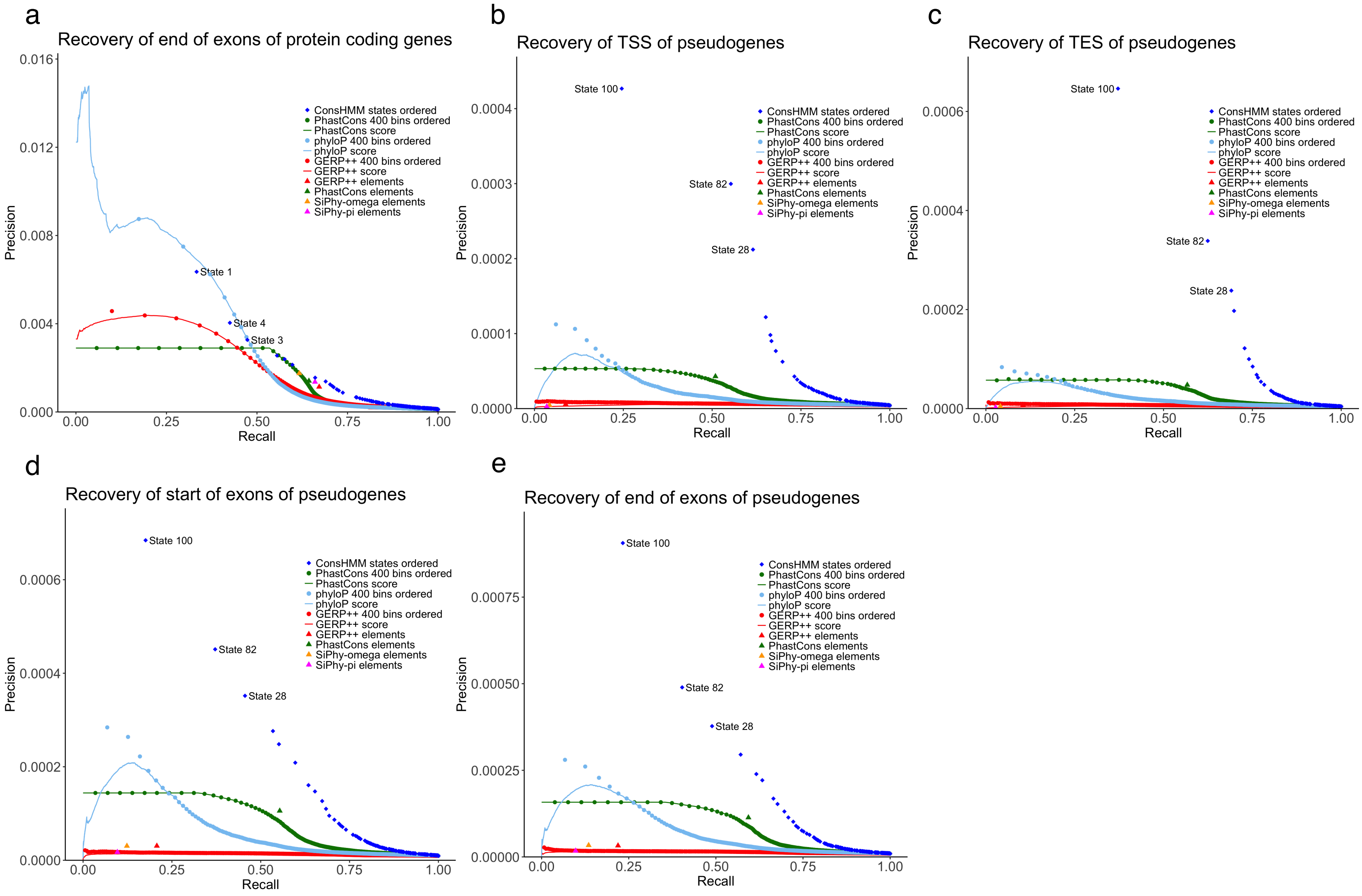

Supplementary Figure 18: Precision-recall recovery of conservation states and constrained element and scores for additional gene annotations. Analogous precision-recall plots to those shown in **Fig. 5a-c**, shown here for **(a)** ends of exons of protein coding genes, **(b)** TSS of pseudogenes, **(c)** TES of pseudogenes, **(d)** start of exons of pseudogenes, and **(e)** end of exons of pseudogenes. Precision-recall values were computed using the same procedure as for **Fig. 5** (**Methods**). The first few conservation states added are labeled.

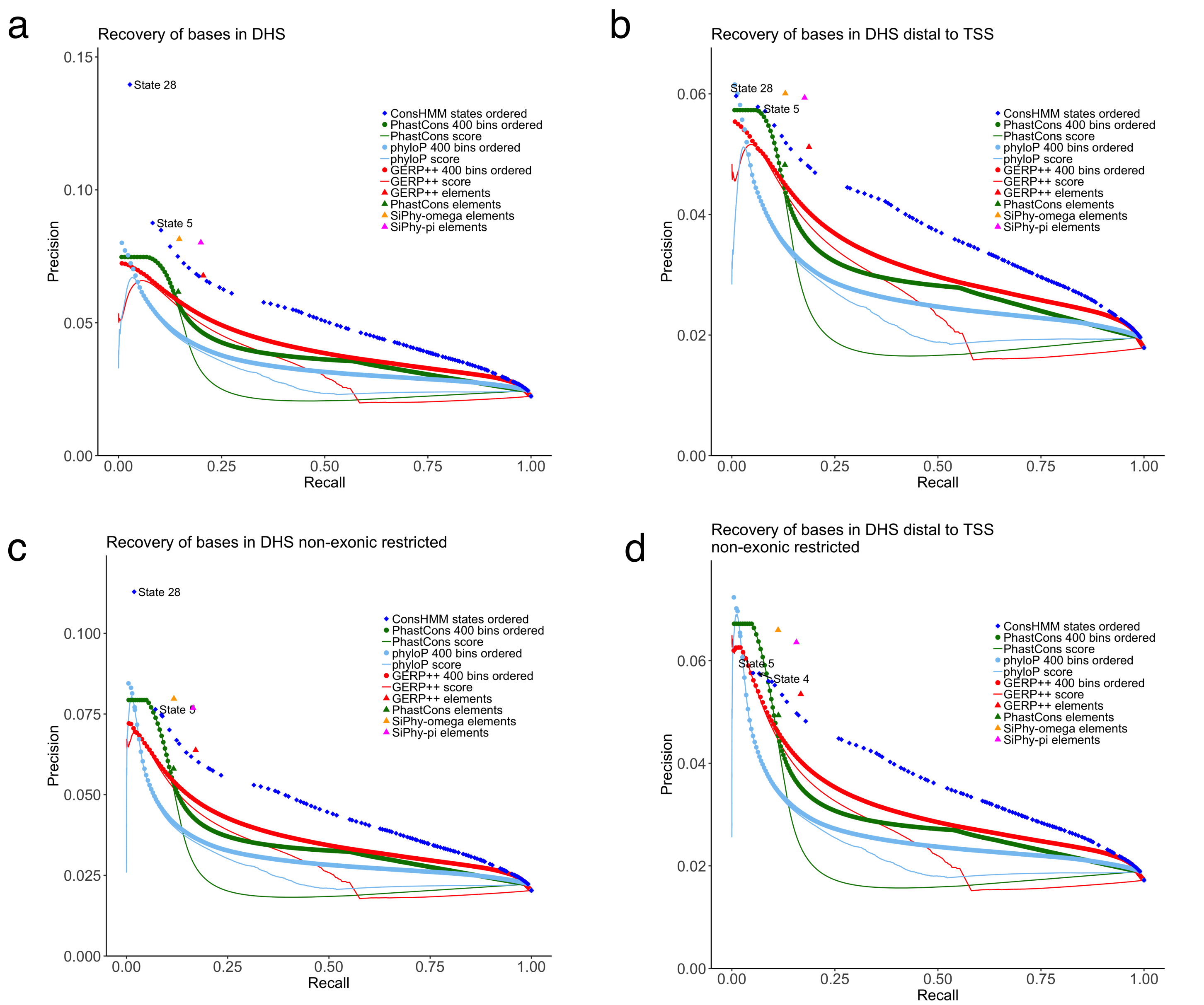

Supplementary Figure 19: Precision-recall recovery of conservation states and constrained element sets and scores for a concatenation of DHS bases in 53 cell and tissue types. Analogous precision-recall plots to those shown in **Fig. 5a-c** and **Supplementary Fig. 18** shown here for DHS bases concatenated across experiments shown **(a)** without restriction and **(b-d)** with the following restrictions for the target and background: **(b)** bases more than 2kb away from a TSS, **(c)** non-exonic bases, **(d)** non-exonic bases more than 2kb away from a TSS.

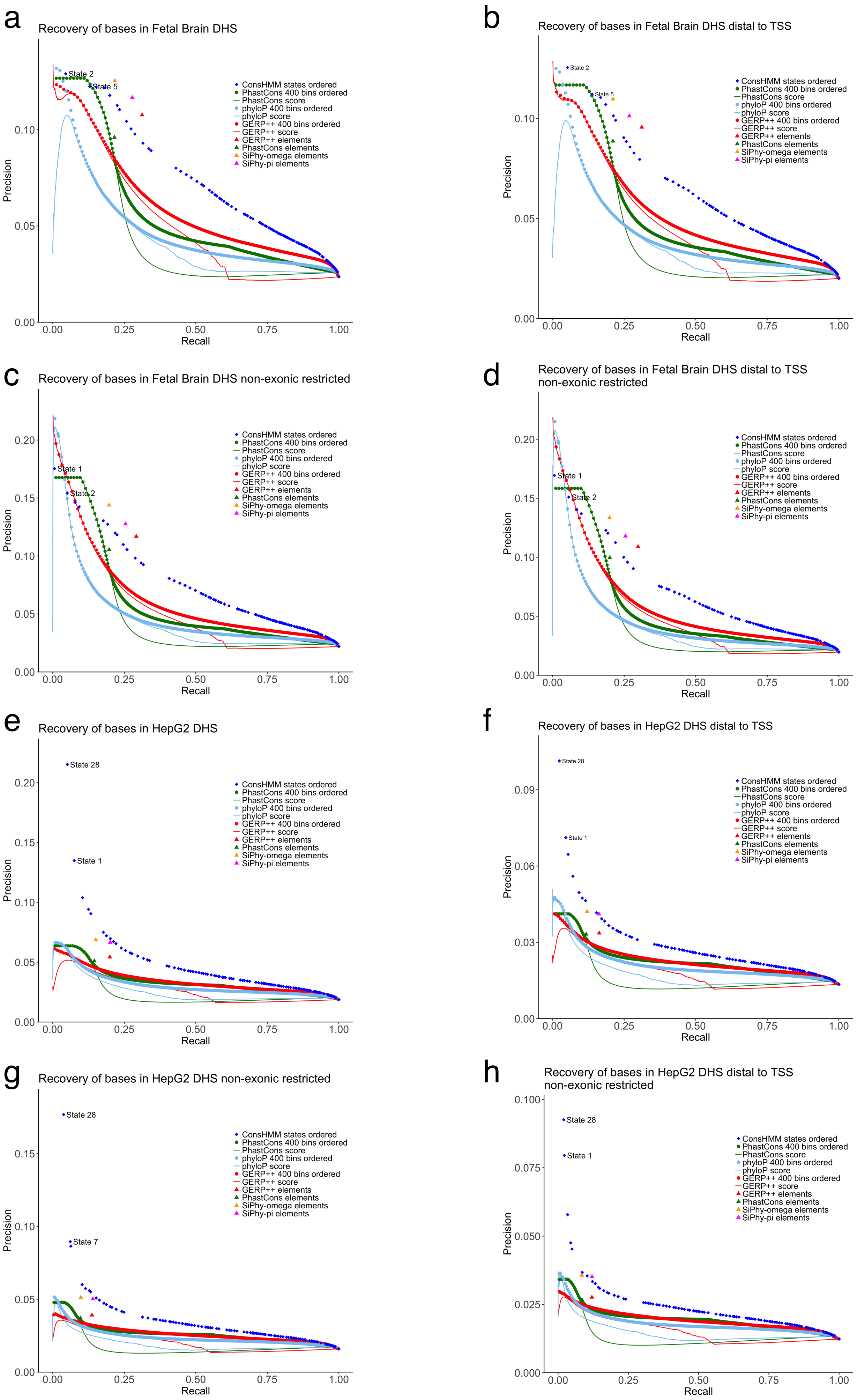

Supplementary Figure 20: Precision-recall recovery of conservation states and constrained element and scores for DHS in two cell types. Analogous precision-recall plots to those shown in **Fig. 5a-c, Supplementary Figs. 18** and **19** shown here for **(a)** Fetal Brain DHS, **(b)** Fetal Brain DHS when restricting target and background to bases more than 2kb away from a TSS, **(c)** Fetal Brain DHS when restricting target and background to non-exonic bases of the genome, **(d)** Fetal Brain DHS when restricting target and background to non-exonic bases of the genome that are more than 2kb away from a TSS, **(e)** HepG2 DHS, **(f)** HepG2 DHS when restricting target and background to bases more than 2kb away from a TSS, **(g)** HepG2 when restricting target and background to non-exonic bases of the genome, **(h)** HepG2 DHS when restricting target and background to non-exonic bases of the genome that are more than 2kb away from a TSS.

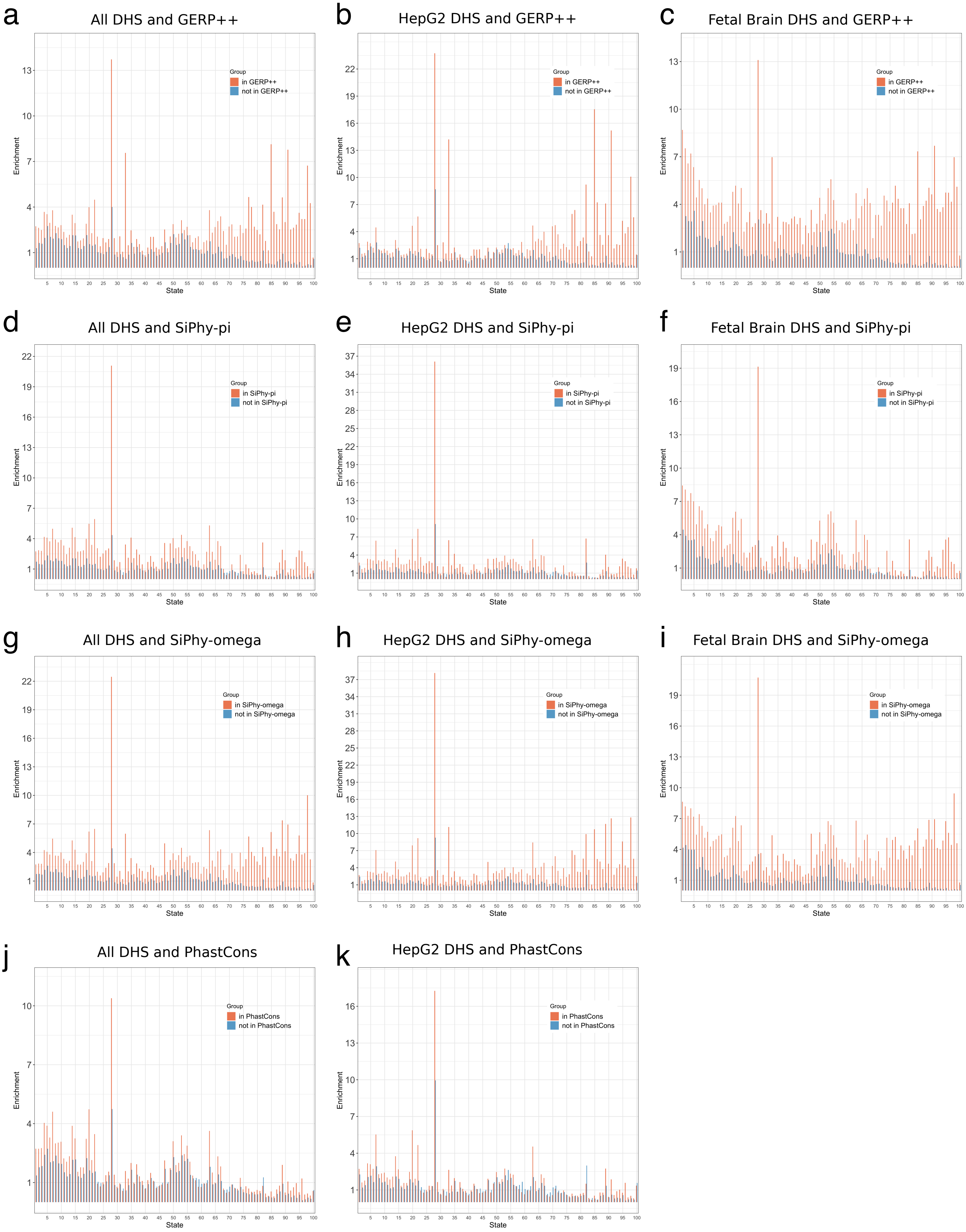

Supplementary Figure 21: Enrichment for non-exonic DHS conditioned on conservation state and constrained element sets. Analogous graphs to **Fig. 5d**, showing enrichments for bases in DHS in the non-exonic portion of each conservation state conditioned on whether it is in a constrained element or not. Enrichments are shown here for **(a-c)** bases in and out of GERP++ elements for **(a)** geometric mean over 53 cell and tissue types, **(b)** HepG2 DHS, **(c)** Fetal Brain DHS; **(d-f)** bases in and out of SiPhy-pi elements for **(d)** geometric mean over 53 cell and tissue types **(e)** HepG2 DHS, **(f)** Fetal Brain DHS; **(g-i)** bases in and out of SiPhy-omega elements for **(g)** geometric mean over 53 cell and tissue types **(h)** HepG2 DHS, **(i)** Fetal Brain DHS; **(j-k)** bases in and out of PhastCons elements for **(j)** geometric mean over 53 cell and tissue types **(k)** HepG2 DHS.

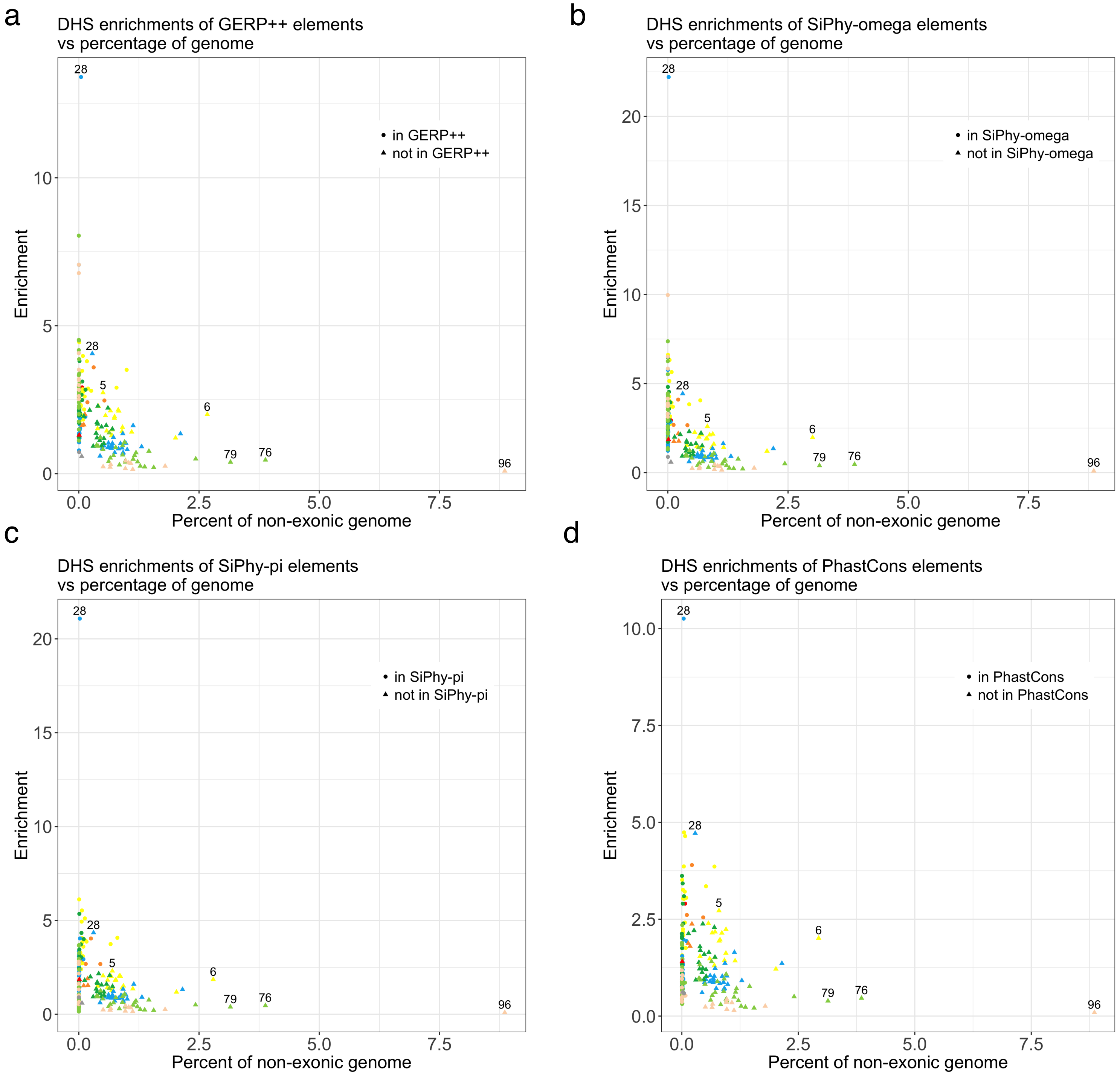

Supplementary Figure 22: Enrichment for non-exonic DHS conditioned on conservation state and constrained element sets versus percent of non-exonic genome covered. The enrichments in **Supplementary Fig. 21a,d,g,j** are shown here on the y-axis (geometric mean over 53 cell and tissue types), with the x-axis corresponding to the median percentage of the non-exonic genome covered by the bases falling in each category across 53 cell and tissue types. The coloring of a point corresponds to the coloring of states in **Fig. 2**. The shape of a point corresponds to whether it is inside or outside a constrained element set according to the legend shown. Labeled points either have an enrichment >10 fold, cover >2.5% of the non-exonic genome, or are subsets of states not in a constrained element set with an enrichment >2 fold. The figure shows substantial variation of enrichments for bases both inside and outside of constrained elements depending on the conservation state, including for subsets covering non-negligible portions of the non-exonic genome. The constrained element sets used for each panel are **(a)** GERP++ elements, **(b)** SiPhy-pi elements, **(c)** SiPhy-omega elements, and **(d)** PhastCons elements.

a

	All CNEEs	Euteleostomi	Tetrapod	Amniote	Mammal	Theria	Eutheria	Boreoeutheria	Euarchontoglire	Hominini
1	2.43	65.12	5.69	0.63	0.02	0.02	0.01	0.26	0.00	0.00
2	16.41	42.84	37.42	53.14	1.00	0.46	0.06	0.91	0.15	0.04
3	6.92	17.39	11.74	24.50	0.64	0.30	0.04	0.55	0.11	0.01
4	10.74	6.03	5.59	20.54	21.60	8.33	1.21	1.73	0.01	0.12
5	12.88	1.39	1.08	4.16	30.64	32.56	3.49	0.87	0.10	0.05
6	3.80	0.20	0.11	0.37	2.06	2.39	10.52	0.97	0.06	0.08
7	2.30	1.83	1.10	6.30	2.17	1.54	0.63	0.92	0.13	0.02
8	4.04	0.56	0.39	1.34	9.74	10.29	0.90	0.35	0.02	0.05
9	1.43	0.10	0.05	0.15	0.84	1.09	3.80	0.33	0.01	0.04
10	0.87	0.09	0.05	0.14	0.95	1.33	1.59	0.29	0.04	0.02
11	0.66	0.09	0.05	0.10	0.58	0.77	1.36	0.79	0.06	0.03
12	0.95	0.07	0.04	0.09	0.67	1.11	2.12	1.24	0.04	0.06
13	1.21	0.08	0.04	0.11	0.68	1.18	3.09	0.44	0.05	0.05
14	1.03	0.11	0.06	0.11	0.76	1.17	2.39	0.50	0.04	0.06
15	0.83	0.07	0.03	0.09	0.49	0.67	2.19	0.23	0.02	0.02
16	0.20	0.02	0.01	0.02	0.05	0.09	0.55	0.71	0.01	0.02
17	0.70	0.05	0.04	0.06	0.18	0.34	1.90	2.40	0.05	0.07
18	0.20	0.02	0.01	0.02	0.07	0.12	0.54	0.45	0.04	0.02
19	1.76	0.17	0.11	0.37	2.64	3.08	2.45	0.28	0.04	0.02
20	1.05	0.09	0.08	0.18	1.13	1.43	1.97	0.59	0.06	0.03
21	0.80	0.08	0.06	0.15	0.90	1.08	1.48	0.50	0.07	0.05
22	0.33	0.04	0.04	0.04	0.16	0.28	0.81	0.65	0.17	0.09
23	0.28	0.05	0.05	0.03	0.05	0.06	0.66	2.37	0.01	0.28
24	0.23	0.01	0.04	0.02	0.04	0.05	0.56	1.63	0.11	0.11
25	0.24	0.02	0.02	0.02	0.02	0.03	0.48	3.10	0.07	0.10
26	0.39	0.03	0.02	0.04	0.15	0.20	1.07	0.69	0.01	0.07
27	0.20	0.03	0.02	0.02	0.09	0.13	0.53	0.48	0.02	0.03
28	1.65	1.82	3.39	2.39	1.27	1.22	0.46	5.55	5.01	0.28
29	0.22	0.01	0.01	0.02	0.04	0.06	0.57	1.40	0.42	0.09
30	0.23	0.03	0.05	0.02	0.05	0.07	0.57	1.44	0.05	0.08
31	0.24	0.04	0.05	0.02	0.06	0.07	0.62	1.17	0.02	0.20
32	0.25	0.02	0.11	0.06	0.01	0.02	0.22	5.49	0.11	0.40
33	0.30	0.04	0.20	0.04	0.07	0.07	0.43	4.02	1.21	0.32
34	0.61	0.06	0.11	0.06	0.25	0.44	1.40	2.43	1.87	0.22
35	0.57	0.08	0.05	0.07	0.33	0.41	1.44	0.80	0.03	0.13
36	0.37	0.06	0.06	0.03	0.04	0.07	0.85	3.62	0.04	0.17
37	0.92	0.09	0.05	0.08	0.42	0.68	2.44	1.14	0.05	0.08
38	0.51	0.07	0.03	0.05	0.23	0.33	1.38	0.59	0.04	0.08
39	0.41	0.03	0.06	0.04	0.17	0.24	0.95	1.98	0.03	0.10
40	0.25	0.03	0.03	0.02	0.02	0.03	0.43	3.85	0.06	0.15
41	0.31	0.04	0.02	0.02	0.06	0.10	0.69	3.01	0.09	0.08
42	0.29	0.04	0.02	0.02	0.05	0.07	0.75	1.73	0.09	0.34
43	0.30	0.04	0.03	0.03	0.05	0.06	0.79	1.66	0.02	0.15
44	0.33	0.05	0.14	0.04	0.03	0.04	0.42	6.27	0.09	0.38
45	0.24	0.03	0.04	0.02	0.03	0.05	0.55	2.22	0.18	0.20
46	0.31	0.04	0.04	0.03	0.05	0.08	0.78	2.10	0.05	0.17
47	0.07	0.01	0.01	0.01	0.04	0.05	0.16	0.15	0.01	0.02
48	0.12	0.02	0.03	0.01	0.03	0.05	0.28	0.65	0.02	0.04
49	0.22	0.03	0.02	0.02	0.12	0.17	0.56	0.30	0.02	0.03
50	1.60	0.18	0.12	0.49	2.57	2.73	2.04	0.27	0.01	0.04
51	0.21	0.02	0.01	0.02	0.08	0.14	0.55	0.43	0.01	0.04
52	0.14	0.01	0.01	0.01	0.05	0.09	0.36	0.33	0.06	0.02
53	1.01	0.16	0.10	0.38	1.58	1.65	1.27	0.20	0.03	0.02
54	3.08	8.06	5.55	9.78	0.87	0.37	0.07	0.93	0.14	0.03
55	2.01	0.23	0.20	0.79	3.35	3.34	2.36	0.53	0.02	0.02
56	0.23	0.02	0.02	0.02	0.11	0.18	0.54	0.70	0.17	0.03
57	0.15	0.02	0.03	0.02	0.06	0.08	0.36	0.77	0.01	0.03
58	0.09	0.02	0.02	0.01	0.03	0.05	0.20	0.49	0.06	0.04
59	0.08	0.02	0.05	0.01	0.02	0.01	0.14	1.08	0.03	0.10
60	0.06	0.01	0.01	0.01	0.01	0.01	0.11	0.86	0.02	0.03
61	0.06	0.01	0.02	0.01	0.01	0.02	0.14	0.34	0.05	0.02
62	0.08	0.01	0.02	0.01	0.02	0.02	0.19	0.53	0.03	0.03
63	0.20	0.03	0.02	0.04	0.20	0.23	0.38	0.25	0.10	0.01
64	0.25	0.01	0.03	0.03	0.10	0.12	0.59	1.38	0.66	0.18
65	0.13	0.02	0.06	0.03	0.05	0.04	0.32	0.18	1.97	0.61
66	0.27	0.06	0.04	0.08	0.23	0.26	0.57	0.12	1.72	0.08
67	0.14	0.06	0.07	0.05	0.06	0.07	0.17	0.48	13.81	0.41
68	0.11	0.03	0.16	0.05	0.01	0.01	0.08	0.54	13.31	0.60
69	0.19	0.04	0.21	0.06	0.01	0.01	0.19	3.40	0.76	0.59
70	0.08	0.02	0.15	0.03	0.01	0.01	0.06	1.18	0.19	0.25
71	0.07	0.01	0.09	0.02	0.02	0.02	0.09	0.81	0.34	0.21
72	0.23	0.02	0.16	0.05	0.02	0.03	0.26	3.96	0.99	0.60
73	0.15	0.06	0.22	0.05	0.01	0.01	0.10	2.71	0.88	0.97
74	0.15	0.05	0.25	0.21	0.04	0.04	0.08	0.33	2.51	7.05
75	0.16	0.16	0.48	0.12	0.03	0.02	0.09	0.69	2.63	3.11
76	0.14	0.02	0.41	0.25	0.02	0.02	0.04	0.17	0.45	5.17
77	0.16	0.04	0.81	0.13	0.01	0.01	0.03	0.30	1.30	3.44
78	0.17	0.02	0.53	0.31	0.01	0.00	0.04	0.48	1.42	3.82
79	0.17	0.01	0.47	0.40	0.01	0.01	0.03	0.25	0.42	3.40
80	0.14	0.06	0.47	0.12	0.02	0.01	0.06	0.61	5.97	1.83
81	0.26	0.08	0.97	0.28	0.05	0.01	0.08	1.61	0.92	2.10
82	3.31	0.94	22.57	0.63	0.54	0.07	1.05	3.13	11.88	4.50
83	0.20	0.01	0.79	0.41	0.00	0.00	0.01	0.14	0.28	4.12
84	0.25	0.05	1.07	0.21	0.02	0.01	0.05	1.59	4.02	2.60
85	0.19	0.01	0.93	0.28	0.00	0.00	0.01	0.25	0.68	2.10
86	0.16	0.01	0.37	0.50	0.00	0.00	0.01	0.14	0.23	2.04
87	0.12	0.02	0.61	0.16	0.01	0.01	0.02	0.15	0.51	0.69
88	0.10	0.05	0.42	0.11	0.02	0.01	0.03	0.12	0.49	1.16
89	0.07	0.02	0.09	0.02	0.03	0.03	0.09	0.68	0.85	0.11
90	0.22	0.06	0.66	0.49	0.02	0.01	0.03	0.36	1.14	2.55
91	0.21	0.06	0.56	0.13	0.03	0.03	0.10	2.18	4.96	0.90
92	0.15	0.01	0.42	0.40	0.02	0.01	0.03	0.22	0.59	1.16
93	0.18	0.01	0.46	0.51	0.01	0.01	0.02	0.17	0.48	1.07
94	0.15	0.01	0.45	0.40	0.02	0.02	0.03	0.11	0.26	0.65
95	0.78	0.12	5.00	0.43	0.17	0.04	0.13	0.58	4.27	1.15
96	0.02	0.00	0.09	0.02	0.00	0.00	0.00	0.01	0.05	0.02
97	0.11	0.00	0.48	0.22	0.00	0.00	0.01	0.07	0.09	0.35
98	0.13	0.01	0.46	0.35	0.01	0.00	0.00	0.14	0.10	0.37
99	0.16	0.01	0.64	0.32	0.00	0.00	0.01	0.22	0.73	0.92
100	6.87	3.73	50.95	0.41	0.20	0.07	1.69	5.16	28.81	11.10
Base	2.70	0.07	0.31	0.54	0.52	0.42	0.75	0.08	0.01	0.01

Enrichments of Lowe et al.[9] CNEEs

b

	All CNEEs	Euteleostomi	Tetrapod	Amniote	Mammal	Theria	Eutheria	Boreoeutheria	Euarchontoglire	Hominini
1	3.20	70.28	8.59	0.76	0.02	0.02	0.01	0.55	0.01	0.00
2	20.88	45.36	56.45	62.31	1.08	0.51	0.08	1.87	0.36	0.15
3	7.98	16.85	15.96	26.21	0.58	0.28	0.04	0.98	0.25	0.02
4	12.65	5.43	7.68	22.61	22.67	8.90	1.55	3.36	0.03	0.39
5	15.37	1.26	1.47	4.61	32.90	35.38	4.53	1.60	0.16	0.16
6	4.09	0.14	0.12	0.37	2.04	2.30	12.55	1.54	0.09	0.23
7	2.10	0.96	1.00	5.63	1.75	1.23	0.57	1.33	0.22	0.03
8	3.92	0.36	0.41	1.20	8.60	9.06	0.91	0.49	0.01	0.10
9	1.24	0.05	0.04	0.12	0.70	0.89	3.62	0.39	0.01	0.08
10	0.73	0.04	0.04	0.12	0.77	1.08	1.40	0.29	0.02	0.04
11	0.53	0.04	0.04	0.07	0.50	0.63	1.14	0.86	0.09	0.05
12	0.84	0.03	0.04	0.08	0.58	0.98	1.98	1.71	0.04	0.17
13	1.11	0.04	0.04	0.10	0.60	1.05	3.09	0.56	0.03	0.10
14	0.89	0.06	0.05	0.09	0.65	1.01	2.18	0.57	0.03	0.17
15	0.67	0.03	0.02	0.07	0.38	0.49	1.95	0.24	0.01	0.05
16	0.11	0.01	0.01	0.01	0.03	0.05	0.33	0.56	0.01	0.03
17	0.57	0.02	0.03	0.05	0.15	0.29	1.69	3.04	0.04	0.15
18	0.13	0.01	0.00	0.01	0.05	0.08	0.37	0.39	0.02	0.03
19	1.62	0.10	0.11	0.32	2.29	2.67	2.35	0.33	0.05	0.03
20	0.96	0.05	0.08	0.16	1.03	1.30	1.84	0.80	0.07	0.08
21	0.70	0.04	0.05	0.13	0.79	0.94	1.30	0.59	0.08	0.09
22	0.22	0.01	0.02	0.03	0.12	0.22	0.55	0.58	0.20	0.22
23	0.15	0.01	0.01	0.01	0.03	0.04	0.39	1.90	0.00	0.56
24	0.11	0.01	0.01	0.01	0.02	0.03	0.32	1.26	0.08	0.18
25	0.14	0.01	0.01	0.01	0.01	0.02	0.31	3.03	0.09	0.18
26	0.26	0.01	0.01	0.03	0.12	0.16	0.75	0.67	0.01	0.14
27	0.12	0.01	0.01	0.01	0.06	0.08	0.32	0.37	0.01	0.06
28	1.56	1.25	3.57	2.21	1.06	1.07	0.46	8.95	9.58	0.30
29	0.13	0.01	0.01	0.01	0.02	0.04	0.38	1.28	0.61	0.19
30	0.12	0.00	0.01	0.01	0.04	0.05	0.33	1.15	0.05	0.13
31	0.12	0.00	0.01	0.01	0.04	0.04	0.34	0.90	0.01	0.19
32	0.12	0.00	0.02	0.01	0.01	0.01	0.13	4.60	0.14	0.26
33	0.16	0.01	0.05	0.02	0.05	0.07	0.26	3.59	1.45	0.62
34	0.47	0.03	0.06	0.05	0.21	0.38	1.15	3.17	3.59	0.60
35	0.41	0.04	0.04	0.05	0.27	0.33	1.07	0.74	0.02	0.19
36	0.23	0.02	0.02	0.02	0.02	0.05	0.59	3.60	0.03	0.36
37	0.77	0.05	0.03	0.06	0.35	0.57	2.20	1.50	0.04	0.19
38	0.36	0.02	0.02	0.04	0.18	0.27	1.03	0.55	0.04	0.12
39	0.27	0.01	0.03	0.03	0.13	0.18	0.64	2.41	0.02	0.19
40	0.13	0.01	0.01	0.01	0.01	0.02	0.27	3.31	0.07	0.32
41	0.21	0.01	0.01	0.01	0.04	0.07	0.52	3.22	0.03	0.21
42	0.16	0.01	0.01	0.01	0.03	0.05	0.48	1.51	0.08	0.88
43	0.17	0.01	0.01	0.02	0.03	0.04	0.50	1.45	0.00	0.22
44	0.17	0.01	0.04	0.01	0.02	0.03	0.25	5.38	0.05	0.55
45	0.12	0.01	0.01	0.01	0.02	0.03	0.31	1.74	0.13	0.30
46	0.17	0.01	0.02	0.01	0.03	0.05	0.46	1.81	0.05	0.29
47	0.03	0.00	0.00	0.00	0.02	0.03	0.08	0.08	0.00	0.02
48	0.06	0.00	0.01	0.00	0.02	0.03	0.16	0.53	0.00	0.07
49	0.14	0.01	0.01	0.01	0.08	0.11	0.39	0.28	0.01	0.03
50	1.54	0.11	0.13	0.45	2.31	2.46	2.03	0.34	0.01	0.07
51	0.15	0.01	0.01	0.01	0.06	0.11	0.42	0.42	0.00	0.07
52	0.08	0.00	0.00	0.01	0.03	0.06	0.24	0.28	0.03	0.04
53	0.88	0.09	0.10	0.32	1.31	1.36	1.15	0.19	0.02	0.03
54	3.30	7.22	7.14	9.80	0.70	0.29	0.06	1.48	0.31	0.08
55	2.02	0.16	0.22	0.77	3.12	3.13	2.46	0.80	0.02	0.04
56	0.17	0.01	0.01	0.01	0.09	0.14	0.43	0.87	0.27	0.06
57	0.09	0.00	0.01	0.01	0.04	0.06	0.23	0.81	0.00	0.05
58	0.04	0.00	0.01	0.00	0.02	0.03	0.10	0.32	0.02	0.03
59	0.03	0.00	0.01	0.00	0.01	0.01	0.07	0.72	0.00	0.07
60	0.02	0.00	0.00	0.00	0.00	0.00	0.05	0.58	0.01	0.05
61	0.02	0.00	0.00	0.00	0.01	0.01	0.07	0.23	0.04	0.02
62	0.03	0.00	0.00	0.00	0.01	0.01	0.09	0.35	0.01	0.03
63	0.14	0.01	0.01	0.03	0.14	0.16	0.27	0.24	0.08	0.01
64	0.15	0.00	0.01	0.02	0.08	0.10	0.36	1.08	0.90	0.23
65	0.05	0.01	0.01	0.01	0.03	0.02	0.13	0.09	1.05	0.33
66	0.19	0.03	0.03	0.06	0.17	0.20	0.39	0.09	1.07	0.09
67	0.08	0.03	0.03	0.03	0.04	0.05	0.10	0.42	12.89	0.21
68	0.03	0.00	0.00	0.01	0.00	0.01	0.03	0.34	9.65	0.55
69	0.07	0.00	0.01	0.01	0.01	0.01	0.09	2.47	0.51	0.36
70	0.02	0.00	0.01	0.00	0.00	0.00	0.02	0.72	0.06	0.07
71	0.02	0.00	0.00	0.00	0.01	0.01	0.04	0.45	0.22	0.11
72	0.10	0.00	0.02	0.01	0.01	0.02	0.14	2.86	1.05	0.59
73	0.05	0.01	0.01	0.01	0.00	0.00	0.05	2.04	0.96	0.50
74	0.05	0.02	0.02	0.02	0.03	0.03	0.04	0.19	1.59	8.74
75	0.04	0.06	0.09	0.02	0.02	0.01	0.05	0.46	1.88	1.50
76	0.02	0.01	0.02	0.01	0.01	0.01	0.02	0.06	0.18	3.70
77	0.02	0.01	0.04	0.01	0.00	0.00	0.01	0.16	1.29	2.40
78	0.02	0.01	0.04	0.01	0.00	0.00	0.01	0.32	1.10	3.36
79	0.02	0.00	0.02	0.01	0.00	0.01	0.01	0.08	0.25	3.82
80	0.03	0.03	0.05	0.01	0.01	0.01	0.03	0.41	5.25	0.95
81	0.10	0.03	0.40	0.04	0.03	0.00	0.06	1.34	1.33	2.58
82	1.29	0.32	7.41	0.35	0.27	0.05	1.11	4.77	20.72	11.44
83	0.01	0.00	0.01	0.01	0.00	0.00	0.00	0.06	0.08	3.78
84	0.06	0.03	0.15	0.02	0.01	0.00	0.03	1.17	2.62	1.96
85	0.01	0.00	0.01	0.00	0.00	0.00	0.00	0.09	0.27	2.05
86	0.00	0.00	0.01	0.01	0.00	0.00	0.00	0.02	0.01	1.25
87	0.01	0.01	0.05	0.00	0.00	0.00	0.01	0.08	0.30	0.38
88	0.01	0.01	0.03	0.01	0.01	0.01	0.02	0.06	0.27	0.60
89	0.03	0.00	0.01	0.01	0.02	0.02	0.05	0.49	0.62	0.08
90	0.02	0.01	0.04	0.02	0.01	0.01	0.02	0.15	0.74	1.06
91	0.06	0.01	0.05	0.02	0.01	0.02	0.05	1.76	3.19	0.76
92	0.02	0.00	0.02	0.01	0.01	0.01	0.02	0.09	0.28	0.70
93	0.01	0.00	0.02	0.00	0.00	0.01	0.02	0.08	0.29	1.13
94	0.02	0.01	0.02	0.01	0.02	0.02	0.02	0.05	0.40	0.08
95	0.44	0.06	3.37	0.13	0.08	0.03	0.10	0.62	4.75	1.34
96	0.00	0.00	0.01	0.00	0.00	0.00	0.00	0.00	0.00	0.00
97	0.01	0.00	0.02	0.00	0.00	0.00	0.00	0.02	0.04	0.14
98	0.00	0.00	0.01	0.00	0.00	0.00	0.00	0.02	0.09	0.04
99	0.01	0.00	0.02	0.00	0.00	0.00	0.01	0.08	0.27	0.06
100	8.23	2.97	71.01	0.45	0.21	0.08	2.30	10.60	68.10	44.34
Base	2.03	0.06	0.20	0.44	0.44	0.34	0.51	0.04	0.003	0.003

Enrichments of Lowe et al.[9] CNEEs overlapping PhastCons elements called on 100-way alignment

c

CNEE	Non-Exonic %	CpG Island
Euteleostomi	0.07	4.8
Tetrapod	0.29	6.7
Amniote	0.56	2.8
Mammal	0.53	2.8
Theria	0.43	3.8
Eutheria	0.77	1.7
Boreoeutheria	0.08	2.5
Euarchontoglire	0.01	1.4
Hominini	0.01	0.2
No assignment	97.28	0.9
Base	100	0.43

Enrichments of Lowe et al.[9] CNEEs

d

CNEE	Non-Exonic %	CpG Island
Euteleostomi	0.06	3.0
Tetrapod	0.18	3.5
Amniote	0.45	1.7
Mammal	0.45	1.6
Theria	0.35	2.4
Eutheria	0.52	1.2
Boreoeutheria	0.03	2.2
Euarchontoglire	0.00	0.9
Hominini	0.00	0.4
No assignment	97.96	1.0
Base	100	0.43

Enrichments of Lowe et al.[9] CNEEs overlapping PhastCons elements called on 100-way alignment

Supplementary Figure 23: Relationship between conservation states and CNEEs from Ref. 9. (a,b) The conservation state enrichments for **(a)** the set of CNEEs defined in Ref. 9 and **(b)** the subset restricted to those that overlap PhastCons element bases called on the 100-way alignment in which the conservation states were defined. The first column of each panel gives the enrichment of all CNEEs included, followed by the enrichments of subset of CNEEs, based on the branch of origin of the CNEE. The second through last columns are sorted in order of distance of branch point to human. The last row gives the % of the genome covered by the annotation in the column. **(c,d)** The enrichments within non-exonic regions of bases in CpG islands of **(c)** the set of CNEEs defined in Ref. 9 and **(d)** the subset restricted to those that overlap PhastCons element bases called on the 100-way alignment in which the conservation states were defined. The first column of each panel gives the percentage of non-exonic bases found in each subset, and the second columns gives the enrichment for bases within non-exonic CpG islands. The last row gives the % of the non-exonic genome covered by the annotation in the column.

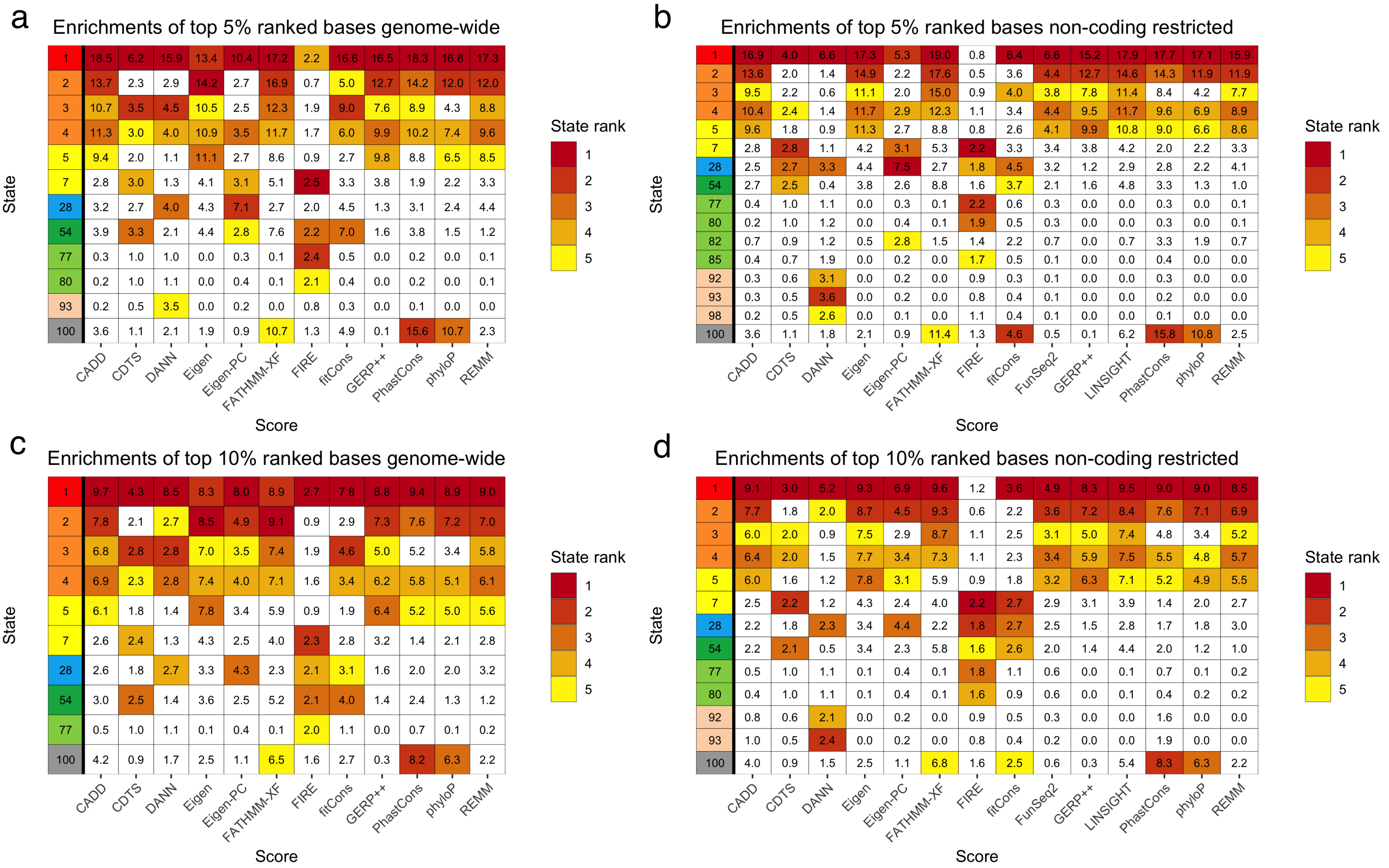

Supplementary Figure 24: Enrichments of selected conservation states for bases prioritized by variant prioritization scores. Analogous figures to those shown in **Fig. 6a,b** of conservation state enrichments of top 1% bases of variant prioritization scores except shown here for: **(a)** bases ranked in top 5% genome-wide, **(b)** bases ranked in the top 5% of the genome restricted to non-coding bases, **(c)** bases ranked in the top 10% genome-wide, and **(d)** bases ranked in the top 10% of the genome restricted to non-coding bases. Only states which were one of the five most enriched states by at least one variant prioritization score are shown. Coloring of enrichments is based on the rank of the state for the score as indicated in the color legend.

a

Enrichments of top 1% ranked bases genome-wide

State	Genome %	CADD 1.4	CADD 1.0	Eigen	Eigen-PC	DANN	GERP++	phyloP	PhastCons	REMM	fitCons	FIRE	FATHMM-XF	CDTS
1	0.60	77.2	43.7	17.5	1.9	72.6	54.5	68.0	37.7	56.0	73.7	0.8	58.4	12.6
2	0.84	19.7	21.5	32.9	1.1	8.4	30.5	26.7	24.6	21.0	9.5	0.5	36.1	2.5
3	0.46	14.6	8.0	13.6	2.0	16.7	10.3	3.3	10.6	9.0	31.0	1.7	10.8	5.8
4	0.60	17.3	13.7	16.2	3.2	13.9	16.8	11.9	13.8	15.3	15.9	1.8	12.8	5.5
5	1.66	5.9	10.8	14.8	3.3	1.7	12.3	6.6	10.6	10.6	1.7	0.9	4.7	2.9
6	3.59	0.6	2.1	1.6	1.9	0.2	0.5	1.5	2.1	2.2	0.2	0.8	0.4	1.5
7	0.85	1.7	1.9	3.6	5.8	1.7	2.0	1.1	1.4	2.6	2.1	3.2	1.7	5.7
8	0.73	0.5	2.6	1.9	2.4	0.6	1.2	0.4	1.5	1.0	1.1	1.5	0.6	2.3
9	0.90	0.1	0.8	0.4	1.7	0.1	0.1	0.0	0.4	0.2	0.2	1.0	0.1	1.4
10	0.86	0.1	0.6	0.3	1.8	0.1	0.0	0.0	0.2	0.1	0.3	1.1	0.1	1.5
11	0.75	0.1	0.5	0.3	1.5	0.1	0.1	0.1	0.2	0.2	0.2	1.0	0.1	1.2
12	0.75	0.1	0.6	0.2	1.2	0.1	0.1	0.2	0.3	0.3	0.1	0.6	0.1	0.9
13	1.24	0.1	0.7	0.4	1.3	0.1	0.1	0.3	0.4	0.5	0.1	0.6	0.1	1.0
14	0.94	0.1	0.6	0.6	2.5	0.1	0.1	0.2	0.3	0.5	0.2	1.4	0.2	1.9
15	0.77	0.0	0.5	0.3	2.2	0.1	0.0	0.0	0.2	0.1	0.2	1.2	0.1	1.7
16	0.72	0.0	0.2	0.1	1.2	0.0	0.0	0.0	0.0	0.0	0.1	0.7	0.1	0.9
17	2.14	0.0	0.4	0.1	1.0	0.1	0.0	0.1	0.2	0.2	0.1	0.6	0.1	0.8
18	0.94	0.0	0.2	0.1	1.3	0.0	0.0	0.0	0.0	0.0	0.1	0.8	0.1	1.0
19	1.07	0.1	1.1	0.6	2.1	0.2	0.1	0.0	0.5	0.2	0.5	1.3	0.2	1.8
20	1.03	0.4	0.7	1.1	2.4	0.4	0.3	0.3	0.5	0.8	0.2	0.9	0.2	2.1
21	0.64	0.1	0.6	0.3	1.5	0.1	0.2	0.2	0.3	0.3	0.2	0.9	0.1	1.2
22	0.41	0.1	0.2	0.3	2.1	0.2	0.0	0.0	0.1	0.2	0.1	1.0	0.1	1.7
23	0.63	0.0	0.1	0.0	1.0	0.1	0.0	0.0	0.0	0.0	0.1	1.1	0.0	0.8
24	0.53	0.0	0.1	0.0	0.7	0.0	0.0	0.0	0.0	0.0	0.0	0.8	0.0	0.6
25	0.61	0.0	0.1	0.0	0.7	0.0	0.0	0.0	0.0	0.0	0.0	0.6	0.0	0.6
26	0.72	0.0	0.2	0.1	0.8	0.1	0.0	0.0	0.1	0.1	0.0	0.9	0.1	0.7
27	0.96	0.0	0.1	0.1	1.1	0.0	0.0	0.0	0.0	0.0	0.1	1.1	0.1	0.9
28	0.44	4.5	0.9	6.3	18.4	8.5	0.1	2.4	3.1	3.7	5.9	1.9	1.8	8.3
29	0.92	0.0	0.1	0.1	0.7	0.1	0.0	0.0	0.0	0.0	0.0	0.4	0.0	0.5
30	0.94	0.0	0.1	0.0	0.3	0.0	0.0	0.0	0.0	0.0	0.0	0.5	0.0	0.4
31	0.63	0.0	0.1	0.0	0.7	0.1	0.0	0.0	0.0	0.0	0.1	0.7	0.0	0.6
32	0.93	0.0	0.0	0.0	0.6	0.1	0.0	0.0	0.0	0.0	0.0	0.6	0.0	0.6
33	0.45	0.0	0.1	0.1	0.6	0.2	0.0	0.0	0.1	0.0	0.1	0.7	0.0	0.5
34	0.51	0.0	0.4	0.1	0.7	0.1	0.0	0.1	0.2	0.1	0.1	0.9	0.1	0.6
35	1.20	0.0	0.3	0.2	1.7	0.1	0.0	0.0	0.1	0.1	0.1	1.5	0.1	1.3
36	1.34	0.0	0.2	0.0	0.6	0.0	0.0	0.0	0.0	0.0	0.0	0.8	0.0	0.5
37	2.30	0.0	0.5	0.2	1.0	0.1	0.0	0.1	0.3	0.2	0.1	1.0	0.1	0.8
38	0.91	0.0	0.3	0.1	0.7	0.1	0.0	0.0	0.1	0.1	0.1	0.7	0.1	0.7
39	0.70	0.0	0.2	0.0	0.5	0.0	0.0	0.0	0.1	0.0	0.1	0.6	0.1	0.5
40	0.76	0.0	0.1	0.0	0.4	0.0	0.0	0.0	0.0	0.0	0.0	0.5	0.0	0.4
41	0.70	0.0	0.2	0.0	0.7	0.0	0.0	0.0	0.1	0.0	0.0	0.4	0.0	0.5
42	0.74	0.0	0.2	0.1	1.3	0.1	0.0	0.0	0.0	0.0	0.1	0.8	0.1	0.9
43	1.00	0.0	0.1	0.0	0.7	0.0	0.0	0.0	0.0	0.0	0.1	0.8	0.1	0.7
44	1.02	0.0	0.1	0.0	0.8	0.1	0.0	0.0	0.0	0.0	0.1	0.9	0.1	0.6
45	0.84	0.0	0.1	0.0	0.6	0.1	0.0	0.0	0.0	0.0	0.1	0.6	0.0	0.5
46	0.70	0.0	0.1	0.0	0.7	0.1	0.0	0.0	0.0	0.0	0.1	1.1	0.1	0.6
47	0.41	0.0	0.0	0.1	1.9	0.0	0.0	0.0	0.0	0.0	0.2	1.0	0.1	1.3
48	0.53	0.0	0.0	0.0	0.8	0.0	0.0	0.0	0.0	0.0	0.1	0.8	0.0	0.6
49	0.61	0.0	0.1	0.1	1.6	0.0	0.0	0.0	0.0	0.0	0.1	1.4	0.1	1.3
50	0.79	0.1	0.4	0.5	2.5	0.2	0.0	0.0	0.5	0.0	0.7	1.5	0.3	2.2
51	0.74	0.0	0.0	0.1	1.6	0.0	0.0	0.0	0.0	0.0	0.1	0.9	0.1	1.1
52	0.50	0.0	0.1	0.1	1.8	0.1	0.0	0.0	0.0	0.0	0.1	0.9	0.1	1.2
53	0.50	0.1	0.6	0.5	2.7	0.3	0.0	0.0	0.3	0.1	0.8	1.6	0.2	2.4
54	0.27	4.2	2.6	2.0	3.9	6.3	0.8	1.1	3.7	0.2	21.1	2.8	4.7	5.8
55	0.30	0.2	0.8	0.5	2.4	0.2	0.3	0.0	0.8	0.0	0.7	1.3	0.3	2.1
56	0.38	0.0	0.1	0.1	1.5	0.1	0.0	0.0	0.1	0.0	0.1	0.7	0.1	1.1
57	0.39	0.0	0.1	0.0	1.1	0.1	0.0	0.0	0.0	0.0	0.1	0.9	0.1	0.9
58	0.46	0.0	0.0	0.0	1.3	0.1	0.0	0.0	0.0	0.0	0.2	0.9	0.1	0.9
59	0.53	0.0	0.0	0.0	1.5	0.1	0.0	0.0	0.0	0.0	0.2	0.9	0.0	1.0
60	0.30	0.0	0.0	0.0	0.9	0.0	0.0	0.0	0.0	0.0	0.1	0.5	0.0	0.6
61	0.48	0.0	0.0	0.0	0.8	0.0	0.0	0.0	0.0	0.0	0.1	0.5	0.0	0.6
62	0.44	0.0	0.0	0.0	1.1	0.0	0.0	0.0	0.0	0.0	0.1	0.9	0.0	0.8
63	0.41	0.0	0.1	0.2	2.8	0.1	0.0	0.0	0.0	0.0	0.2	1.1	0.1	2.1
64	0.79	0.0	0.1	0.1	0.7	0.1	0.0	0.0	0.0	0.0	0.1	0.7	0.0	0.6
65	0.93	0.0	0.1	0.0	1.0	0.1	0.0	0.0	0.0	0.0	0.1	0.9	0.0	0.7
66	0.67	0.0	0.2	0.1	1.8	0.1	0.0	0.0	0.1	0.1	0.1	1.1	0.1	1.2
67	0.98	0.0	0.1	0.1	1.6	0.1	0.0	0.0	0.0	0.0	0.1	1.0	0.1	1.1
68	1.17	0.0	0.0	0.0	0.5	0.1	0.0	0.0	0.0	0.0	0.0	1.1	0.0	0.5
69	0.92	0.0	0.0	0.0	0.5	0.1	0.0	0.0	0.0	0.0	0.0	0.7	0.0	0.5
70	0.51	0.0	0.0	0.0	1.0	0.1	0.0	0.0	0.0	0.0	0.1	0.7	0.0	0.7
71	0.41	0.0	0.0	0.0	1.2	0.1	0.0	0.0	0.0	0.0	0.1	1.1	0.0	0.8
72	1.21	0.0	0.0	0.0	0.7	0.1	0.0	0.0	0.0	0.0	0.1	0.9	0.0	0.6
73	0.74	0.0	0.0	0.0	0.5	0.2	0.0	0.0	0.0	0.0	0.2	1.5	0.0	0.7
74	1.49	0.0	0.0	0.0	0.6	0.1	0.0	0.0	0.0	0.0	0.0	1.6	0.0	0.7
75	2.47	0.0	0.0	0.0	0.3	0.1	0.0	0.0	0.0	0.0	0.0	2.0	0.0	0.6
76	3.92	0.0	0.0	0.0	0.1	0.2	0.0	0.0	0.0	0.0	0.0	1.1	0.0	0.4
77	0.98	0.0	0.0	0.0	0.3	0.2	0.0	0.0	0.0	0.0	0.0	3.0	0.0	0.6
78	1.14	0.0	0.0	0.0	0.3	0.2	0.0	0.0	0.0	0.0	0.0	1.7	0.0	0.6
79	3.18	0.0	0.0	0.0	0.1	0.6	0.0	0.0	0.0	0.0	0.0	1.2	0.0	0.5
80	1.24	0.0	0.0	0.0	0.3	0.2	0.0	0.0	0.0	0.0	0.0	2.5	0.0	0.7
81	0.98	0.0	0.0	0.0	0.4	0.4	0.0	0.0	0.0	0.0	0.0	1.4	0.0	0.6
82	0.54	0.3	0.1	0.2	3.6	1.0	0.0	0.3	1.8	0.1	1.1	1.4	0.5	1.6
83	1.29	0.0	0.0	0.0	0.1	0.5	0.0	0.0	0.0	0.0	0.0	1.0	0.0	0.3
84	0.86	0.0	0.0	0.0	0.2	0.4	0.0	0.0	0.0	0.0	0.2	1.5	0.0	0.4
85	1.38	0.0	0.0	0.0	0.1	0.5	0.0	0.0	0.0	0.0	0.0	2.1	0.0	0.4
86	1.56	0.0	0.0	0.0	0.1	0.9	0.0	0.0	0.0	0.0	0.0	1.0	0.0	0.3
87	0.63	0.0	0.0	0.0	0.3	0.1	0.0	0.0	0.0	0.0	0.0	1.5	0.0	0.6
88	0.66	0.0	0.0	0.0	0.3	0.1	0.0	0.0	0.0	0.0	0.0	1.4	0.0	0.5
89	0.38	0.0	0.0	0.0	1.3	0.1	0.0	0.0	0.0	0.0	0.1	0.8	0.0	0.8
90	1.09	0.0	0.1	0.0	0.2	0.7	0.0	0.0	0.0	0.0	0.0	1.2	0.0	0.4
91	0.99	0.0	0.0	0.0	0.5	0.4	0.0	0.0	0.0	0.0	0.1	1.1	0.0	0.6
92	1.81	0.0	0.0	0.0	0.1	1.1	0.0	0.0	0.0	0.0	0.0	0.9	0.0	0.4
93	0.67	0.0	0.0	0.0	0.1	1.3	0.0	0.0	0.0	0.0	0.0	0.8	0.0	0.4
94	0.65	0.0	0.0	0.0	0.1	0.4	0.0	0.0	0.0	0.0	0.0	0.5	0.0	0.2
95	1.06	0.0	0.0	0.0	0.5	0.7	0.0	0.0	0.2	0.0	0.2	0.8	0.0	0.4
96	8.86	0.0	0.0	0.0	0.0	0.3	0.0	0.0	0.0	0.0	0.0	0.0	0.0	0.0
97	1.13	0.0	0.0	0.0	0.0	0.1	0.0	0.0	0.0	0.0	0.0	0.4	0.0	0.1
98	0.51	0.0	0.0	0.0	0.1	0.9	0.0	0.0	0.0	0.0	0.0	1.2	0.0	0.4
99	0.97	0.0	0.0	0.0	0.1	0.8	0.0	0.0	0.0	0.0	0.0	1.2	0.0	0.3
100	0.25	1.9	0.7	0.1	0.3	2.9	0.0	28.9	26.1	0.7	3.5	0.5	13.1	1.6
Base	100	1.00	1.00	1.00	1.00	1.00	1.00	1.00	2.13	1.02	1.02	1.02	1.00	1.00

b

Enrichments of top 1% ranked bases non-coding restricted

State	Non coding region %	CADD 1.4	CADD 1.0	Eigen	Eigen-PC	DANN	GERP++	phyloP	PhastCons	REMM	fitCons	FIRE	FATHMM-XF	CDTS	LINSIGHT	FunSeq2
1	0.13	57.8	30.0	50.8	2.6	16.3	56.5	71.7	45.9	53.9	15.2	0.5	80.7	6.8	50.5	6.3
2	0.83	33.3	25.3	35.8	1.0	0.8	36.2	32.9	30.4	27.8	8.3	0.3	47.2	2.1	24.9	3.5
3	0.33	13.3	10.2	15.0	1.3	0.4	14.7	4.2	12.3	10.7	9.3	0.9	20.4	2.7	6.6	3.1
4	0.54	19.2	14.8	18.1	3.1	1.7	19.5	11.9	15.4	16.8	5.9	1.3	17.7	4.1	13.1	5.4
5	1.80	15.8	13.4	14.9	3.1	0.6	17.2	9.3	13.0	15.0	3.9	0.8	6.9	2.8	13.1	5.0
6	3.95	2.5	2.9	1.6	1.8	0.2	1.8	3.7	2.6	3.3	1.5	0.8	0.8	1.6	5.4	2.8
7	0.91	2.5	2.4	3.6	5.5	1.0	3.3	1.8	1.6	3.5	5.0	2.8	2.6	5.5	2.5	3.7
8	0.79	2.0	3.6	1.8	2.2	0.2	2.3	0.8	1.8	1.8	3.5	1.4	0.9	2.3	1.2	2.7
9	0.99	0.4	1.2	0.4	1.6	0.1	0.2	0.0	0.4	0.5	1.3	0.9	0.2	1.5	0.8	1.9
10	0.95	0.2	0.8	0.3	1.7	0.1	0.1	0.0	0.2	0.3	1.5	1.0	0.2	1.6	0.3	1.7
11	0.83	0.2	0.7	0.3	1.4	0.1	0.3	0.6	0.2	0.4	1.2	1.0	0.2	1.2	0.8	1.5
12	0.83	0.3	0.8	0.2	1.1	0.1	0.2	0.8	0.4	0.5	0.8	0.5	0.2	0.9	0.8	1.2
13	1.37	0.5	1.1	0.4	1.2	0.1	0.4	1.2	0.5	0.7	0.8	0.5	0.2	1.1	1.5	1.4
14	1.03	0.4	0.9	0.6	2.3	0.2	0.4	0.9	0.4	0.8	1.5	1.4	0.3	1.9	1.3	2.5
15	0.85	0.2	0.8	0.3	2.0	0.1	0.1	0.0	0.2	0.3	1.5	1.1	0.2	1.8	0.4	1.9
16	0.79	0.0	0.3	0.1	1.1	0.1	0.0	0.0	0.0	0.1	0.7	0.7	0.1	0.9	0.1	1.0
17	2.36	0.2	0.6	0.1	0.9	0.1	0.1	1.0	0.2	0.3	0.7	0.6	0.2	0.8	0.9	1.2
18	1.04	0.0	0.3	0.1	1.2	0.1	0.0	0.0	0.0	0.1	0.8	0.7	0.1	1.0	0.1	1.0
19	1.17	0.6	1.5	0.6	2.0	0.1	0.3	0.0	0.6	0.5	2.0	1.1	0.3	1.8	0.4	2.0
20	1.13	0.7	1.0	1.0	2.3	0.6	0.5	1.1	0.6	1.2	1.1	0.8	0.3	2.1	1.3	1.9
21	0.71	0.3	0.8	0.3	1.4	0.1	0.3	0.7	0.3	0.5	1.2	0.8	0.2	1.2	0.8	1.4
22	0.45	0.1	0.3	0.3	1.9	0.4	0.0	0.2	0.1	0.4	0.9	1.0	0.1	1.7	0.3	1.4
23	0.69	0.0	0.2	0.0	0.9	0.2	0.0	0.0	0.0	0.1	0.6	1.0	0.1	0.8	0.0	0.8
24	0.59	0.0	0.2	0.0	0.6	0.2	0.0	0.0	0.0	0.0	0.6	0.7	0.1	0.6	0.0	0.7
25	0.68	0.0	0.2	0.0	0.6	0.1	0.0	0.0	0.0	0.1	0.5	0.5	0.1	0.6	0.1	0.7
26	0.80	0.1	0.4	0.1	0.7	0.1	0.0	0.2	0.1	0.1	0.6	0.8	0.1	0.7	0.2	0.9
27	1.05	0.0	0.2	0.1	1.1	0.1	0.0	0.0	0.0	0.0	0.9	1.0	0.1	0.9	0.0	1.0
28	0.44	3.1	0.4	6.2	18.6	7.6	0.3	2.9	3.1	5.0	3.7	1.8	2.4	8.0	0.6	5.0
29	1.01	0.0	0.2	0.1	0.7	0.2	0.0	0.0	0.0	0.1	0.4	0.4	0.1	0.5	0.1	0.6
30	1.03	0.0	0.2	0.0	0.3	0.1	0.0	0.0	0.0	0.0	0.3	0.4	0.1	0.4	0.0	0.5
31	0.70	0.0	0.1	0.0	0.6	0.2	0.0	0.0	0.0	0.1	0.4	0.7	0.1	0.7	0.0	0.6
32	1.00	0.0	0.1	0.0	0.5	0.6	0.0	0.0	0.0	0.0	0.4	0.6	0.0	0.6	0.0	0.4
33	0.49	0.0	0.2	0.1	0.6	0.8	0.0	0.0	0.1	0.1	0.4	0.7	0.1	0.5	0.0	0.4
34	0.56	0.1	0.5	0.1	0.7	0.2	0.1	0.4	0.2	0.2	0.6	0.8	0.1	0.6	0.4	0.8
35	1.33	0.1	0.4	0.2	1.6	0.2	0.1	0.2	0.1	0.3	1.2	1.3	0.2	1.4	0.3	1.6
36	1.47	0.0	0.3	0.0	0.5	0.1	0.0	0.0	0.1	0.1	0.5	0.7	0.1	0.6	0.1	0.7
37	2.54	0.2	0.7	0.2	0.9	0.1	0.2	0.8	0.3	0.4	0.9	0.9	0.2	0.9	0.9	1.4
38	1.00	0.1	0.4	0.1	0.6	0.1	0.0	0.2	0.1	0.1	0.6	0.7	0.1	0.7	0.3	0.9
39	0.76	0.0	0.3	0.0	0.5	0.1	0.0	0.1	0.1	0.1	0.5	0.5	0.1	0.5	0.1	0.6
40	0.83	0.0	0.2	0.0	0.3	0.2	0.0	0.0	0.0	0.0	0.3	0.4	0.0	0.4	0.0	0.4
41	0.78	0.0	0.3	0.0	0.7	0.1	0.0	0.1	0.1	0.1	0.4	0.4	0.1	0.6	0.1	0.7
42	0.82	0.0	0.3	0.1	1.2	0.1	0.0	0.1	0.0	0.1	0.6	0.8	0.1	0.9	0.1	1.0
43	1.10	0.0	0.2	0.0	0.7	0.2	0.0	0.0	0.0	0.1	0.5	0.7	0.1	0.7	0.0	0.8
44	1.12	0.0	0.1	0.0	0.7	0.6	0.0	0.0	0.0	0.0	0.5	0.9	0.1	0.7	0.0	0.5
45	0.93	0.0	0.2	0.0	0.6	0.2	0.0	0.0	0.0	0.0	0.5	0.6	0.1	0.6	0.0	0.6
46	0.77	0.0	0.2	0.0	0.6	0.2	0.0	0.0	0.0	0.1	0.7	1.0	0.1	0.7	0.1	0.8
47	0.45	0.0	0.1	0.1	1.7	0.1	0.0	0.0	0.0	0.0	1.2	0.9	0.1	1.4	0.0	1.2
48	0.59	0.0	0.1	0.0	0.7	0.0	0.0	0.0	0.0	0.0	0.6	0.7	0.1	0.7	0.0	0.6
49	0.67	0.0	0.1	0.1	1.5	0.1	0.0	0.0	0.0	0.0	1.1	1.3	0.1	1.3	0.0	1.2
50	0.87	0.2	0.6	0.4	2.3	0.1	0.1	0.0	0.6	0.1	2.1	1.3	0.4	2.2	0.0	2.0
51	0.81	0.0	0.1	0.1	1.5	0.1	0.0	0.0	0.0	0.0	0.9	0.8	0.1	1.2	0.0	1.1
52	0.55	0.0	0.2	0.1	1.7	0.1	0.0	0.0	0.0	0.1	1.0	0.8	0.1	1.3	0.0	1.1
53	0.55	0.3	0.9	0.5	2.5	0.1	0.1	0.0	0.3	0.3	2.4	1.4	0.3	2.4	0.1	2.0
54	0.23	1.2	2.2	1.5	3.4	0.3	1.4	1.3	3.8	0.3	7.5	2.2	7.9	4.0	0.1	2.2
55	0.33	0.4	1.0	0.4	2.3	0.3	0.6	0.0	1.0	0.0	2.0	1.2	0.5	2.1	0.0	2.0
56	0.42	0.0	0.1	0.1	1.4	0.4	0.0	0.0	0.1	0.0	0.8	0.7	0.1	1.1	0.0	1.0
57	0.43	0.0	0.1	0.0	1.1	0.6	0.0	0.0	0.0	0.0	0.7	0.8	0.1	0.9	0.0	0.8
58	0.51	0.0	0.1	0.0	1.2	0.1	0.0	0.0	0.0	0.0	0.8	0.9	0.1	1.0	0.0	0.8
59	0.58	0.0	0.0	0.0	1.4	0.2	0.0	0.0	0.0	0.0	0.7	0.9	0.1	1.0	0.0	0.8
60	0.34	0.0	0.0	0.0	0.8	0.1	0.0	0.0	0.0	0.0	0.4	0.5	0.0	0.6	0.0	0.5
61	0.53	0.0	0.0	0.0	0.7	0.1	0.0	0.0	0.0	0.0	0.5	0.5	0.0	0.6	0.0	0.5
62	0.49	0.0	0.0	0.0	1.0	0.1	0.0	0.0	0.0	0.0	0.7	0.8	0.1	0.8	0.0	0.7
63	0.45	0.0	0.1	0.2	2.6	0.2	0.0	0.0	0.0	0.1	1.3	1.0	0.1	2.1	0.0	1.3
64	0.87	0.0	0.2	0.1	0.7	0.2	0.0	0.0	0.0	0.1	0.5	0.6	0.1	0.6	0.1	0.6
65	1.03	0.0	0.1	0.0	0.9	0.3	0.0	0.0	0.0	0.0	0.6	0.8	0.1	0.8	0.0	0.6
66	0.73	0.1	0.3	0.1	1.6	0.2	0.1	0.0	0.1	0.2	1.1	1.0	0.1	1.3	0.1	1.2
67	1.08	0.0	0.1	0.1	1.4	0.3	0.0	0.0	0.0	0.1	0.8	0.9	0.1	1.1	0.1	0.8
68	1.30	0.0	0.1	0.0	0.4	0.3	0.0	0.0	0.0	0.0	0.4	1.0	0.0	0.6	0.0	0.3
69	1.01	0.0	0.1	0.0	0.4	0.6	0.0	0.0	0.0	0.0	0.3	0.6	0.0	0.5	0.0	0.3
70	0.55	0.0	0.0	0.0	0.9	0.3	0.0	0.0	0.0	0.0	0.4	0.7	0.0	0.7	0.0	0.5
71	0.45	0.0	0.0	0.0	1.1	0.2	0.0	0.0	0.0	0.0	0.6	1.0	0.0	0.8	0.0	0.6
72	1.31	0.0	0.1	0.0	0.6	0.6	0.0	0.0	0.0	0.0	0.4	0.9	0.1	0.6	0.0	0.4
73	0.81	0.0	0.0	0.0	0.5	0.6	0.0	0.0	0.0	0.0	0.6	1.4	0.1	0.7	0.0	0.4
74	1.64	0.0	0.1	0.0	0.6	0.3	0.0	0.0	0.0	0.0	0.6	1.5	0.1	0.7	0.0	0.5
75	2.73	0.0	0.0	0.0	0.3	0.7	0.0	0.0	0.0	0.0	0.4	1.8	0.0	0.6	0.0	0.3
76	4.33	0.0	0.1	0.0	0.1	1.5	0.0	0.0	0.0	0.0	0.3	1.0	0.0	0.4	0.0	0.2
77	1.08	0.0	0.0	0.0	0.2	1.0	0.0	0.0	0.0	0.0	0.4	2.8	0.0	0.7	0.0	0.2
78	1.26	0.0	0.1	0.0	0.3	1.0	0.0	0.0	0.0	0.0	0.3	1.6	0.0	0.6	0.0	0.3
79	3.51	0.0	0.0	0.0	0.1	3.0	0.0	0.0	0.0	0.0	0.2	1.1	0.0	0.5	0.0	0.2
80	1.37	0.0	0.0	0.0	0.3	1.0	0.0	0.0	0.0	0.0	0.3	2.3	0.0	0.8	0.0	0.3
81	1.08	0.0	0.0	0.0	0.3	2.0	0.0	0.0	0.0	0.0	0.3	1.3	0.0	0.7	0.0	0.3
82	0.57	0.1	0.1	0.2	3.4	1.4	0.0	0.8	2.2	0.2	3.0	1.3	0.7	1.6	0.0	0.8
83	1.38	0.0	0.1	0.0	0.1	2.6	0.0	0.0	0.0	0.0	0.3	1.0	0.0	0.3	0.0	0.1
84	0.91	0.0	0.0	0.0	0.2	1.6	0.0	0.0	0.0	0.0	0.5	1.5	0.0	0.4	0.0	0.2
85	1.46	0.0	0.0	0.0	0.1	2.6	0.0	0.0	0.0	0.0	0.3	2.1	0.0	0.5	0.0	0.2
86	1.73	0.0	0.0	0.0	0.0	3.9	0.0	0.0	0.0	0.0	0.2	1.0	0.0	0.3	0.0	0.1
87	0.69	0.0	0.0	0.0	0.2	0.4	0.0	0.0	0.0	0.0	0.3	1.4	0.0	0.6	0.0	0.3
88	0.73	0.0	0.0	0.0	0.3	0.3	0.0	0.0	0.0	0.0	0.4	1.3	0.0	0.6	0.0	0.3
89	0.41	0.0	0.0	0.0	1.2	0.6	0.0	0.0	0.0	0.0	0.6	0.7	0.1	0.8	0.0	0.6
90	1.19	0.0	0.2	0.0	0.2	3.6	0.0	0.0	0.0	0.0	0.3	1.1	0.0	0.5	0.0	0.2
91	1.07	0.0	0.1	0.0	0.5	1.7	0.0	0.0	0.0	0.0	0.5	1.1	0.1	0.7	0.0	0.3
92	2.00	0.0	0.0	0.0	0.1	4.9	0.0	0.0	0.0	0.0	0.2	0.8	0.0	0.4	0.0	0.1
93	0.74	0.0	0.0	0.0	0.1	5.8	0.0	0.0	0.0	0.0	0.2	0.7	0.0	0.4	0.0	0.2
94	0.72	0.0	0.0	0.0	0.1	1.7	0.0	0.0	0.0	0.0	0.3	0.5	0.0	0.2	0.0	0.1
95	1.08	0.0	0.0	0.0	0.4	2.0	0.0	0.0	0.1	0.0	0.9	0.8	0.1	0.5	0.0	0.2
96	1.33	0.0	0.0	0.1	0.2	4.2	0.0	0.0	0.0	0.0	0.5	0.3	0.4	0.2	0.0	0.1
97	0.96	0.0	0.0	0.0	0.0	0.4	0.0	0.0	0.0	0.0	0.5	0.5	0.0	0.1	0.0	0.1
98	0.56	0.0	0.0	0.0	0.1	4.1	0.0	0.0	0.0	0.0	0.4	1.1	0.0	0.4	0.0	0.1
99	1.05	0.0	0.0	0.0	0.0	3.5	0.0	0.0	0.0	0.0	0.2	1.1	0.0	0.3	0.0	0.1
100	0.26	0.7	0.6	0.1	0.3	2.0	0.0	34.5	32.8	1.3	14.7	0.5	17.9	1.6	0.0	0.3
Base	90.44	0.99	0.99	0.99	0.99	0.99	1.00	0.99	1.71	1.01	1.01	1.01	0.99	0.99	0.99	0.99

Supplementary Figure 25: Enrichment of all conservation states for top 1% of bases prioritized by variant prioritization scores. The figure displays for top 1% prioritized bases **(a)** genome-wide and **(b)** in non-coding regions by variant prioritization scores the same enrichments as shown in **Fig. 6a,b** except here enrichments for all conservation states are shown and also included is the original version of the CADD score in addition to v1.4. Coloring of enrichments is based on their value in a column specific manner. The second columns gives the percentage of the background region used to compute the enrichments falling in each state, which is for **(a)** the whole genome and for **(b)** bases scored by both LINSIGHT and FunSeq2. The last line in both heatmaps gives the actual percentage of the background set covered by each set of prioritized bases, which can differ from 1.00% because of how ties were handled.

a

Enrichments of top 5% ranked bases genome-wide

State	Genome %	CADD 1.4	CADD 1.0	Eigen	Eigen-PC	DANN	GERP++	phyloP	PhastCons	REMM	fitCons	FIRE	FATHMM-XF	CDTS
1	0.60	18.5	15.7	13.4	10.4	15.9	16.5	16.8	18.3	17.3	16.6	2.2	17.2	6.2
2	0.84	13.7	10.6	14.2	2.7	2.9	12.7	12.0	14.2	12.0	5.0	0.7	16.9	2.3
3	0.46	10.7	7.0	10.5	2.5	4.5	7.6	4.3	8.9	8.8	9.0	1.9	12.3	3.5
4	0.60	11.3	8.5	10.9	3.5	4.0	9.9	7.4	10.2	9.6	6.0	1.7	11.7	3.0
5	1.66	9.4	7.1	11.1	2.7	1.1	9.8	6.5	8.8	8.5	2.7	0.9	8.6	2.0
6	3.59	2.8	2.6	3.6	1.8	0.6	4.5	3.2	2.8	3.2	1.4	0.8	2.0	1.4
7	0.85	2.8	2.7	4.1	3.1	1.3	3.8	2.2	1.9	3.3	3.3	2.5	5.1	3.0
8	0.73	3.3	3.2	4.4	2.1	0.6	3.3	1.2	2.4	3.0	2.4	1.2	3.3	1.8
9	0.90	1.2	1.6	1.5	1.7	0.4	1.4	0.4	0.9	1.6	1.3	0.9	0.7	1.4
10	0.86	0.8	1.3	1.1	1.7	0.3	0.7	0.2	0.5	1.1	1.4	1.0	0.7	1.4
11	0.75	0.7	1.1	0.9	1.5	0.5	1.5	1.1	0.6	1.2	1.1	1.0	0.7	1.2
12	0.75	0.8	1.2	0.9	1.2	0.5	1.6	1.3	0.8	1.2	0.9	0.6	0.8	1.0
13	1.24	1.0	1.4	1.3	1.3	0.5	2.2	1.6	1.0	1.4	1.0	0.7	0.8	1.1
14	0.94	1.0	1.3	1.5	2.0	0.5	2.0	1.4	0.9	1.8	1.5	1.3	0.8	1.6
15	0.77	0.7	1.3	1.2	1.9	0.3	0.8	0.2	0.5	1.2	1.4	1.0	0.6	1.5
16	0.72	0.2	0.6	0.3	1.2	0.3	0.4	0.1	0.2	0.6	0.9	0.8	0.2	1.1
17	2.14	0.6	1.0	0.8	1.1	0.5	1.9	1.8	0.7	1.1	0.8	0.7	0.8	1.0
18	0.94	0.2	0.6	0.3	1.3	0.3	0.2	0.1	0.1	0.5	0.9	0.8	0.2	1.1
19	1.07	1.5	1.9	2.0	1.9	0.4	1.1	0.3	1.0	1.6	1.7	1.0	1.3	1.6
20	1.03	1.0	1.3	1.4	1.6	0.6	2.0	1.4	0.9	1.6	1.2	0.9	0.9	1.4
21	0.64	0.7	1.1	1.0	1.4	0.5	1.5	1.1	0.7	1.2	1.1	0.9	0.8	1.2
22	0.41	0.4	0.7	0.6	1.6	0.6	0.8	1.0	0.4	1.0	1.1	1.1	0.5	1.3
23	0.63	0.3	0.5	0.2	1.0	0.7	0.1	1.5	0.4	0.6	0.8	1.2	0.4	1.0
24	0.53	0.2	0.5	0.2	0.8	0.6	0.2	1.3	0.3	0.5	0.7	0.9	0.4	0.9
25	0.61	0.2	0.6	0.2	0.8	0.5	0.3	1.3	0.3	0.5	0.6	0.7	0.4	0.9
26	0.72	0.4	0.7	0.4	0.9	0.5	0.8	1.1	0.4	0.7	0.7	1.0	0.5	1.0
27	0.96	0.2	0.6	0.3	1.2	0.3	0.1	0.0	0.2	0.5	0.9	1.1	0.2	1.1
28	0.44	3.2	1.7	4.3	7.1	4.0	1.3	2.4	3.1	4.4	4.5	2.0	2.7	2.7
29	0.92	0.2	0.6	0.2	0.9	0.5	0.4	1.1	0.3	0.5	0.6	0.5	0.4	0.8
30	0.94	0.2	0.5	0.1	0.6	0.5	0.2	1.5	0.3	0.4	0.5	0.7	0.4	0.8
31	0.63	0.2	0.5	0.2	0.8	0.6	0.1	1.4	0.3	0.5	0.7	0.9	0.3	0.9
32	0.93	0.3	0.4	0.1	0.7	1.0	0.0	0.5	0.4	0.3	0.5	0.9	0.2	0.9
33	0.45	0.4	0.7	0.1	0.6	1.3	0.0	1.0	0.4	0.4	0.5	0.9	0.3	0.7
34	0.51	0.6	1.1	0.5	0.8	0.8	0.8	1.4	0.6	0.7	0.7	1.0	0.6	0.9
35	1.20	0.5	0.8	0.7	1.5	0.6	0.9	1.6	0.6	1.1	1.2	1.4	0.7	1.3
36	1.34	0.3	0.6	0.3	0.8	0.6	0.4	1.7	0.5	0.6	0.7	0.9	0.5	0.9
37	2.30	0.8	1.1	0.9	1.1	0.5	1.7	1.5	0.8	1.2	1.0	1.0	0.7	1.1
38	0.91	0.4	0.8	0.4	0.8	0.5	1.0	1.4	0.5	0.7	0.7	0.9	0.6	1.0
39	0.70	0.3	0.7	0.3	0.7	0.5	0.5	1.3	0.4	0.6	0.6	0.8	0.5	0.8
40	0.76	0.2	0.5	0.1	0.6	0.6	0.1	1.6	0.3	0.4	0.5	0.7	0.4	0.8
41	0.70	0.3	0.7	0.3	0.9	0.5	0.6	1.1	0.4	0.6	0.6	0.5	0.5	0.9
42	0.74	0.3	0.6	0.3	1.2	0.5	0.5	1.2	0.3	0.8	0.8	0.9	0.4	1.1
43	1.00	0.3	0.5	0.2	0.9	0.6	0.1	1.6	0.4	0.6	0.7	1.0	0.4	1.0
44	1.02	0.3	0.5	0.1	0.9	1.0	0.0	1.1	0.5	0.4	0.7	1.2	0.4	0.9
45	0.84	0.2	0.5	0.2	0.8	0.6	0.1	1.3	0.3	0.5	0.6	0.9	0.4	0.9
46	0.70	0.3	0.6	0.2	0.9	0.6	0.3	1.4	0.4	0.6	0.8	1.2	0.4	1.0
47	0.41	0.0	0.3	0.2	1.6	0.2	0.0	0.0	0.0	0.2	1.2	1.0	0.2	1.2
48	0.53	0.1	0.3	0.1	0.9	0.2	0.0	0.0	0.1	0.1	0.7	0.9	0.2	0.9
49	0.61	0.1	0.3	0.3	1.4	0.2	0.1	0.0	0.2	0.3	1.1	1.3	0.3	1.3
50	0.79	0.8	0.9	1.5	2.0	0.2	0.6	0.3	1.0	0.3	1.8	1.1	1.3	1.7
51	0.74	0.1	0.3	0.3	1.4	0.2	0.1	0.1	0.2	0.2	1.0	0.8	0.3	1.2
52	0.50	0.1	0.5	0.4	1.6	0.3	0.1	0.0	0.1	0.5	1.1	0.9	0.2	1.2
53	0.50	0.9	1.4	1.4	2.2	0.4	0.5	0.2	0.6	1.2	1.9	1.2	1.2	1.8
54	0.27	3.9	2.7	4.4	2.8	2.1	1.6	1.5	3.8	1.2	7.0	2.2	7.6	3.3
55	0.30	1.0	1.3	1.5	2.0	0.5	1.0	0.4	1.3	0.1	1.7	1.1	1.4	1.6
56	0.38	0.1	0.4	0.2	1.3	0.5	0.2	0.1	0.2	0.0	0.9	0.7	0.3	1.1
57	0.39	0.0	0.2	0.1	1.1	0.5	0.1	0.0	0.1	0.0	0.8	0.9	0.2	1.0
58	0.46	0.1	0.3	0.2	1.2	0.3	0.0	0.0	0.1	0.2	0.9	1.0	0.2	1.1
59	0.53	0.0	0.2	0.1	1.4	0.4	0.0	0.0	0.1	0.2	0.9	1.1	0.1	1.0
60	0.30	0.0	0.2	0.1	1.0	0.2	0.0	0.0	0.1	0.1	0.6	0.6	0.1	0.9
61	0.48	0.0	0.2	0.1	0.9	0.2	0.0	0.0	0.1	0.1	0.7	0.7	0.1	0.8
62	0.44	0.0	0.2	0.1	1.1	0.3	0.0	0.0	0.1	0.2	0.8	1.0	0.1	1.0
63	0.41	0.2	0.4	0.5	1.9	0.3	0.1	0.1	0.2	0.4	1.3	1.0	0.3	1.4
64	0.79	0.3	0.6	0.2	0.8	0.6	0.3	1.4	0.3	0.5	0.6	0.8	0.3	0.9
65	0.93	0.2	0.4	0.1	1.0	0.6	0.0	0.4	0.2	0.4	0.7	1.0	0.2	0.9
66	0.67	0.3	0.6	0.5	1.5	0.5	0.3	1.1	0.3	0.8	1.1	1.0	0.4	1.2
67	0.98	0.2	0.4	0.3	1.3	0.6	0.1	0.6	0.2	0.5	0.8	1.0	0.3	1.1
68	1.17	0.2	0.3	0.1	0.6	0.7	0.0	0.1	0.1	0.2	0.5	1.1	0.1	0.9
69	0.92	0.2	0.4	0.1	0.7	1.0	0.0	0.4	0.3	0.3	0.5	0.9	0.2	0.8
70	0.51	0.1	0.2	0.1	1.0	0.5	0.0	0.0	0.1	0.1	0.6	0.9	0.1	0.9
71	0.41	0.1	0.2	0.1	1.1	0.4	0.0	0.0	0.1	0.1	0.7	1.1	0.1	0.9
72	1.21	0.3	0.4	0.1	0.8	0.9	0.0	0.3	0.3	0.3	0.6	1.1	0.2	0.9
73	0.74	0.2	0.3	0.1	0.6	0.9	0.0	0.1	0.2	0.2	0.6	1.5	0.1	1.0
74	1.49	0.2	0.3	0.1	0.7	0.6	0.0	0.0	0.2	0.3	0.6	1.5	0.1	1.0
75	2.47	0.2	0.3	0.0	0.5	0.9	0.0	0.0	0.2	0.1	0.5	1.7	0.1	1.0
76	3.92	0.5	0.5	0.0	0.3	1.2	0.0	0.0	0.4	0.1	0.5	1.1	0.0	0.8
77	0.98	0.3	0.3	0.0	0.3	1.0	0.0	0.0	0.2	0.1	0.5	2.4	0.1	1.0
78	1.14	0.4	0.4	0.0	0.4	1.0	0.0	0.0	0.3	0.1	0.5	1.5	0.1	0.9
79	3.18	0.4	0.4	0.0	0.3	2.0	0.0	0.0	0.4	0.0	0.4	1.2	0.0	0.8
80	1.24	0.2	0.2	0.0	0.4	1.1	0.0	0.0	0.2	0.1	0.4	2.1	0.1	1.0
81	0.98	0.3	0.3	0.0	0.5	1.6	0.0	0.0	0.5	0.1	0.4	1.4	0.1	0.9
82	0.54	0.7	0.4	0.5	2.7	1.2	0.0	1.9	3.3	0.6	2.0	1.5	1.4	0.9
83	1.29	0.6	0.5	0.0	0.2	1.7	0.0	0.0	0.3	0.0	0.4	1.0	0.0	0.5
84	0.86	0.3	0.3	0.0	0.3	1.4	0.0	0.0	0.3	0.1	0.5	1.4	0.1	0.6
85	1.38	0.3	0.2	0.0	0.2	1.7	0.0	0.0	0.2	0.0	0.4	1.8	0.0	0.7
86	1.56	0.4	0.3	0.0	0.1	2.4	0.0	0.0	0.3	0.0	0.2	1.0	0.0	0.5
87	0.63	0.0	0.0	0.0	0.3	0.4	0.0	0.0	0.1	0.0	0.4	1.4	0.0	0.8
88	0.66	0.0	0.0	0.0	0.4	0.3	0.0	0.0	0.1	0.0	0.5	1.3	0.1	0.9
89	0.38	0.0	0.1	0.1	1.1	0.6	0.0	0.0	0.1	0.0	0.7	0.9	0.1	0.9
90	1.09	1.0	1.0	0.0	0.3	2.3	0.0	0.0	0.3	0.1	0.4	1.2	0.0	0.7
91	0.99	0.3	0.3	0.1	0.5	1.6	0.0	0.1	0.3	0.1	0.5	1.2	0.1	0.8
92	1.81	0.2	0.2	0.0	0.2	3.0	0.0	0.0	0.2	0.0	0.3	0.9	0.0	0.6
93	0.67	0.2	0.2	0.0	0.2	3.5	0.0	0.0	0.1	0.0	0.3	0.8	0.0	0.5
94	0.65	0.0	0.0	0.0	0.1	1.3	0.0	0.0	0.0	0.0	0.3	0.6	0.0	0.3
95	1.06	0.1	0.1	0.1	0.6	1.1	0.0	0.1	1.4	0.1	0.6	0.8	0.2	0.4
96	8.86	0.0	0.0	0.0	0.0	0.2	0.0	0.0	0.0	0.0	0.0	0.0	0.3	0.0
97	1.13	0.0	0.0	0.0	0.1	0.2	0.0	0.0	0.0	0.0	0.3	0.4	0.0	0.2
98	0.51	0.2	0.1	0.0	0.1	2.5	0.0	0.0	0.0	0.0	0.4	1.1	0.0	0.5
99	0.97	0.1	0.1	0.0	0.1	2.2	0.0	0.0	0.1	0.0	0.3	1.1	0.0	0.5
100	0.25	3.6	1.4	1.9	0.9	2.1	0.1	10.7	15.6	2.3	4.9	1.3	10.7	1.1
Base	100	5.00	5.00	4.98	5.00	5.00	5.02	5.00	5.00	5.03	5.03	5.01	5.00	5.00

b

Enrichments of top 5% ranked bases non-coding restricted

State	Non coding region %	CADD 1.4	CADD 1.0	Eigen	Eigen-PC	DANN	GERP++	phyloP	PhastCons	REMM	fitCons	FIRE	FATHMM-XF	CDTS	LINSIGHT	FunSeq2
1	0.13	16.9	12.2	17.3	5.3	6.6	15.2	17.1	17.7	15.9	6.4	0.8	19.0	4.0	17.9	6.6
2	0.83	13.6	10.5	14.9	2.2	1.4	12.7	11.9	14.3	11.9	3.6	0.5	17.6	2.0	14.6	4.4
3	0.33	9.5	6.8	11.1	2.0	0.6	7.8	4.2	8.4	7.7	4.0	0.9	15.0	2.2	11.4	3.8
4	0.54	10.4	7.9	11.7	2.9	1.4	9.5	6.9	9.6	8.9	3.4	1.1	12.3	2.4	11.7	4.4
5	1.80	9.6	7.2	11.3	2.7	0.9	9.9	6.6	9.0	8.6	2.6	0.8	8.8	1.8	10.8	4.1
6	3.95	3.0	2.7	3.6	1.8	0.7	4.7	3.3	2.9	3.4	1.5	0.8	2.1	1.4	3.9	2.8
7	0.91	2.8	2.7	4.2	3.1	1.1	3.8	2.2	2.0	3.3	3.3	2.2	5.3	2.8	4.2	3.4
8	0.79	3.5	3.3	4.4	2.1	0.5	3.4	1.3	2.5	3.1	2.4	1.1	3.5	1.7	4.5	2.5
9	0.99	1.3	1.7	1.6	1.7	0.4	1.5	0.4	0.9	1.7	1.5	0.8	0.7	1.3	1.7	1.8
10	0.95	0.9	1.3	1.2	1.7	0.4	0.8	0.2	0.6	1.2	1.5	0.9	0.8	1.4	1.4	1.5
11	0.83	0.7	1.1	0.9	1.5	0.5	1.6	1.2	0.6	1.3	1.3	0.9	0.7	1.2	1.0	1.6
12	0.83	0.9	1.2	1.0	1.2	0.6	1.7	1.4	0.8	1.3	1.0	0.6	0.8	1.0	1.1	1.4
13	1.37	1.1	1.4	1.3	1.2	0.5	2.3	1.7	1.0	1.5	1.1	0.6	0.9	1.1	1.5	1.7
14	1.03	1.1	1.3	1.5	2.0	0.6	2.1	1.5	0.9	1.9	1.7	1.3	0.8	1.5	1.4	2.3
15	0.85	0.9	1.3	1.2	1.9	0.4	0.8	0.2	0.5	1.3	1.6	0.9	0.6	1.4	1.3	1.7
16	0.79	0.2	0.7	0.3	1.2	0.4	0.4	0.1	0.2	0.6	1.0	0.7	0.3	1.0	0.3	1.0
17	2.36	0.7	1.1	0.8	1.1	0.6	2.0	1.9	0.8	1.2	0.9	0.7	0.8	1.0	0.9	1.6
18	1.04	0.2	0.7	0.3	1.3	0.3	0.2	0.1	0.2	0.6	1.1	0.7	0.3	1.1	0.4	1.0
19	1.17	1.6	1.9	2.0	1.9	0.4	1.1	0.4	1.1	1.8	1.8	0.9	1.3	1.5	2.4	1.8
20	1.13	1.1	1.4	1.5	1.6	0.7	2.1	1.4	1.0	1.6	1.3	0.8	0.9	1.3	1.5	1.8
21	0.71	0.8	1.2	1.0	1.4	0.5	1.6	1.2	0.7	1.3	1.3	0.8	0.8	1.1	1.1	1.6
22	0.45	0.5	0.8	0.6	1.5	0.7	0.8	1.0	0.4	1.1	1.2	1.0	0.5	1.3	0.4	1.4
23	0.69	0.3	0.5	0.2	1.0	0.7	0.2	1.6	0.4	0.6	0.9	1.1	0.4	1.0	0.1	0.9
24	0.59	0.3	0.5	0.2	0.8	0.6	0.2	1.4	0.3	0.6	0.8	0.8	0.4	0.8	0.1	0.8
25	0.68	0.3	0.6	0.2	0.8	0.6	0.4	1.3	0.3	0.6	0.7	0.7	0.4	0.8	0.1	0.9
26	0.80	0.4	0.8	0.4	0.8	0.6	0.9	1.2	0.5	0.7	0.8	0.9	0.5	0.9	0.3	1.2
27	1.05	0.2	0.6	0.3	1.2	0.4	0.2	0.1	0.2	0.5	1.1	1.0	0.3	1.1	0.2	0.9
28	0.44	2.5	1.3	4.4	7.5	3.3	1.2	2.2	2.8	4.1	4.5	1.8	2.7	2.7	2.9	3.2
29	1.01	0.3	0.6	0.2	0.9	0.6	0.4	1.2	0.3	0.6	0.7	0.5	0.4	0.8	0.2	0.8
30	1.03	0.3	0.6	0.1	0.6	0.6	0.2	1.5	0.3	0.5	0.6	0.6	0.4	0.7	0.1	0.7
31	0.70	0.3	0.5	0.2	0.8	0.7	0.1	1.5	0.3	0.6	0.8	0.9	0.4	0.9	0.1	0.7
32	1.00	0.3	0.5	0.1	0.7	1.1	0.0	0.6	0.5	0.3	0.6	0.8	0.2	0.8	0.0	0.4
33	0.49	0.5	0.8	0.1	0.6	1.5	0.0	1.1	0.5	0.4	0.6	0.9	0.3	0.7	0.1	0.4
34	0.56	0.7	1.2	0.5	0.8	0.9	0.9	1.4	0.6	0.8	0.8	0.9	0.6	0.9	0.5	1.0
35	1.33	0.6	0.9	0.7	1.5	0.7	0.9	1.6	0.7	1.2	1.4	1.3	0.7	1.3	0.5	1.7
36	1.47	0.4	0.7	0.3	0.7	0.6	0.4	1.7	0.5	0.7	0.7	0.9	0.6	0.9	0.1	1.0
37	2.54	0.9	1.2	0.9	1.1	0.6	1.8	1.6	0.9	1.3	1.1	1.0	0.8	1.1	0.9	1.7
38	1.00	0.5	0.9	0.4	0.8	0.6	1.1	1.4	0.6	0.8	0.8	0.9	0.6	1.0	0.4	1.2
39	0.76	0.4	0.7	0.3	0.7	0.6	0.5	1.3	0.5	0.6	0.7	0.7	0.5	0.8	0.2	0.9
40	0.83	0.3	0.5	0.1	0.6	0.7	0.1	1.7	0.4	0.4	0.6	0.7	0.4	0.8	0.0	0.6
41	0.78	0.4	0.7	0.3	0.9	0.6	0.6	1.2	0.4	0.7	0.7	0.5	0.5	0.8	0.3	0.9
42	0.82	0.4	0.7	0.4	1.2	0.6	0.5	1.2	0.4	0.8	1.0	0.9	0.5	1.0	0.2	1.1
43	1.10	0.3	0.6	0.2	0.9	0.7	0.2	1.7	0.4	0.7	0.8	0.9	0.4	1.0	0.1	0.8
44	1.12	0.4	0.5	0.1	0.9	1.1	0.0	1.2	0.5	0.4	0.8	1.1	0.4	0.9	0.0	0.5
45	0.93	0.3	0.6	0.2	0.8	0.7	0.2	1.4	0.3	0.5	0.7	0.8	0.4	0.9	0.1	0.7
46	0.77	0.3	0.6	0.2	0.9	0.7	0.4	1.5	0.4	0.7	0.9	1.1	0.5	0.9	0.1	1.0
47	0.45	0.1	0.3	0.2	1.6	0.2	0.0	0.0	0.0	0.3	1.3	0.9	0.3	1.2	0.2	1.0
48	0.59	0.1	0.3	0.1	0.9	0.3	0.0	0.0	0.1	0.2	0.8	0.8	0.2	0.9	0.1	0.6
49	0.67	0.1	0.4	0.3	1.4	0.2	0.1	0.1	0.2	0.3	1.3	1.2	0.3	1.2	0.2	1.0
50	0.87	0.9	0.9	1.5	2.0	0.2	0.7	0.3	1.1	0.4	1.9	1.0	1.3	1.6	1.8	1.6
51	0.81	0.1	0.3	0.3	1.4	0.2	0.1	0.1	0.2	0.2	1.1	0.8	0.3	1.1	0.2	0.9
52	0.55	0.2	0.5	0.4	1.5	0.3	0.1	0.1	0.1	0.5	1.2	0.8	0.3	1.2	0.3	1.0
53	0.55	1.0	1.4	1.5	2.2	0.3	0.5	0.2	0.6	1.3	2.0	1.1	1.2	1.7	1.8	1.6
54	0.23	2.7	2.2	3.8	2.6	0.4	1.6	1.3	3.3	1.0	3.7	1.6	8.8	2.5	4.8	2.1
55	0.33	1.0	1.3	1.5	1.9	0.4	1.1	0.4	1.3	0.1	1.8	1.0	1.5	1.5	1.9	1.7
56	0.42	0.1	0.4	0.2	1.3	0.5	0.2	0.1	0.2	0.0	1.1	0.7	0.3	1.0	0.2	0.9
57	0.43	0.0	0.3	0.1	1.1	0.6	0.1	0.0	0.2	0.0	1.0	0.9	0.3	1.0	0.1	0.7
58	0.51	0.1	0.3	0.2	1.2	0.3	0.0	0.0	0.1	0.2	1.0	0.9	0.2	1.0	0.1	0.7
59	0.58	0.1	0.2	0.1	1.3	0.5	0.0	0.0	0.1	0.2	1.0	1.0	0.1	1.0	0.0	0.6
60	0.34	0.0	0.2	0.1	1.0	0.3	0.0	0.0	0.1	0.1	0.7	0.6	0.1	0.8	0.0	0.5
61	0.53	0.0	0.2	0.1	0.9	0.3	0.0	0.0	0.1	0.2	0.7	0.6	0.1	0.8	0.0	0.5
62	0.49	0.1	0.2	0.1	1.1	0.3	0.0	0.0	0.1	0.2	0.9	0.9	0.1	0.9	0.0	0.7
63	0.45	0.2	0.4	0.5	1.8	0.3	0.1	0.1	0.2	0.4	1.5	0.9	0.3	1.3	0.4	1.1
64	0.87	0.3	0.6	0.2	0.8	0.7	0.3	1.4	0.3	0.6	0.7	0.8	0.4	0.9	0.1	0.8
65	1.03	0.2	0.4	0.2	1.0	0.7	0.0	0.5	0.2	0.5	0.8	1.0	0.2	0.9	0.1	0.6
66	0.73	0.4	0.6	0.5	1.5	0.6	0.4	1.1	0.3	0.9	1.2	1.0	0.4	1.1	0.4	1.2
67	1.08	0.2	0.4	0.3	1.3	0.6	0.1	0.7	0.2	0.5	1.0	0.9	0.3	1.0	0.2	0.7
68	1.30	0.2	0.3	0.1	0.6	0.7	0.0	0.1	0.2	0.3	0.6	1.0	0.2	0.8	0.0	0.4
69	1.01	0.3	0.4	0.1	0.7	1.1	0.0	0.4	0.4	0.3	0.6	0.8	0.2	0.8	0.0	0.3
70	0.55	0.1	0.2	0.1	1.0	0.6	0.0	0.0	0.1	0.1	0.7	0.8	0.1	0.9	0.0	0.4
71	0.45	0.1	0.2	0.1	1.1	0.5	0.0	0.0	0.1	0.2	0.8	1.0	0.1	0.9	0.0	0.5
72	1.31	0.3	0.4	0.1	0.8	1.1	0.0	0.4	0.4	0.3	0.7	1.1	0.2	0.9	0.0	0.4
73	0.81	0.2	0.3	0.1	0.6	1.0	0.0	0.1	0.3	0.2	0.7	1.4	0.1	0.9	0.0	0.4
74	1.64	0.3	0.3	0.1	0.7	0.7	0.0	0.0	0.2	0.3	0.8	1.4	0.1	1.0	0.1	0.5
75	2.73	0.3	0.4	0.0	0.5	1.0	0.0	0.0	0.3	0.2	0.6	1.6	0.1	1.0	0.0	0.4
76	4.33	0.6	0.5	0.0	0.3	1.3	0.0	0.0	0.5	0.1	0.5	1.0	0.1	0.8	0.0	0.3
77	1.08	0.4	0.3	0.0	0.3	1.1	0.0	0.0	0.3	0.1	0.6	2.2	0.1	1.0	0.0	0.3
78	1.26	0.5	0.5	0.0	0.4	1.1	0.0	0.0	0.4	0.1	0.5	1.4	0.1	0.9	0.0	0.3
79	3.51	0.6	0.4	0.0	0.3	2.1	0.0	0.0	0.5	0.0	0.5	1.1	0.0	0.7	0.0	0.2
80	1.37	0.2	0.2	0.0	0.4	1.2	0.0	0.0	0.3	0.1	0.5	1.9	0.1	1.0	0.0	0.3
81	1.08	0.4	0.3	0.0	0.5	1.7	0.0	0.0	0.6	0.1	0.5	1.3	0.1	0.9	0.0	0.2
82	0.57	0.7	0.4	0.5	2.8	1.2	0.0	1.9	3.3	0.7	2.2	1.4	1.5	0.9	0.7	0.7
83	1.38	0.7	0.6	0.0	0.2	1.8	0.0	0.0	0.4	0.0	0.4	0.9	0.0	0.5	0.0	0.1
84	0.91	0.4	0.3	0.0	0.3	1.6	0.0	0.0	0.4	0.1	0.6	1.3	0.1	0.6	0.0	0.2
85	1.46	0.4	0.3	0.0	0.2	1.9	0.0	0.0	0.4	0.0	0.5	1.7	0.0	0.7	0.0	0.1
86	1.73	0.5	0.3	0.0	0.1	2.5	0.0	0.0	0.5	0.0	0.3	1.0	0.0	0.5	0.0	0.1
87	0.69	0.0	0.0	0.0	0.3	0.4	0.0	0.0	0.1	0.0	0.5	1.3	0.0	0.8	0.0	0.2
88	0.73	0.0	0.0	0.0	0.4	0.3	0.0	0.0	0.1	0.0	0.6	1.2	0.1	0.9	0.0	0.3
89	0.41	0.0	0.1	0.1	1.1	0.6	0.0	0.0	0.1	0.0	0.9	0.8	0.1	0.9	0.0	0.5
90	1.19	1.2	1.0	0.0	0.3	2.4	0.0	0.0	0.4	0.1	0.4	1.1	0.1	0.7	0.1	0.2
91	1.07	0.3	0.4	0.1	0.5	1.8	0.0	0.1	0.3	0.1	0.6	1.2	0.1	0.8	0.0	0.2
92	2.00	0.3	0.2	0.0	0.2	3.1	0.0	0.0	0.3	0.0	0.3	0.9	0.0	0.6	0.0	0.1
93	0.74	0.3	0.2	0.0	0.2	3.6	0.0	0.0	0.2	0.0	0.4	0.8	0.0	0.5	0.0	0.1
94	0.72	0.0	0.0	0.0	0.1	1.4	0.0	0.0	0.0	0.0	0.4	0.6	0.0	0.3	0.0	0.1
95	1.08	0.2	0.1	0.1	0.6	1.2	0.0	0.1	1.4	0.1	0.7	0.8	0.2	0.4	0.1	0.2
96	1.33	0.0	0.0	0.0	0.2	1.4	0.0	0.0	0.0	0.0	0.4	0.4	2.1	0.1	0.0	0.0
97	0.96	0.0	0.0	0.0	0.1	0.3	0.0	0.0	0.0	0.0	0.4	0.5	0.0	0.2	0.0	0.1
98	0.56	0.2	0.1	0.0	0.1	2.6	0.0	0.0	0.1	0.0	0.4	1.1	0.0	0.5	0.0	0.1
99	1.05	0.2	0.1	0.0	0.1	2.3	0.0	0.0	0.2	0.0	0.3	1.0	0.0	0.5	0.0	0.1
100	0.26	3.6	1.2	2.1	0.9	1.8	0.1	10.8	15.8	2.5	4.6	1.3	11.4	1.1	6.2	0.5
Base	90.44	4.96	4.97	4.97	4.96	4.95	4.98	4.97	4.96	5.00	5.02	4.97	4.96	4.97	4.97	4.96

Supplementary Figure 26: Enrichment of all conservation states for top 5% of bases prioritized by variant prioritization scores. The figure displays for top 5% prioritized bases **(a)** genome-wide and **(b)** in non-coding regions by variant prioritization scores the same enrichments as shown in **Supplementary Fig. 24a,b** except here enrichments for all conservation states are shown and also included is the original version of the CADD score in addition to v1.4. Coloring of enrichments is based on their value in a column specific manner. The second columns gives the percentage of the background region used to compute the enrichments falling in each state, which is for **(a)** the whole genome and for **(b)** bases scored by both LINSIGHT and FunSeq2. The last line in both heatmaps gives the actual percentage of the background set covered by each set of prioritized bases, which can differ from 5.00% because of how ties were handled.

a Enrichments of top 10% ranked bases genome-wide

State	Genome %	CADD 1.4	CADD 1.0	Eigen	Eigen-PC	DANN	GERP++	phyloP	PhastCons	REMM	fitCons	FIRE	FATHMM-XF	CDTS
1	0.60	9.7	8.9	8.3	8.0	8.5	8.8	8.9	9.4	9.0	7.8	2.7	8.9	4.3
2	0.84	7.8	6.9	8.5	4.9	2.7	7.3	7.2	7.6	7.0	2.9	0.9	9.1	2.1
3	0.46	6.8	5.4	7.0	3.5	2.8	5.0	3.4	5.2	5.8	4.6	1.9	7.4	2.8
4	0.60	6.9	6.0	7.4	4.0	2.8	6.2	5.1	5.8	6.1	3.4	1.6	7.1	2.3
5	1.66	6.1	5.3	7.8	3.4	1.4	6.4	5.0	5.2	5.6	1.9	0.9	5.9	1.8
6	3.59	2.5	2.5	3.9	1.9	1.0	4.0	3.0	2.0	2.9	1.3	0.9	2.4	1.4
7	0.85	2.6	2.8	4.3	2.5	1.3	3.2	2.1	1.4	2.8	2.8	2.3	4.0	2.4
8	0.73	2.9	3.0	4.8	2.1	0.8	2.9	1.4	1.7	2.8	1.9	1.2	3.4	1.6
9	0.90	1.4	1.7	2.2	1.6	0.7	1.8	0.7	0.7	1.9	1.3	0.9	1.5	1.4
10	0.86	1.0	1.4	1.6	1.6	0.6	1.0	0.4	0.5	1.6	1.3	1.0	1.4	1.4
11	0.75	0.9	1.2	1.4	1.4	0.8	1.9	1.4	0.6	1.6	1.2	1.0	1.2	1.3
12	0.75	1.0	1.3	1.4	1.2	0.9	2.1	1.6	0.7	1.5	1.0	0.7	1.3	1.1
13	1.24	1.2	1.5	1.9	1.3	0.9	2.6	1.8	0.8	1.7	1.0	0.8	1.4	1.2
14	0.94	1.3	1.5	2.1	1.8	0.9	2.4	1.6	0.8	2.1	1.5	1.3	1.3	1.5
15	0.77	1.0	1.4	1.7	1.7	0.6	1.1	0.4	0.4	1.7	1.4	1.0	1.4	1.4
16	0.72	0.5	0.8	0.6	1.1	0.6	0.8	0.3	0.2	1.0	1.0	0.8	0.8	1.1
17	2.14	1.0	1.2	1.4	1.1	0.9	2.6	2.1	0.7	1.5	1.0	0.8	1.4	1.1
18	0.94	0.4	0.8	0.6	1.2	0.6	0.5	0.2	0.2	1.0	1.1	0.8	0.8	1.2
19	1.07	1.6	1.9	2.5	1.8	0.7	1.3	0.6	0.8	2.0	1.5	1.0	1.9	1.5
20	1.03	1.2	1.5	1.9	1.5	0.9	2.3	1.5	0.8	1.8	1.2	0.9	1.4	1.3
21	0.64	1.0	1.2	1.5	1.4	0.8	1.9	1.4	0.6	1.6	1.2	0.9	1.3	1.2
22	0.41	0.7	0.9	0.9	1.4	0.9	1.5	1.2	0.4	1.4	1.2	1.1	0.9	1.3
23	0.63	0.6	0.7	0.4	1.0	0.9	0.7	1.4	0.5	1.0	1.1	1.2	0.8	1.1
24	0.53	0.5	0.6	0.4	0.9	0.9	0.8	1.3	0.4	0.9	0.9	0.9	0.8	1.0
25	0.61	0.5	0.7	0.4	0.8	0.8	1.1	1.2	0.4	0.9	0.8	0.8	0.9	1.0
26	0.72	0.7	0.9	0.7	0.9	0.8	1.5	1.3	0.5	1.1	1.0	1.0	1.1	1.1
27	0.96	0.4	0.7	0.5	1.1	0.6	0.4	0.2	0.2	0.9	1.2	1.1	0.7	1.2
28	0.44	2.6	2.0	3.3	4.3	2.7	1.6	2.0	2.0	3.2	3.1	2.1	2.3	1.8
29	0.92	0.5	0.7	0.4	0.9	0.8	1.1	1.1	0.3	0.9	0.7	0.6	0.9	0.9
30	0.94	0.5	0.6	0.3	0.7	0.8	0.9	1.2	0.4	0.8	0.7	0.7	0.8	0.9
31	0.63	0.5	0.6	0.3	0.9	0.9	0.6	1.3	0.4	0.9	0.9	1.0	0.7	1.0
32	0.93	0.6	0.6	0.2	0.7	1.1	0.1	2.0	0.6	0.6	0.8	1.0	0.5	0.9
33	0.45	0.8	1.0	0.3	0.6	1.5	0.3	1.6	0.6	0.6	0.7	1.0	0.5	0.8
34	0.51	1.0	1.3	0.9	0.9	1.1	1.5	1.4	0.6	1.1	0.9	1.1	1.1	1.0
35	1.20	0.8	1.0	1.1	1.4	0.9	1.7	1.6	0.7	1.5	1.4	1.4	1.2	1.3
36	1.34	0.6	0.8	0.5	0.8	0.9	1.3	1.5	0.6	1.0	0.9	1.0	1.1	1.0
37	2.30	1.1	1.3	1.5	1.2	0.9	2.3	1.8	0.8	1.6	1.2	1.1	1.3	1.2
38	0.91	0.8	1.0	0.8	0.9	0.8	1.8	1.6	0.6	1.2	1.0	1.0	1.1	1.1
39	0.70	0.6	0.8	0.5	0.8	0.8	1.3	1.3	0.5	1.0	0.8	0.8	1.0	1.0
40	0.76	0.5	0.6	0.3	0.7	0.9	0.7	1.3	0.4	0.8	0.7	0.8	0.8	0.9
41	0.70	0.6	0.8	0.6	0.9	0.8	1.4	1.3	0.4	1.0	0.7	0.6	1.0	1.0
42	0.74	0.6	0.8	0.6	1.2	0.9	1.3	1.3	0.4	1.2	1.0	1.0	0.9	1.1
43	1.00	0.6	0.7	0.4	1.0	0.9	0.8	1.4	0.5	1.0	1.0	1.0	0.8	1.1
44	1.02	0.7	0.7	0.3	0.8	1.2	0.2	2.0	0.6	0.7	0.9	1.2	0.7	1.0
45	0.84	0.5	0.7	0.3	0.8	0.9	0.7	1.4	0.4	0.9	0.8	0.9	0.8	1.0
46	0.70	0.6	0.8	0.5	0.9	0.9	1.1	1.3	0.5	1.0	1.0	1.2	0.9	1.1
47	0.41	0.2	0.4	0.3	1.4	0.4	0.1	0.0	0.0	0.5	1.3	1.0	0.7	1.2
48	0.53	0.2	0.4	0.2	0.9	0.4	0.1	0.1	0.2	0.3	0.9	0.9	0.6	1.0
49	0.61	0.3	0.5	0.5	1.3	0.4	0.3	0.2	0.2	0.5	1.3	1.2	0.7	1.3
50	0.79	0.9	0.9	1.6	1.8	0.3	0.9	0.5	0.8	0.5	1.5	1.1	1.8	1.5
51	0.74	0.2	0.4	0.4	1.3	0.3	0.3	0.2	0.2	0.3	1.1	0.9	0.7	1.2
52	0.50	0.3	0.6	0.5	1.4	0.5	0.3	0.1	0.1	0.9	1.1	0.9	0.8	1.2
53	0.50	1.1	1.4	1.9	1.9	0.6	0.7	0.3	0.5	1.6	1.7	1.2	1.8	1.6
54	0.27	3.0	2.5	3.6	2.5	1.4	1.4	1.3	2.4	1.2	4.0	2.1	5.2	2.5
55	0.30	0.9	1.3	1.5	1.7	0.5	1.1	0.6	0.9	0.1	1.5	1.1	1.7	1.5
56	0.38	0.2	0.7	0.3	1.2	0.5	0.4	0.2	0.2	0.0	1.0	0.8	0.6	1.1
57	0.39	0.1	0.6	0.2	1.0	0.5	0.2	0.1	0.2	0.0	1.0	1.0	0.5	1.1
58	0.46	0.2	0.4	0.2	1.1	0.4	0.1	0.1	0.1	0.4	1.0	1.0	0.4	1.1
59	0.53	0.2	0.3	0.2	1.2	0.6	0.0	0.0	0.2	0.4	1.1	1.1	0.3	1.0
60	0.30	0.1	0.3	0.1	0.9	0.4	0.0	0.0	0.1	0.3	0.8	0.7	0.4	1.0
61	0.48	0.1	0.3	0.1	0.9	0.4	0.1	0.1	0.1	0.3	0.8	0.7	0.3	0.9
62	0.44	0.1	0.3	0.2	1.0	0.4	0.1	0.1	0.1	0.3	1.0	1.0	0.4	1.0
63	0.41	0.3	0.5	0.6	1.5	0.4	0.3	0.2	0.2	0.6	1.3	1.0	0.7	1.3
64	0.79	0.5	0.7	0.4	0.9	0.9	0.9	1.2	0.4	0.9	0.8	0.9	0.8	1.0
65	0.93	0.4	0.5	0.2	1.0	0.8	0.2	1.3	0.3	0.7	0.9	1.0	0.4	1.0
66	0.67	0.6	0.7	0.7	1.4	0.8	0.9	0.9	0.3	1.2	1.1	1.1	0.8	1.2
67	0.98	0.4	0.5	0.4	1.1	0.8	0.3	1.2	0.2	0.8	1.0	1.0	0.5	1.1
68	1.17	0.4	0.4	0.1	0.6	0.9	0.1	1.2	0.3	0.5	0.8	1.0	0.3	0.9
69	0.92	0.5	0.6	0.2	0.7	1.1	0.1	1.8	0.5	0.6	0.7	0.9	0.4	0.9
70	0.51	0.2	0.3	0.1	0.9	0.6	0.0	0.0	0.2	0.3	0.8	0.9	0.1	0.9
71	0.41	0.2	0.3	0.1	1.0	0.6	0.0	0.0	0.1	0.3	0.9	1.1	0.2	1.0
72	1.21	0.6	0.6	0.2	0.8	1.1	0.1	1.8	0.5	0.6	0.8	1.1	0.3	0.9
73	0.74	0.4	0.5	0.1	0.7	1.0	0.0	0.9	0.4	0.4	0.9	1.4	0.2	1.0
74	1.49	0.4	0.3	0.2	0.7	0.8	0.1	0.2	0.4	0.5	1.0	1.4	0.2	1.1
75	2.47	0.5	0.5	0.1	0.5	1.0	0.0	0.3	0.4	0.3	1.0	1.5	0.2	1.1
76	3.92	0.8	0.6	0.1	0.4	1.2	0.0	0.0	1.1	0.2	0.7	1.0	0.1	0.9
77	0.98	0.5	0.4	0.1	0.4	1.1	0.0	0.1	0.7	0.2	1.1	2.0	0.1	1.0
78	1.14	0.7	0.5	0.1	0.4	1.0	0.0	0.0	0.9	0.3	0.8	1.4	0.1	0.9
79	3.18	0.9	0.7	0.1	0.4	1.6	0.0	0.0	1.5	0.1	0.7	1.1	0.1	0.8
80	1.24	0.4	0.4	0.1	0.4	1.1	0.0	0.2	0.4	0.2	0.9	1.8	0.1	1.0
81	0.98	0.7	0.5	0.1	0.5	1.4	0.0	0.2	1.0	0.1	0.8	1.3	0.1	0.9
82	0.54	1.1	0.6	0.7	2.1	1.1	0.1	1.9	2.4	0.8	1.7	1.5	1.3	0.8
83	1.29	1.0	0.7	0.1	0.3	1.4	0.0	0.0	1.1	0.1	0.5	0.9	0.0	0.6
84	0.86	0.6	0.5	0.1	0.4	1.3	0.0	0.3	0.6	0.2	0.7	1.3	0.1	0.6
85	1.38	0.7	0.5	0.1	0.2	1.4	0.0	0.0	1.3	0.0	0.7	1.6	0.0	0.7
86	1.56	0.9	0.6	0.1	0.2	1.8	0.0	0.0	1.6	0.0	0.5	1.0	0.0	0.6
87	0.63	0.0	0.1	0.0	0.3	0.3	0.0	0.0	0.2	0.0	0.8	1.3	0.1	0.9
88	0.66	0.0	0.1	0.0	0.4	0.3	0.0	0.0	0.2	0.0	0.9	1.2	0.1	0.9
89	0.38	0.1	0.4	0.1	1.0	0.5	0.1	0.0	0.1	0.0	0.9	0.9	0.2	0.9
90	1.09	1.4	1.1	0.2	0.3	1.8	0.0	0.0	1.0	0.2	0.6	1.1	0.1	0.8
91	0.99	0.6	0.6	0.1	0.5	1.5	0.0	0.5	0.5	0.2	0.7	1.2	0.1	0.8
92	1.81	0.8	0.5	0.0	0.2	2.1	0.0	0.0	1.7	0.0	0.5	0.9	0.0	0.6
93	0.67	1.0	0.7	0.0	0.3	2.4	0.0	0.0	1.9	0.0	0.4	0.9	0.1	0.6
94	0.65	0.2	0.1	0.0	0.2	0.9	0.0	0.0	2.3	0.0	0.4	0.7	0.0	0.3
95	1.06	0.3	0.2	0.1	0.5	0.8	0.0	0.4	2.1	0.1	0.6	0.8	0.3	0.4
96	8.86	0.0	0.0	0.0	0.0	0.1	0.0	0.0	0.0	0.0	0.0	0.1	0.2	0.0
97	1.13	0.1	0.0	0.0	0.1	0.2	0.0	0.0	2.5	0.0	0.3	0.5	0.0	0.2
98	0.51	0.7	0.4	0.0	0.2	1.8	0.0	0.0	1.9	0.0	0.5	1.1	0.0	0.5
99	0.97	0.6	0.4	0.0	0.2	1.5	0.0	0.0	1.5	0.0	0.5	1.1	0.0	0.5
100	0.25	4.2	1.6	2.5	1.1	1.7	0.3	6.3	8.2	2.2	2.7	1.6	6.5	0.9
Base	100	10.00	10.00	9.95	9.95	10.00	10.02	10.00	10.00	10.03	10.88	10.03	10.00	10.00

b Enrichments of top 10% ranked bases non-coding restricted

State	Non coding region %	CADD 1.4	CADD 1.0	Eigen	Eigen-PC	DANN	GERP++	phyloP	PhastCons	REMM	fitCons	FIRE	FATHMM-XF	CDTS	LINSIGHT	FunSeq2
1	0.13	9.1	7.7	9.3	6.9	5.2	8.3	9.0	9.0	8.5	3.6	1.2	9.6	3.0	9.5	4.9
2	0.83	7.7	6.8	8.7	4.5	2.0	7.2	7.1	7.6	6.9	2.2	0.6	9.3	1.8	8.4	3.6
3	0.33	6.0	5.0	7.5	2.9	0.9	5.0	3.4	4.8	5.2	2.5	1.1	8.7	2.0	7.4	3.1
4	0.54	6.4	5.5	7.7	3.4	1.5	5.9	4.8	5.5	5.7	2.3	1.1	7.3	2.0	7.5	3.4
5	1.80	6.0	5.3	7.8	3.1	1.2	6.3	4.9	5.2	5.5	1.8	0.9	5.9	1.6	7.1	3.2
6	3.95	2.5	2.5	3.8	1.8	1.0	3.9	2.9	2.0	2.9	1.3	0.8	2.4	1.3	3.5	2.3
7	0.91	2.5	2.7	4.3	2.4	1.2	3.1	2.0	1.4	2.7	2.7	2.2	4.0	2.2	3.9	2.9
8	0.79	2.9	3.0	4.7	2.0	0.7	2.8	1.4	1.7	2.8	1.8	1.1	3.3	1.5	4.3	2.2
9	0.99	1.4	1.7	2.1	1.6	0.7	1.8	0.7	0.7	1.9	1.3	0.9	1.5	1.3	2.2	1.6
10	0.95	1.0	1.4	1.6	1.6	0.6	1.0	0.4	0.5	1.6	1.3	0.9	1.3	1.3	2.0	1.4
11	0.83	0.9	1.2	1.4	1.4	0.8	1.9	1.3	0.6	1.6	1.2	1.0	1.2	1.2	1.4	1.5
12	0.83	1.0	1.2	1.4	1.2	0.9	2.1	1.5	0.7	1.5	1.0	0.7	1.3	1.0	1.5	1.3
13	1.37	1.2	1.5	1.8	1.2	0.8	2.5	1.7	0.8	1.7	1.0	0.7	1.3	1.1	1.8	1.6
14	1.03	1.3	1.5	2.0	1.8	0.9	2.3	1.6	0.8	2.1	1.5	1.3	1.2	1.4	1.8	2.0
15	0.85	1.0	1.4	1.6	1.7	0.6	1.1	0.4	0.4	1.7	1.4	1.0	1.3	1.4	2.0	1.5
16	0.79	0.5	0.8	0.6	1.1	0.6	0.8	0.3	0.2	1.0	1.0	0.8	0.8	1.1	0.7	1.0
17	2.36	1.0	1.2	1.3	1.1	0.9	2.5	2.1	0.7	1.5	1.0	0.8	1.3	1.1	1.3	1.5
18	1.04	0.4	0.7	0.6	1.2	0.6	0.5	0.2	0.2	1.0	1.0	0.8	0.7	1.1	0.9	1.0
19	1.17	1.5	1.8	2.4	1.8	0.6	1.3	0.6	0.8	2.0	1.5	1.0	1.8	1.4	2.8	1.6
20	1.13	1.2	1.4	1.8	1.4	0.9	2.2	1.5	0.8	1.7	1.2	0.8	1.3	1.2	1.8	1.6
21	0.71	1.0	1.2	1.4	1.3	0.8	1.9	1.3	0.6	1.6	1.2	0.9	1.3	1.2	1.6	1.4
22	0.45	0.7	0.9	0.9	1.3	0.9	1.4	1.2	0.4	1.4	1.2	1.0	0.9	1.2	0.9	1.3
23	0.69	0.6	0.6	0.4	1.0	0.9	0.7	1.4	0.5	1.0	1.1	1.1	0.8	1.0	0.3	1.0
24	0.59	0.5	0.6	0.3	0.8	0.9	0.7	1.3	0.4	0.9	0.9	0.9	0.8	0.9	0.3	0.8
25	0.68	0.5	0.7	0.4	0.8	0.8	1.1	1.1	0.4	0.9	0.8	0.7	0.9	0.9	0.4	0.9
26	0.80	0.7	0.9	0.7	0.9	0.8	1.5	1.3	0.5	1.1	1.0	0.9	1.0	1.0	0.5	1.2
27	1.05	0.4	0.7	0.5	1.1	0.6	0.4	0.2	0.2	0.9	1.2	1.0	0.7	1.1	0.6	1.0
28	0.44	2.2	1.6	3.4	4.4	2.3	1.5	1.8	1.7	3.0	2.7	1.8	2.2	1.8	2.8	2.5
29	1.01	0.5	0.7	0.4	0.9	0.8	1.1	1.1	0.3	0.9	0.7	0.6	0.8	0.9	0.6	0.8
30	1.03	0.5	0.6	0.3	0.7	0.8	0.9	1.2	0.4	0.8	0.7	0.7	0.8	0.9	0.2	0.7
31	0.70	0.5	0.6	0.3	0.8	0.9	0.6	1.3	0.4	0.9	0.9	0.9	0.7	0.9	0.2	0.7
32	1.00	0.6	0.6	0.2	0.7	1.1	0.1	2.0	0.6	0.6	0.8	0.9	0.5	0.9	0.1	0.6
33	0.49	0.8	1.0	0.3	0.6	1.5	0.3	1.6	0.6	0.6	0.7	0.9	0.5	0.8	0.3	0.5
34	0.56	1.0	1.2	0.9	0.8	1.1	1.4	1.4	0.6	1.1	0.9	1.0	1.1	1.0	0.9	1.0
35	1.33	0.8	1.0	1.1	1.4	0.9	1.6	1.5	0.7	1.5	1.4	1.3	1.1	1.3	0.8	1.6
36	1.47	0.6	0.8	0.5	0.8	0.9	1.3	1.5	0.6	1.0	0.9	0.9	1.1	1.0	0.4	1.0
37	2.54	1.1	1.2	1.5	1.2	0.8	2.2	1.7	0.8	1.5	1.2	1.0	1.3	1.1	1.2	1.6
38	1.00	0.8	0.9	0.8	0.9	0.8	1.8	1.5	0.6	1.1	1.0	0.9	1.1	1.0	0.6	1.2
39	0.76	0.6	0.8	0.5	0.8	0.8	1.2	1.3	0.5	0.9	0.8	0.8	1.0	0.9	0.5	0.9
40	0.83	0.5	0.6	0.3	0.7	0.9	0.7	1.2	0.4	0.7	0.7	0.7	0.8	0.9	0.2	0.7
41	0.78	0.6	0.8	0.6	0.9	0.8	1.4	1.3	0.4	1.0	0.7	0.6	1.0	0.9	0.6	0.9
42	0.82	0.6	0.8	0.6	1.2	0.9	1.3	1.2	0.4	1.2	1.0	1.0	0.9	1.1	0.6	1.1
43	1.10	0.6	0.7	0.4	0.9	0.9	0.8	1.3	0.5	1.0	1.0	1.0	0.8	1.0	0.3	0.9
44	1.12	0.7	0.7	0.3	0.8	1.2	0.1	1.9	0.6	0.7	0.9	1.1	0.7	0.9	0.2	0.6
45	0.93	0.5	0.7	0.3	0.8	0.9	0.7	1.4	0.4	0.8	0.8	0.9	0.7	0.9	0.3	0.7
46	0.77	0.6	0.7	0.5	0.9	0.9	1.0	1.3	0.5	1.0	1.0	1.1	0.9	1.0	0.4	1.0
47	0.45	0.2	0.4	0.3	1.4	0.4	0.1	0.0	0.0	0.5	1.2	0.9	0.7	1.2	0.7	1.0
48	0.59	0.2	0.3	0.2	0.9	0.4	0.1	0.1	0.2	0.3	0.9	0.9	0.5	1.0	0.3	0.7
49	0.67	0.3	0.4	0.4	1.3	0.4	0.2	0.2	0.2	0.5	1.3	1.1	0.7	1.2	0.6	1.1
50	0.87	0.9	0.9	1.6	1.8	0.3	0.9	0.5	0.8	0.5	1.5	1.0	1.7	1.5	2.4	1.5
51	0.81	0.2	0.4	0.4	1.3	0.3	0.3	0.2	0.2	0.3	1.1	0.8	0.6	1.1	0.8	0.9
52	0.55	0.3	0.6	0.5	1.4	0.5	0.3	0.1	0.1	0.9	1.1	0.9	0.7	1.1	0.9	1.0
53	0.55	1.1	1.4	1.8	1.9	0.6	0.6	0.3	0.5	1.6	1.6	1.1	1.7	1.5	2.5	1.5
54	0.23	2.2	1.9	3.4	2.3	0.5	1.4	1.2	2.0	1.0	2.6	1.6	5.8	2.1	4.4	2.0
55	0.33	0.9	1.3	1.5	1.7	0.4	1.1	0.5	0.9	0.1	1.5	1.0	1.7	1.4	2.4	1.5
56	0.42	0.2	0.7	0.3	1.1	0.5	0.4	0.2	0.2	0.0	1.0	0.8	0.6	1.0	0.7	0.9
57	0.43	0.1	0.6	0.2	1.0	0.5	0.2	0.1	0.2	0.0	1.0	0.9	0.5	1.0	0.3	0.8
58	0.51	0.2	0.4	0.2	1.1	0.4	0.1	0.1	0.1	0.4	1.0	1.0	0.4	1.0	0.4	0.8
59	0.58	0.2	0.3	0.2	1.2	0.5	0.0	0.0	0.2	0.4	1.1	1.0	0.3	1.0	0.2	0.7
60	0.34	0.1	0.3	0.1	0.9	0.4	0.0	0.0	0.1	0.3	0.8	0.7	0.3	0.9	0.2	0.6
61	0.53	0.1	0.3	0.1	0.9	0.4	0.1	0.0	0.1	0.3	0.8	0.7	0.3	0.9	0.2	0.6
62	0.49	0.1	0.3	0.2	1.0	0.4	0.1	0.1	0.1	0.3	1.0	0.9	0.4	1.0	0.2	0.8
63	0.45	0.3	0.5	0.6	1.5	0.4	0.3	0.2	0.2	0.6	1.3	0.9	0.7	1.2	0.9	1.1
64	0.87	0.5	0.7	0.4	0.8	0.9	0.9	1.1	0.4	0.9	0.8	0.8	0.7	0.9	0.4	0.8
65	1.03	0.4	0.5	0.2	1.0	0.8	0.2	1.3	0.3	0.7	0.9	1.0	0.4	1.0	0.2	0.7
66	0.73	0.6	0.7	0.6	1.3	0.8	0.9	0.9	0.3	1.2	1.1	1.0	0.7	1.1	0.9	1.1
67	1.08	0.4	0.5	0.4	1.1	0.8	0.3	1.2	0.2	0.8	1.0	0.9	0.5	1.0	0.5	0.8
68	1.30	0.4	0.4	0.1	0.6	0.9	0.1	1.2	0.3	0.5	0.8	1.0	0.2	0.9	0.1	0.5
69	1.01	0.5	0.6	0.2	0.7	1.1	0.1	1.7	0.5	0.5	0.7	0.8	0.4	0.8	0.1	0.5
70	0.55	0.2	0.3	0.1	0.9	0.6	0.0	0.0	0.2	0.3	0.8	0.8	0.1	0.9	0.1	0.6
71	0.45	0.2	0.3	0.1	1.0	0.6	0.0	0.0	0.1	0.3	0.9	1.0	0.2	0.9	0.1	0.6
72	1.31	0.5	0.6	0.2	0.8	1.1	0.1	1.7	0.5	0.6	0.8	1.1	0.3	0.9	0.1	0.6
73	0.81	0.4	0.4	0.1	0.6	1.0	0.0	0.9	0.4	0.4	0.9	1.3	0.2	1.0	0.1	0.6
74	1.64	0.4	0.3	0.2	0.7	0.8	0.1	0.2	0.4	0.5	1.0	1.3	0.2	1.0	0.1	0.7
75	2.73	0.5	0.4	0.1	0.5	1.0	0.0	0.2	0.4	0.3	1.0	1.4	0.1	1.0	0.1	0.6
76	4.33	0.8	0.6	0.1	0.4	1.2	0.0	0.0	1.1	0.2	0.7	1.0	0.1	0.8	0.1	0.4
77	1.08	0.5	0.4	0.1	0.4	1.1	0.0	0.1	0.7	0.2	1.1	1.8	0.1	1.0	0.1	0.6
78	1.26	0.7	0.5	0.1	0.4	1.0	0.0	0.0	0.9	0.2	0.8	1.3	0.1	0.9	0.1	0.5
79	3.51	0.9	0.6	0.1	0.3	1.6	0.0	0.0	1.5	0.1	0.7	1.0	0.1	0.8	0.0	0.4
80	1.37	0.4	0.4	0.1	0.4	1.1	0.0	0.2	0.4	0.2	0.9	1.6	0.1	1.0	0.1	0.6
81	1.08	0.7	0.5	0.1	0.5	1.4	0.0	0.2	1.0	0.1	0.8	1.2	0.1	0.9	0.1	0.4
82	0.57	1.0	0.5	0.7	2.1	1.0	0.1	1.8	2.3	0.8	1.6	1.4	1.3	0.8	1.3	0.8
83	1.38	1.0	0.7	0.1	0.2	1.4	0.0	0.0	1.1	0.1	0.6	0.9	0.0	0.6	0.0	0.3
84	0.91	0.6	0.5	0.1	0.4	1.3	0.0	0.2	0.6	0.2	0.7	1.2	0.1	0.6	0.1	0.4
85	1.46	0.7	0.5	0.0	0.2	1.4	0.0	0.0	1.3	0.0	0.7	1.5	0.0	0.7	0.0	0.4
86	1.73	0.9	0.6	0.0	0.2	1.8	0.0	0.0	1.6	0.0	0.5	0.9	0.0	0.6	0.0	0.3
87	0.69	0.0	0.1	0.0	0.3	0.3	0.0	0.0	0.2	0.0	0.8	1.2	0.1	0.8	0.0	0.5
88	0.73	0.0	0.1	0.0	0.4	0.3	0.0	0.0	0.2	0.0	0.9	1.1	0.1	0.9	0.1	0.5
89	0.41	0.1	0.4	0.1	1.0	0.5	0.0	0.0	0.1	0.0	0.9	0.9	0.2	0.9	0.2	0.6
90	1.19	1.4	1.1	0.2	0.3	1.8	0.0	0.0	1.0	0.2	0.6	1.0	0.1	0.8	0.1	0.4
91	1.07	0.6	0.6	0.1	0.5	1.5	0.0	0.5	0.5	0.2	0.7	1.1	0.1	0.8	0.1	0.4
92	2.00	0.8	0.5	0.0	0.2	2.1	0.0	0.0	1.6	0.0	0.5	0.9	0.0	0.6	0.0	0.3
93	0.74	1.0	0.6	0.0	0.2	2.4	0.0	0.0	1.9	0.0	0.4	0.8	0.0	0.5	0.0	0.2
94	0.72	0.2	0.1	0.0	0.2	0.9	0.0	0.0	2.3	0.0	0.4	0.6	0.0	0.3	0.0	0.2
95	1.08	0.3	0.2	0.1	0.5	0.9	0.0	0.3	2.1	0.1	0.6	0.8	0.3	0.4	0.2	0.3
96	1.33	0.0	0.0	0.0	0.2	1.0	0.0	0.0	0.1	0.0	0.3	0.4	1.5	0.1	0.0	0.2
97	0.96	0.1	0.0	0.0	0.1	0.2	0.0	0.0	2.5	0.0	0.4	0.6	0.0	0.2	0.0	0.2
98	0.56	0.7	0.4	0.0	0.2	1.8	0.0	0.0	1.9	0.0	0.5	1.1	0.0	0.5	0.0	0.2
99	1.05	0.5	0.4	0.0	0.2	1.5	0.0	0.0	1.5	0.0	0.5	1.0	0.0	0.4	0.0	0.3
100	0.26	4.0	1.5	2.5	1.1	1.5	0.3	6.3	8.3	2.2	2.5	1.6	6.8	0.9	5.4	0.6
Base	90.44	9.92	9.93	9.93	9.91	9.91	10.01	9.93	9.93	9.96	10.74	9.91	9.93	9.93	9.93	9.91

Supplementary Figure 27: Enrichment of all conservation states for top 10% of bases prioritized by variant prioritization scores. The figure displays for top 10% prioritized bases **(a)** genome-wide and **(b)** in non-coding regions by variant prioritization scores the same enrichments as shown in **Supplementary Fig. 24c,d** except here enrichments for all conservation states are shown and also included is the original version of the CADD score in addition to v1.4. Coloring of enrichments is based on their value in a column specific manner. The second columns gives the percentage of the background region used to compute the enrichments falling in each state, which is for **(a)** the whole genome and for **(b)** bases scored by both LINSIGHT and FunSeq2. The last line in both heatmaps gives the actual percentage of the background set covered by each set of prioritized bases, which can differ from 10.00% because of how ties were handled.

	MAF < 0.001	0.001 <= MAF < 0.01	0.01 <= MAF < 0.1	0.1 <= MAF < 0.2	0.2 <= MAF < 0.3	0.3 <= MAF < 0.4	0.4 <= MAF < 0.5
1	-0.52	-1.05	-1.64	-2.26	-2.53	-2.66	-2.80
2	-0.20	-0.31	-0.63	-1.04	-1.26	-1.43	-1.52
3	0.22	0.08	-0.21	-0.60	-0.75	-0.92	-1.03
4	-0.10	-0.21	-0.50	-0.88	-1.07	-1.26	-1.30
5	-0.19	-0.24	-0.48	-0.81	-1.04	-1.17	-1.27
6	-0.17	-0.20	-0.35	-0.64	-0.80	-0.95	-1.00
7	-0.06	-0.09	-0.31	-0.64	-0.82	-0.98	-1.00
8	0.06	0.05	-0.14	-0.40	-0.61	-0.68	-0.76
9	0.01	0.00	-0.16	-0.42	-0.55	-0.69	-0.80
10	0.10	0.11	-0.04	-0.29	-0.45	-0.58	-0.65
11	-0.06	-0.04	-0.17	-0.36	-0.51	-0.68	-0.74
12	-0.11	-0.06	-0.20	-0.41	-0.55	-0.72	-0.74
13	-0.10	-0.06	-0.20	-0.42	-0.59	-0.75	-0.81
14	-0.11	-0.08	-0.22	-0.49	-0.64	-0.77	-0.82
15	0.16	0.15	0.01	-0.19	-0.32	-0.39	-0.45
16	0.12	0.14	0.04	-0.16	-0.26	-0.37	-0.38
17	-0.16	-0.15	-0.27	-0.52	-0.64	-0.78	-0.85
18	0.14	0.17	0.05	-0.21	-0.34	-0.45	-0.50
19	0.13	0.11	-0.04	-0.33	-0.48	-0.59	-0.69
20	-0.04	-0.04	-0.17	-0.44	-0.63	-0.70	-0.78
21	-0.06	-0.02	-0.13	-0.37	-0.54	-0.68	-0.70
22	-0.03	-0.01	-0.13	-0.39	-0.50	-0.63	-0.70
23	-0.03	0.00	-0.11	-0.32	-0.43	-0.58	-0.59
24	-0.08	-0.01	-0.12	-0.32	-0.43	-0.55	-0.59
25	-0.03	0.00	-0.11	-0.31	-0.43	-0.57	-0.63
26	-0.09	-0.05	-0.17	-0.40	-0.55	-0.64	-0.71
27	0.12	0.15	0.04	-0.21	-0.36	-0.50	-0.55
28	-0.04	-0.13	-0.28	-0.56	-0.71	-0.74	-0.85
29	-0.04	0.01	-0.08	-0.29	-0.46	-0.58	-0.56
30	-0.07	-0.02	-0.11	-0.34	-0.47	-0.57	-0.65
31	-0.04	0.01	-0.09	-0.29	-0.44	-0.57	-0.60
32	-0.07	-0.08	-0.17	-0.41	-0.54	-0.64	-0.72
33	-0.13	-0.09	-0.16	-0.31	-0.39	-0.53	-0.62
34	-0.09	-0.06	-0.16	-0.36	-0.50	-0.55	-0.65
35	-0.08	-0.05	-0.17	-0.39	-0.54	-0.70	-0.71
36	-0.10	-0.07	-0.18	-0.38	-0.53	-0.63	-0.67
37	-0.14	-0.10	-0.25	-0.50	-0.64	-0.76	-0.83
38	-0.09	-0.05	-0.17	-0.40	-0.60	-0.68	-0.71
39	-0.14	-0.09	-0.19	-0.41	-0.55	-0.64	-0.69
40	-0.05	-0.01	-0.11	-0.33	-0.42	-0.55	-0.61
41	-0.06	0.00	-0.11	-0.33	-0.45	-0.53	-0.65
42	-0.05	-0.02	-0.13	-0.36	-0.46	-0.62	-0.67
43	-0.03	0.01	-0.07	-0.30	-0.42	-0.52	-0.61
44	-0.20	-0.18	-0.27	-0.43	-0.60	-0.66	-0.74
45	-0.06	-0.03	-0.11	-0.32	-0.47	-0.59	-0.66
46	-0.10	-0.09	-0.22	-0.42	-0.55	-0.65	-0.70
47	0.42	0.41	0.33	0.18	0.02	-0.05	-0.09
48	0.31	0.31	0.20	0.00	-0.10	-0.27	-0.37
49	0.34	0.33	0.20	-0.04	-0.19	-0.30	-0.32
50	0.50	0.43	0.27	0.04	-0.12	-0.26	-0.27
51	0.46	0.43	0.31	0.12	-0.03	-0.12	-0.17
52	0.14	0.16	0.02	-0.22	-0.38	-0.50	-0.61
53	0.21	0.17	0.00	-0.30	-0.48	-0.60	-0.68
54	0.87	0.83	1.08	1.44	1.65	1.79	1.81
55	1.50	1.69	2.70	3.59	3.92	4.12	4.21
56	1.46	1.65	2.62	3.50	3.83	4.02	4.12
57	1.47	1.69	2.72	3.61	3.95	4.14	4.24
58	0.38	0.37	0.27	0.11	0.02	-0.10	-0.15
59	0.34	0.35	0.30	0.14	0.11	0.04	-0.02
60	0.36	0.37	0.26	0.07	-0.03	-0.11	-0.19
61	0.38	0.39	0.28	0.11	-0.01	-0.10	-0.18
62	0.39	0.38	0.29	0.11	0.01	-0.08	-0.13
63	0.39	0.37	0.26	0.03	-0.12	-0.23	-0.28
64	-0.04	-0.02	-0.13	-0.36	-0.54	-0.62	-0.75
65	0.06	0.09	-0.01	-0.25	-0.40	-0.53	-0.60
66	0.08	0.10	-0.04	-0.26	-0.37	-0.52	-0.54
67	0.13	0.14	0.03	-0.19	-0.34	-0.46	-0.56
68	0.08	0.10	-0.02	-0.24	-0.39	-0.54	-0.60
69	-0.06	-0.01	-0.11	-0.32	-0.49	-0.57	-0.66
70	0.32	0.32	0.25	0.10	0.03	-0.10	-0.11
71	0.35	0.32	0.21	0.00	-0.14	-0.23	-0.24
72	-0.05	-0.07	-0.18	-0.41	-0.56	-0.71	-0.72
73	0.08	0.10	0.00	-0.16	-0.31	-0.43	-0.46
74	0.11	0.12	-0.01	-0.27	-0.43	-0.54	-0.60
75	0.18	0.17	0.04	-0.21	-0.36	-0.50	-0.57
76	0.18	0.15	0.04	-0.21	-0.35	-0.47	-0.56
77	0.09	0.08	-0.06	-0.33	-0.50	-0.62	-0.69
78	0.13	0.13	0.01	-0.23	-0.37	-0.49	-0.58
79	0.22	0.17	0.04	-0.19	-0.34	-0.46	-0.53
80	0.26	0.26	0.13	-0.12	-0.31	-0.45	-0.49
81	0.19	0.16	0.05	-0.16	-0.36	-0.48	-0.56
82	-0.23	-0.20	-0.23	-0.32	-0.41	-0.41	-0.41
83	-0.13	-0.21	-0.33	-0.58	-0.72	-0.79	-0.90
84	-0.43	-0.46	-0.57	-0.81	-0.96	-1.12	-1.11
85	-0.05	-0.10	-0.22	-0.47	-0.63	-0.73	-0.83
86	0.26	0.16	0.09	-0.02	-0.12	-0.18	-0.24
87	1.41	1.49	2.10	2.76	3.00	3.17	3.24
88	1.37	1.45	2.08	2.74	3.03	3.18	3.25
89	1.47	1.69	2.71	3.60	3.92	4.12	4.22
90	0.20	0.17	0.19	0.19	0.19	0.19	0.18
91	0.23	0.28	0.29	0.29	0.27	0.28	0.22
92	0.42	0.35	0.37	0.34	0.32	0.35	0.27
93	0.38	0.29	0.32	0.35	0.31	0.34	0.34
94	0.16	0.09	0.09	0.07	0.04	0.01	0.03
95	-0.10	-0.18	-0.15	-0.19	-0.25	-0.25	-0.31
96	-3.18	-3.34	-3.38	-3.51	-3.70	-3.60	-3.48
97	-0.70	-0.78	-0.79	-0.86	-0.93	-0.98	-1.05
98	0.16	0.07	0.07	0.03	0.03	-0.07	-0.05
99	0.09	0.03	0.02	-0.02	-0.05	-0.09	-0.13
100	-0.52	-0.57	-0.52	-0.55	-0.56	-0.63	-0.56
% genome	1.46	0.39	0.18	0.05	0.04	0.03	0.03

Supplementary Figure 28: Conservation state enrichments for single nucleotide variants from Ref. 10. Rows 1-100 corresponds to states, color coded based on their group, and the last line represents the percentage of the genome covered by each annotation in the columns. The table displays the log_2 fold enrichments of all the single nucleotide variants from whole genome sequencing data of 7794 unrelated individuals that were used to generate the context dependent tolerance score. The variants are grouped into disjoint sets according to minor allele frequency (MAF). Depletions are shown in shades of blue and enrichments in shades of red. The large depletions in state 96 are due to the state capturing assembly gaps.

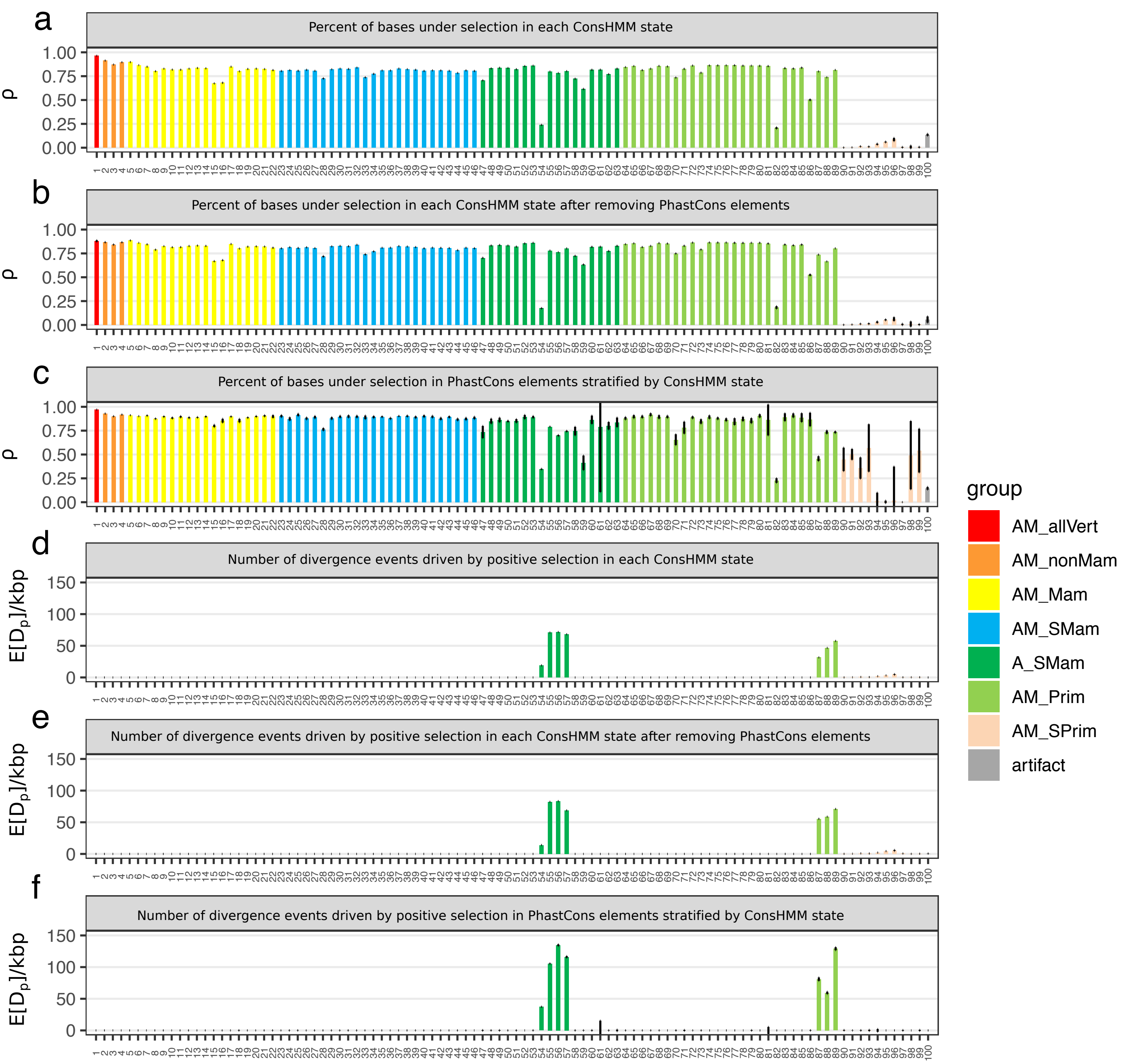

Supplementary Figure 29: Results of running the INSIGHT[11] model. (a-c) The estimated fraction of bases under selection (ρ) as estimated by the INSIGHT method within **(a)** each conservation state, **(b)** each conservation state after removing bases in PhastCons elements and **(c)** each state restricted only to bases in PhastCons elements. **(d-f)** The estimated number of divergence events driven by positive selection per kilobase-pair (E[Dp]/kbp) as estimated by the INSIGHT method within **(d)** each conservation state, **(e)** each state after removing bases in PhastCons elements and **(f)** each state restricted only to bases in PhastCons elements. States are colored according to their group as indicated on the right. Error bars represent one standard error around each parameter estimate.

References

1. Bar-Joseph, Z., Gifford, D. K. & Jaakkola, T. S. Fast optimal leaf ordering for hierarchical clustering. *Bioinformatics* **17**, S22–S29 (2001).

2. Rosenbloom, K. R. *et al.* The UCSC Genome Browser database: 2015 update. *Nucleic Acids Res.* **43**, D670-681 (2015).

3. Ernst, J. & Kellis, M. Large-scale imputation of epigenomic datasets for systematic annotation of diverse human tissues. *Nat. Biotechnol.* **33**, 364–376 (2015).

4. Davydov, E. V. *et al.* Identifying a High Fraction of the Human Genome to be under Selective Constraint Using GERP++. *PLoS Comput. Biol.* **6**, e1001025 (2010).

5. Siepel, A. *et al.* Evolutionarily conserved elements in vertebrate, insect, worm, and yeast genomes. *Genome Res.* **15**, 1034–1050 (2005).

6. Lindblad-Toh, K. *et al.* A high-resolution map of human evolutionary constraint using 29 mammals. *Nature* **478**, 476–482 (2011).

7. Garber, M. *et al.* Identifying novel constrained elements by exploiting biased substitution patterns. *Bioinformatics* **25**, i54–i62 (2009).

8. Pollard, K. S., Hubisz, M. J., Rosenbloom, K. R. & Siepel, A. Detection of nonneutral substitution rates on mammalian phylogenies. *Genome Res.* **20**, 110–121 (2010).

9. Lowe, C. B. *et al.* Three periods of regulatory innovation during vertebrate evolution. *Science* **333**, 1019–1024 (2011).

10. Iulio, J. di *et al.* The human noncoding genome defined by genetic diversity. *Nat. Genet.* **50**, 333 (2018).

11. Gronau, I., Arbiza, L., Mohammed, J. & Siepel, A. Inference of Natural Selection from Interspersed Genomic Elements Based on Polymorphism and Divergence. *Mol. Biol. Evol.* **30**, 1159–1171 (2013).
